# NUP93 facilitates the nuclear import of SOX2 to activate G3BP1 transcription and impairs gemcitabine response in pancreatic cancer

**DOI:** 10.1038/s41419-026-08586-4

**Published:** 2026-03-28

**Authors:** Hao Sun, Chenxiao Yang, Juntong Du, Chao Xu, Yao Chen, Jinsuo Chen, Nanxi Yue, Ruining Gong, Zhan Yang

**Affiliations:** 1 Department of Biochemistry and Molecular Biology, The Key Laboratory of Neural and Vascular Biology, Ministry of Education of China, Hebei Medical University, Shijiazhuang, PR China; 2 Shandong Provincial Key Laboratory of Clinical Research for Pancreatic Diseases, Tumor Immunology and Cytotherapy, Medical Research Center, The Affiliated Hospital of Qingdao University, Qingdao, PR China; 3 School of Clinical Medicine, Hebei University, Department of Urology, Affiliated Hospital of Hebei University, Baoding, PR China

**Keywords:** Pancreatic cancer, Oncogenes, Transcriptional regulatory elements, Protein transport

## Abstract

Gemcitabine is a cornerstone chemotherapeutic for pancreatic ductal adenocarcinoma (PDAC); however, the frequent development of resistance compromises its efficacy and poses a significant challenge to patient prognosis. Here, we report that nuclear pore protein NUP93 is upregulated in PDAC and correlates with poor patient survival. Functional studies demonstrated that *NUP93* promotes PDAC cell proliferation and confers gemcitabine resistance by enhancing DNA damage repair. Mechanistically, NUP93 interacts with the transcription factor SOX2 by recognizing its nuclear localization sequence and facilitates its nuclear import. Nuclear SOX2 transcriptionally activates the key stress granule component *G3BP1* by directly binding to its promoter. Subsequently, G3BP1 stabilizes the mRNA of *RAD51*, a crucial homologous recombination repair factor, thereby promoting DNA damage repair and gemcitabine resistance. In vivo, disruption of the *NUP93*/*SOX2*/*G3BP1* axis suppressed tumor growth and synergized with gemcitabine. Our findings unveil the novel *NUP93*-*SOX2*-*G3BP1* signaling axis as a critical driver of gemcitabine resistance in PDAC, presenting a promising therapeutic target for overcoming chemoresistance.

## Introduction

Pancreatic ductal adenocarcinoma (PDAC) is a highly aggressive and lethal malignancy, projected to become the second leading cause of cancer-related deaths by 2030 [1]. Although surgical resection offers a potential for cure and improved long-term survival, the majority of patients are diagnosed at advanced stages due to nonspecific early symptoms [2]. Consequently, treatment for PDAC is often limited to gemcitabine-based first-line chemotherapy [3, 4]. However, its efficacy is frequently compromised by the development of chemoresistance. A key mechanism underlying this resistance is the aberrant activation of DNA damage repair pathways in cancer cells [5, 6]. This resistance constitutes a major driver of poor outcomes in advanced PDAC, and making breakthroughs in overcoming it an urgent priority.

Gemcitabine exerts its effects by incorporating into elongating DNA strands, triggering replication stress and inducing DNA damage such as single-strand breaks [7]. The accumulation of these lesions can progress to double-strand breaks, ultimately inhibiting tumor cell proliferation [8]. Research has demonstrated that cancer cells can counteract the cytotoxic effects of gemcitabine by activating DNA damage repair systems [9, 10]. In this process, the assembly of stress granules (SGs) plays a crucial role in promoting the survival and growth of pancreatic cancer cells under stress conditions [11, 12]. Previous studies have revealed that SGs selectively protect specific mRNAs under stimulatory or cytotoxic conditions, thereby driving chemoresistance—including transcripts encoding DNA damage repair-related proteins [13, 14]. For instance, QKI7 interacts with the core SG protein G3BP1 to recruit internal m7G-modified transcripts into SGs, regulating mRNA stability and translation during stress [15]. Additionally, *DDX1* supports DNA damage repair by coordinating stress granule dynamics and maintaining the stability of key mRNAs [16]. Research by Fa-Liang Xing et al. demonstrated that *G3BP2* promotes tumor progression and gemcitabine resistance in PDAC by modulating the *PDIA3*–*DKC1*–*hENT* axis in an SG-dependent manner [12]. Although these findings highlight the significance of SG-related proteins in chemoresistance, the role of *G3BP1* in gemcitabine resistance in pancreatic cancer remains largely unexplored.

The nuclear pore complex (NPC) forms a large transmembrane channel that spans the nuclear envelope [17]. As a core structural component of the NPC, *NUP93* not only contributes to its central role in mediating nucleocytoplasmic transport [18] but is also involved in diverse cellular processes, including viral proliferation [19, 20] and the cellular stress response [21]. Moreover, mutations in *NUP93* are a known cause of hereditary nephrotic syndrome [22]. Emerging evidence has highlighted the functional importance of the NPC in DNA damage response and repair, with various nucleoporins promoting genome maintenance through distinct mechanisms. For instance, *NUP153* facilitates double-strand break repair by promoting the nuclear import of repair factors such as *53BP1* [23]; the *NUP84* complex coordinates the DNA damage response via SUMOylation to prevent the accumulation of DNA lesions [24]; and *NUP54* participates in homologous recombination to help preserve post-replicative DNA integrity [25]. While *NUP93* has been implicated in the progression of several malignancies [26–29], its specific function in pancreatic cancer, particularly in the context of DNA damage response, remains largely unexplored.

In this study, we demonstrate that *NUP93* is significantly upregulated in PDAC and correlates with poor prognosis. We have identified a novel mechanism through which NUP93 mediates the nuclear import of the transcription factor SOX2 by recognizing its NSL sequence. Subsequently, SOX2 transcriptionally activates the expression of *G3BP1*, a core stress granule component. G3BP1 stabilizes *RAD51* mRNA to enhance DNA damage repair and confer gemcitabine resistance. Our findings reveal the *NUP93*/*SOX2*/*G3BP1* axis as a critical regulator of PDAC proliferation and chemoresistance, providing a promising therapeutic target for overcoming gemcitabine resistance in this lethal disease.

## Materials and methods

### Cell lines and cell culture

The human pancreatic ductal epithelial cell line hTERT-HPNE (CL-0921; Procell) and AsPC-1 cells (CL-0027; Procell) were maintained in RPMI 1640 medium. HEK-293T (CL-0005; Procell), MIA-PACA2 (CL-0627; Procell), PANC1 (CL-0184; Procell), and SW1990 (CL-0448; Procell) cells were cultured in DMEM. Capan-1 (CL-0708; Procell) and CFPAC1 (CL-0059; Procell) were grown in IMDM. All media were supplemented with 10% fetal bovine serum (Clark Bio, Claymont, DE, USA), penicillin (100 U/mL), and streptomycin (100 µg/mL), and cells were incubated at 37 °C in a 5% CO₂ atmosphere. All shRNA constructs, control shRNA, and other reagents required for gene knockout were procured from GenePharma (Shanghai, China) and transfected following the supplier’s instructions.

### Human tissues

All patient samples were obtained from individuals treated at the Affiliated Hospital of Qingdao University. Pancreatic cancer tissues were collected from 15 patients undergoing pancreatic resection. The diagnosis of each specimen was verified by two independent pathologists. The study received approval from the Ethics Committee of the Affiliated Hospital of Qingdao University (Permission No. QYFYWZLL28017), and written informed consent was obtained from each participant.

### In vivo xenografts

A total of 5 × 10⁶ cells suspended in 100 µL of PBS were subcutaneously injected into 5-week-old male BALB/c nude mice (Weitonglihua, Beijing, China). Tumor size was measured weekly with a caliper, and tumor volume was determined using the formula: *V* = 1/2 × *L* × *W*², where *L* and *W* correspond to the tumor’s length and width, respectively. After 4 weeks, all mice were euthanized, and xenograft tumors were excised for further analysis. The Committee for the Care and Use of Laboratory Animals at the Affiliated Hospital of Qingdao University granted permission under No. AHQU-MAL20201016 [30].

### Western blot analysis

Total protein was extracted using RIPA buffer containing a protease inhibitor cocktail, as previously described. Lysate supernatants were quantified, and equal protein amounts were loaded onto gels. Following SDS-PAGE separation, proteins were electrotransferred onto polyvinylidene fluoride (PVDF) membranes (Millipore). Membranes were blocked with skim milk for 2 h and incubated with the following primary antibodies: NUP93(1:100; sc-374399), SOX2(1:1000; 11064-1-AP), G3BP1(1:5000; 66486-1-Ig), γH2AX (1:1000; ab81299), RAD51 (1:1000; 14961-1-AP). After incubation with HRP-conjugated secondary antibody (1:10,000; Rockland), membranes were treated with Chemiluminescent HRP Substrate (Millipore) and detected using a Fuazon Fx system (Vilber Lourmat) [31].

### Quantitative real-time PCR

Total RNA was extracted using the Total RNA Kit II (Omega, #R6934). Cytoplasmic and nuclear RNA fractions were isolated with the RNA Subcellular Isolation Kit (Active Motif, 25501). Nascent RNA transcripts were captured and purified using the Click-iT Nascent RNA Capture Kit (Invitrogen). RNA concentration was measured on a NanoDrop 2000 Spectrophotometer. First-strand cDNA synthesis was carried out with the M-MLV First Strand Kit (Life Technologies). Quantitative real-time PCR (qRT-PCR) was performed on an ABI 7500 FAST system using diluted cDNA and Platinum SYBR Green qPCR Super Mix UDG Kit (Invitrogen). GAPDH served as the reference gene for total RNA. Gene expression levels were calculated via the 2^−^ΔΔCt method. The primer sequences for qRT-PCR are listed as follows: *NUP93* (forward: 5’-AGTACCATCGGGAGTCAATGT-3’; reverse: 5’-TGATGTAGCTTGGCTCGCTTT-3’); *G3BP1* (forward: 5’-ACATAGCTCAGACAGTACAGGAA-3’; reverse: 5’-GCACTCTTTGATCCCGCTG-3’); *SOX2* (forward:5’-TACAGCATGTCCTACTCGCAG-3’; reverse:5’-GAGGAAGAGGTAACCACAGGG-3’); *RAD51* (forward:5’-CAACCCATTTCACGGTTAGAGC-3’; reverse:5’-TTCTTTGGCGCATAGGCAACA-3’); *GAPDH* (forward:5’-GCACCGTCAAGGCTGAGAAC-3’; reverse:5’-TGGTGAAGACGCCAGTGGA-3’); *G3BP1* promoter-site1 (forward:5’-AGGGGACGAGTTGCCTTTTA-3’; reverse:5’-CTCCAGTGGAGTGTGGGC-3’); *G3BP1* promoter-site2 (forward:5’-GAGGGCCCACACTCCACT-3’; reverse:5’-CTGAGAGTGCGCCCTTCC-3’); *G3BP1* promoter-site3 (forward:5’-GTCAGGAGTTCGAGACCAGC-3’; reverse:5’-TGCCTCAGCCTCCCTAGTAG-3’); *G3BP1* promoter-site4 (forward:5’-ACTTCCCCAAAGTACACGGG-3’; reverse:5’-GTGGGGCTGCTCTTATTCGT-3’); *G3BP1* promoter-site5 (forward:5’-CTTCAGCTCGGCCTCTCTC-3’; reverse:5’-TCTCGTTCCAGGCATGTGAC-3’) [31].

### Immunohistochemical staining and evaluation

Immunohistochemistry was conducted on 4-μm-thick paraffin-embedded tissue sections. After deparaffinization in xylene and rehydration through a graded ethanol series, sections were blocked with 10% normal goat serum (710027, KPL, USA). They were incubated with primary antibodies overnight at 4 °C, followed by incubation with horseradish peroxidase-labeled rabbit IgG secondary antibody (021516, KPL, USA). Chromogenic development was performed using a diaminobenzidine (DAB) substrate kit. Stained sections were then washed, cleared, and analyzed [32].

### Immunofluorescence staining

Cultured cells and tissue sections were permeabilized with Triton X-100, blocked with goat serum, and incubated with primary antibodies. After washing, slides were treated with fluorescein-conjugated secondary antibodies, and nuclei were stained with DAPI. Images were acquired on a Leica microscope (Leica DM6000B, Switzerland) and digitized using LAS V.4.4 software (Leica). The antibody used included Ki-67 (ab16667) [33].

### Cell proliferation assays and in vitro half maximal inhibitory concentration (IC50) assays

For proliferation assays, 3000 cells were seeded into 96-well plates, and viability was assessed over three consecutive days using the Cell Counting Kit-8 (SC-119, SEVEN, China) according to the manufacturer’s guidelines. For cytotoxic assays, cells were cultured for 24 h before treatment with varying concentrations of gemcitabine for 48 h. Absorbance was measured at 450 nm, and IC₅₀ values were determined using GraphPad Prism 9.0 [34].

### Colony formation assays

A total of 2,000 cells were plated in 6-well plates and cultured for two weeks. Cells were then washed with PBS, fixed with methanol, and stained with crystal violet. Colonies containing more than 50 cells were counted as one colony using ImageJ [34].

### 5-Ethynyl-2’-deoxyuridine (EdU) assay

The EdU assay was performed using an Edu Proliferation Kit (C0075S, Beyotime, China) according to the manufacturer’s protocol. Transfected PDAC cells were seeded in 96-well plates and cultured for 12 h. Cells were then treated with 50 µM EdU solution for 4 h, fixed with 4% paraformaldehyde, and stained with Apollo Dye Solution and Hoechst 33342. Images were captured and quantified using a Leica microscope (Leica DM6000B, Switzerland). All experiments were conducted in triplicate [30].

### Application of gemcitabine

Gemcitabine was purchased from Solarbio (Beijing, China). A working solution was prepared as per the manufacturer’s instructions and used to evaluate its effect on the proliferation of PDAC cells.

### Alkaline comet assays

DNA damage in individual cells was assessed using the alkaline comet assay. Cells were trypsinized and resuspended at 1 × 10⁶/mL. The DNA damage comet assay kit (Beyotime, C2041M) was used according to the supplied protocol. Briefly, cells were embedded in 0.7% low melting point agarose on slides precoated with 1% normal melting point agarose. After lysis and alkaline electrophoresis, samples were stained with propidium iodide (PI). Images were captured using an Olympus IX74 fluorescence microscope, and tail moment and tail DNA (%) were analyzed with CASP software [33].

### Co-immunoprecipitation (CoIP) assay

Co-IP assays were conducted using the Pierce™ Classic Magnetic IP/Co-IP Kit (Thermo, #88804) or Anti-Flag/HA Magnetic Beads (Beyotime, P2115, P2121). Cultured cells were lysed in ice-cold Lysis/Wash Buffer. Lysates were immunoprecipitated with anti-NUP93 or anti-SOX2 antibody for 1 h at room temperature. Prewashed magnetic beads were added and incubated for an additional hour. Bead–antibody–antigen complexes were collected using a magnetic stand, washed, and eluted before detection by western blot [31].

### Bioinformatics analysis

*NUP93* expression levels in various cancers, specific cancer subtypes, and corresponding normal tissues, along with clinical staging information for PDAC patients, were acquired from The Cancer Genome Atlas (TCGA) database (https://www.genome.gov/Funded-Programs-Projects/Cancer-Genome-Atlas; https://portal.gdc.cancer.gov/). The GSE283149 and GSE130656 datasets were downloaded from the Gene Expression Omnibus (GEO) database (https://www.ncbi.nlm.nih.gov/geo/). The GSE283149 dataset was used to identify downstream genes regulated by *NUP93*, while the GSE130656 dataset was used to analyze its genomic binding regions. Additionally, enhancer regions in pancreatic cancer, defined by H3K27ac and H3K4me1 histone marks, were retrieved from the EnhancerAtlas database (http://www.enhanceratlas.org/index2.php). Correlations among different genes were examined using the GEPIA 2 online tool (http://gepia2.cancer-pku.cn/#index). Analysis of *NUP93* gene effect and expression across cell lines derived from diverse tissues was performed using the Cancer Dependency Map (DepMap) database (https://depmap.org/portal/). Putative transcription factor binding sites for SOX2 within the *G3BP1* promoter region were predicted with JASPAR (http://jaspar.genereg.net/). In addition, RBPsuite (http://www.csbio.sjtu.edu.cn/bioinf/RBPsuite/) and starBase (https://rnasysu.com/encori/index.php) were employed to forecast potential binding interactions between *G3BP1* and *RAD51* mRNA.

### Molecular docking assay

The three-dimensional structures of NUP93 (UniProt ID: Q8N1F7) and SOX2 (UniProt ID: P48431) were retrieved from the Protein Data Bank (PDB) database. Molecular docking was carried out with the HDOCK Server (https://hdock.phys.hust.edu.cn) to predict the potential binding site between NUP93 and SOX2. The top-ranked binding model, selected based on the docking score, was visualized and analyzed using PyMOL software version 2.4.0 (https://www.schrodinger.com/pymol). For protein–protein interactions, docking scores below −200 are typically regarded as indicative of significant binding. Furthermore, binding confidence was classified into three tiers: high (confidence score >0.7), moderate (0.5–0.7), or low (score <0.5) [35].

### Chromatin immunoprecipitation (ChIP) assay

Cells at over 90% confluence were crosslinked with 1% formaldehyde for 10 min at 37 °C. Protein–DNA complexes were immunoprecipitated and purified using the BeyoChIP™ Kit (P2080S, Beyotime, China). Purified DNA was analyzed by qPCR [36].

### Luciferase reporter assays

Putative SOX2 binding sites within the *G3BP1* promoter region were identified via bioinformatic analysis. The *G3BP1* promoter was amplified by PCR and cloned into the pGL3-basic vector (ZB56920, Sangon, China). Firefly and Renilla luciferase activities were measured with the Dual-Luciferase Reporter Assay Kit (RG027, Beyotime, China) on a GloMax 20/20 luminometer (Promega, USA). Firefly luciferase activity was normalized to Renilla activity [36].

### RNA stability assay

To assess RNA stability, transcription was inhibited using actinomycin D (HY-17559, MCE, USA). Cells with or without *G3BP1* modulation were treated with 5 µg/mL actinomycin D and harvested at 0, 4, 8, and 12 h. RNA was extracted and analyzed by qRT-PCR [32].

### Data analysis

Data are presented as the mean ± standard deviation (SD). Statistical significance was defined as a two-sided *P*-value < 0.05. For comparisons between two independent groups with normally distributed data, an unpaired Student’s *t* test was applied. When sample sizes were small (*n* < 5) or data were not normally distributed, the non-parametric Mann-Whitney *U* test was used instead. Comparisons among three or more groups were analyzed by one-way analysis of variance (ANOVA) followed by an appropriate post hoc test. For the analysis of continuous variables measured across multiple time points, such as CCK-8 assays and tumor growth curves, a two-way repeated-measures ANOVA was employed as the required method. The sample size (*n*) for each experiment, indicating the number of independent biological replicates, is specified in the corresponding figure legend. All analyses were performed using GraphPad Prism 9.0 software.

## Results

### *NUP93* expression is upregulated in PDAC and correlates with a poor prognosis

**Fig. 1:**
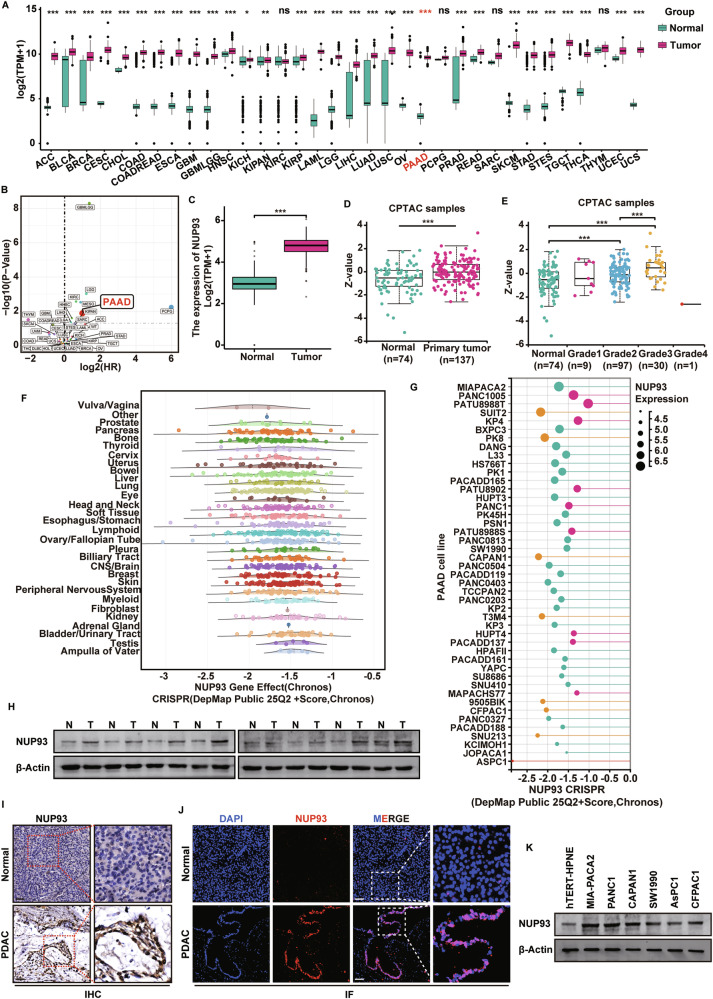
*NUP93* expression is upregulated in PDAC and correlates with a poor prognosis. **A** Comparison of *NUP93* expression status between different cancers or specific cancer subtypes and normal tissues in TCGA database. **B** Pan-cancer analysis of *NUP93*-associated survival (TCGA). High *NUP93* expression significantly correlates with poor survival in PAAD (*P* = 0.013, HR = 1.99). **C** The *NUP93* mRNA expression between PAAD tissues and normal samples in TCGA database. **D** The NUP93 protein expression between PAAD tissues and normal samples in CPTAC database. **E** The expression of NUP93 was evaluated in different groups stratified according to clinical stages. **F** Distribution of *NUP93* gene dependency scores across human cancer cell lines from the DepMap project. **G** Lollipop plot depicting *NUP93* dependency scores (Chronos) and expression levels of pancreatic cancer cell lines. **H** Western blotting of NUP93 protein expression in eight paired tumor and nontumor pancreatic tissue. **I**, **J** Representative images showing the expression of NUP93 in tumor and nontumor pancreatic tissue detected by IHC and IF staining. Red: NUP93; Blue: DAPI. Scale bar: 50 μm. **K** Western blotting of NUP93 protein expression in normal pancreatic duct epithelial cell lines and six pancreatic cancer cell lines.

To assess the expression and potential role of *NUP93* in pancreatic ductal adenocarcinoma (PDAC), we analyzed publicly available TCGA datasets. *NUP93* expression was significantly elevated in multiple tumor types and predicted poor survival (Fig. 1A, B). Further analysis specific to PDAC revealed that both mRNA and protein levels of *NUP93* were markedly higher in tumor tissues than in normal counterparts (Fig. 1C, D). Moreover, NUP93 protein levels were positively correlated with advancing pancreatic cancer stage (Fig. 1E). Interrogation of the DEPMAP database indicated that *NUP93* knockout strongly impaired the viability of nearly all cancer cell lines, with pancreatic cancer cells showing particular sensitivity (Fig. 1F, G). Western blot analysis of eight paired fresh PDAC and adjacent normal tissues further confirmed significant upregulation of NUP93 protein in tumors (Fig. 1H; Supplementary Fig. 1A). Consistent results were obtained through immunohistochemistry (IHC) and immunofluorescence analyses (Fig. 1I, J; Supplementary Fig. 1B, C). Additionally, NUP93 was aberrantly overexpressed in multiple pancreatic cancer cell lines compared to normal pancreatic ductal epithelial cells, with MIA PaCa-2 and PANC-1 cells exhibiting the highest levels (Fig. 1K; Supplementary Fig. 1D). Together, these data indicate that *NUP93* is significantly upregulated in PDAC and correlates with unfavorable prognosis.

### *NUP93* promotes pancreatic cancer cell proliferation

**Fig. 2:**
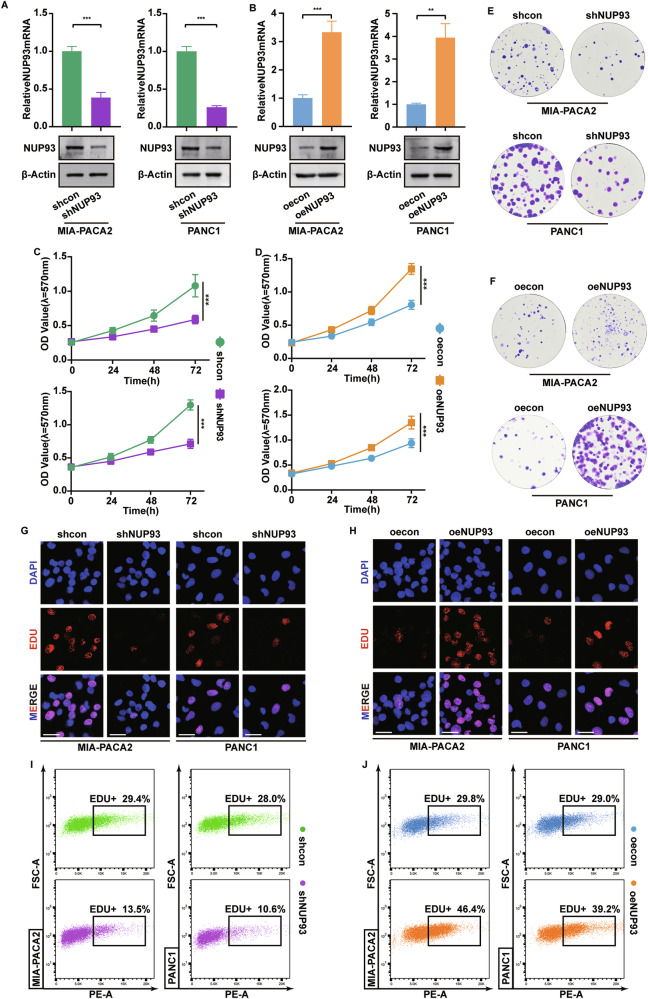
*NUP93* promotes pancreatic cancer cell proliferation. **A**, **B** The knockdown and overexpression efficiency of *NUP93* was validated at the protein and mRNA levels in MIA PACA2 and PANC1 cells (mean ± SD, ***P < 0.001, unpaired Student’s *t* test, *n* = 3). **C**, **D** Effects of *NUP93* knockdown and overexpression on cell proliferation were assessed using CCK8 assay (mean ± SD, ***P < 0.001, vs. control group, two-way repeated-measures ANOVA, *n* = 6). **E**, **F** Effects of *NUP93* knockdown and overexpression on cell proliferation were determined by colony formation assay. **G**, **H** EdU assays were performed to assess the proliferation ability of PDAC cells with *NUP93* knockdown or overexpression. Red: EDU; Blue: DAPI. Scale bar: 25 μm. **I**, **J** Flow cytometry was used to detect EdU+ cells to evaluate the proliferation changes of PDAC cells with *NUP93* knockout or overexpression.

To explore the oncogenic function of *NUP93* in PDAC, we selected MIA PaCa-2 and PANC-1 cell lines, which display high endogenous *NUP93* expression, for loss- and gain-of-function studies. Knockdown and overexpression efficiencies were verified at both mRNA and protein levels (Fig. 2A, B; Supplementary Fig. 2A–D). Functional assays revealed that *NUP93* depletion significantly reduced the proliferation and clonogenic capacity of both cell lines, whereas *NUP93* overexpression enhanced these properties (Fig. 2C-F; Supplementary Fig. 2E–H). EdU incorporation assays analyzed by immunofluorescence and flow cytometry further demonstrated that *NUP93* silencing decreased the proportion of EdU-positive cells, while its overexpression increased the number of proliferating cells (Fig. 2G–J; Supplementary Fig. 2I–L). These results collectively indicate that *NUP93* plays a critical role in promoting PDAC cell proliferation.

### *NUP93* confers gemcitabine chemoresistance to PDAC cells by enhancing DNA damage repair

**Fig. 3:**
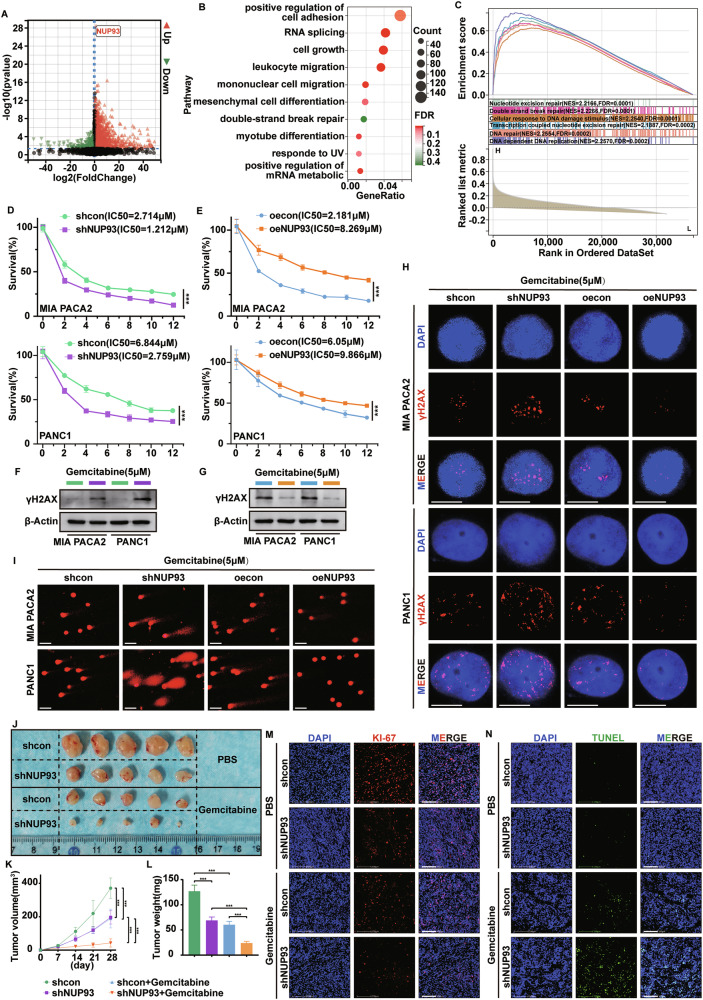
*NUP93* confers gemcitabine chemoresistance to PDAC cells by enhancing DNA damage repair. **A** Volcano plots of DEGs between the high *NUP93* expression and low *NUP93* expression group of TCGA database. The criterion for identification of DEGs is |Fold change | ≥1 and *p* < 0.05. **B** The top 10 enriched pathways were summarized. The number of genes represented in each pathway was indicated by point size, and the FDR was shown by point color. **C** GSEA enrichment analysis showed that DNA damage repair process was activated in the group with high *NUP93* expression. The high and low groups of *NUP93* were classified by the median expression of *NUP93* in PDAC specimens from the TCGA cohort. **D**, **E** PDAC cells treated with varying concentrations of gemcitabine for 48 h. Cell viability was analyzed using CCK8 assay, and IC50 values were presented (mean ± SD, ***P < 0.001, vs. control group, two-way repeated-measures ANOVA, *n* = 6). **F**, **G** Expression levels of γH2AX in PDAC cells treated with gemcitabine (5 µM) for 48 h analyzed using western blot analysis. **H** Distribution of γH2AX in PDAC cells treated with gemcitabine (5 µM) for 48 h analyzed via IF. γH2AX is stained red, and the nucleus is stained blue. Scale bar =10 μm. **I** Representative images from alkaline comet assays of control cells, *NUP93*-overexpressing cells and *NUP93* knockdown cells treated with gemcitabine (5 µM) for 48 h. Scale bar =25 μm. **J** Subcutaneous xenograft tumors were established in BALB/c nude mice (*n* = 5) using cells with or without *NUP93* knockdown, followed by intraperitoneal administration of gemcitabine (50 mg/kg). **K**, **L** Growth curve (mean ± SD, ***P < 0.001, vs. control group, two-way repeated-measures ANOVA, *n* = 5) and weight analysis (mean ± SD, ***P < 0.001, one-way ANOVA with Tukey’s post hoc test, *n* = 5) of xenograft tumors in nude mice. **M**, **N** The representative fluorescent staining images of Ki67 and TUNEL in tumor tissues of the indicated groups are shown. Red: Ki67; Green: TUNEL; Blue: DAPI. Scale bar: 100 μm.

Gemcitabine is a first-line chemotherapeutic agent for PDAC, and DNA damage repair has been implicated in gemcitabine resistance [9]. Differential gene expression analysis based on the TCGA database revealed that high *NUP93* expression was associated with biological processes, including cell proliferation, migration, DNA strand break repair, and RNA metabolism (Fig. 3A, B). Gene set enrichment analysis (GSEA) further supported a connection between *NUP93* and DNA damage repair (Fig. 3C). We therefore hypothesized that *NUP93* influences the response of PDAC cells to gemcitabine. We first evaluated gemcitabine sensitivity using CCK-8 assays in MIA PaCa-2 and PANC-1 cells. The results showed that *NUP93* knockdown enhanced PDAC cell sensitivity to gemcitabine, as reflected by decreased half-maximal inhibitory concentration (IC₅₀) values (Fig. 3D). Conversely, *NUP93* overexpression conferred increased resistance to gemcitabine, with higher IC₅₀ values (Fig. 3E). To investigate the effect of *NUP93* on gemcitabine-induced DNA damage, we assessed the levels of the DNA damage marker γH2AX by western blot and immunofluorescence (IF). *NUP93* knockdown sensitized PDAC cells to gemcitabine-induced DNA damage, whereas *NUP93* overexpression alleviated the DNA damage triggered by gemcitabine (Fig. 3 F–H; Supplementary Fig. 3A-D). In addition, alkaline comet assays demonstrated that a higher percentage of tail DNA and longer tail moments—indicators of severe DNA damage—were evident in *NUP93*-knockdown cells after 48 h of gemcitabine (5 µM) treatment. In contrast, *NUP93*-overexpressing cells exhibited shorter comet tails and reduced tail DNA percentages (Fig. 3 I; Supplementary Fig. 3E-H).

We further validated these findings in vivo using a subcutaneous xenograft model. *NUP93* knockdown significantly suppressed tumor growth and reduced tumor mass (Fig. 3J–L). Immunofluorescence analysis of proliferation marker Ki67 and apoptosis marker TUNEL revealed that *NUP93* knockdown, either alone or in combination with intraperitoneal injection of gemcitabine (50 mg/kg), markedly inhibited tumor growth and promoted apoptosis. The combination of *NUP93* knockdown and gemcitabine resulted in the most pronounced antitumor effect (Fig. 3M, N; Supplementary Fig. 3I, J). These findings demonstrate that *NUP93* enhances DNA damage repair and contributes to gemcitabine resistance in PDAC.

### *G3BP1* is involved in *NUP93*-mediated DNA damage repair to promote gemcitabine resistance in PDAC

**Fig. 4:**
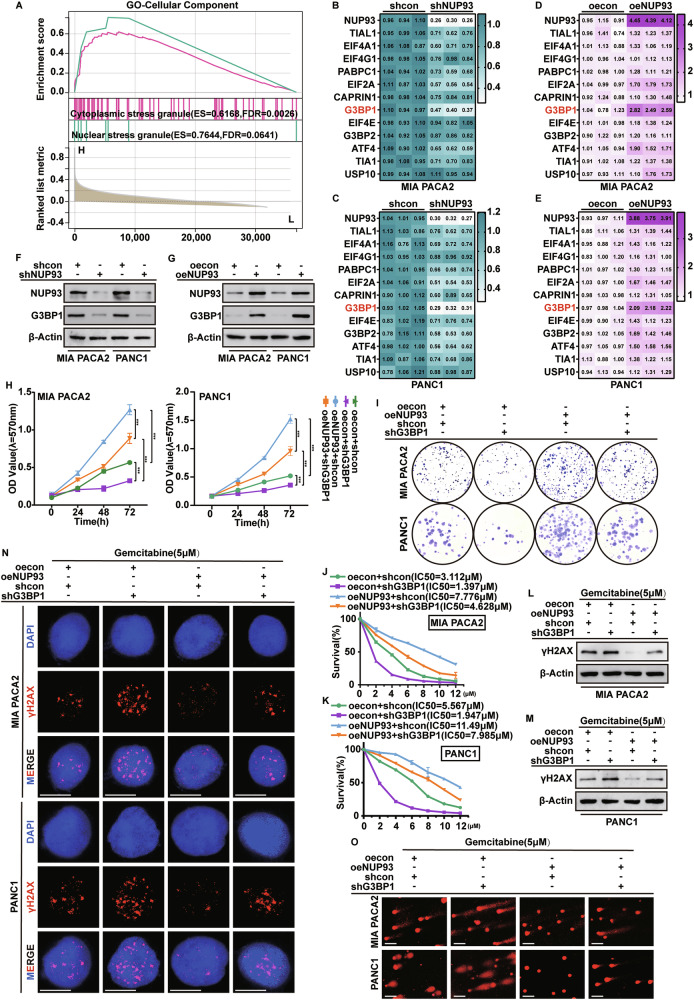
*G3BP1* is involved in *NUP93*-mediated DNA damage repair to promote gemcitabine resistance in PDAC. **A** GSEA performed using TCGA database, showing *NUP93*-related enrichment plot of cellular components. **B–E** The mRNA levels of genes encoding core cytoplasmic stress granule components were analyzed by qRT-PCR in MIA PaCa-2 and PANC-1 cells. The values in the heatmap represent relative mRNA expression normalized to *GAPDH*. **F**, **G** Western blot analysis of NUP93 and G3BP1 protein levels in MIA PaCa-2 and PANC-1 cells following *NUP93* knockdown and overexpression. **H**, **I** Cell proliferation measured by CCK-8 (mean ± SD, ***P < 0.001, vs. control group, two-way repeated-measures ANOVA, *n* = 6) and colony formation assay in MIA PaCa-2 and PANC-1 cells under individual or combined modulation of *NUP93* overexpression and *G3BP1* knockdown. **J**, **K** Viability of MIA PaCa-2 and PANC-1 cells analyzed using CCK8 assay after treatment with various concentrations of gemcitabine for 48 h, with IC50 values displayed (mean ± SD, ***P < 0.001, vs. control group, two-way repeated-measures ANOVA, *n* = 6). **L**, **M** Expression level of γH2AX in PDAC cells treated with gemcitabine (5 µM) for 48 h analyzed using western blot analysis. **N** γH2AX distribution in PDAC cells treated with gemcitabine (5 µM) for 48 h analyzed using IF. γH2AX is stained red, and the nucleus is stained blue. Scale bar = 10 μm. **O** Representative images from alkaline comet assays of oecon+shcon, oecon+shG3BP1, oeNUP93+shcon, and oeNUP93+shG3BP1 cells treated with gemcitabine (5 µM) for 48 h. Scale bar = 25 μm.

To investigate how *NUP93* mitigates gemcitabine-induced cell death, we examined its relationship with stress granule components (Fig. 4A). This correlation was further corroborated by multiplex immunofluorescence staining in PDAC patient tissues and a KPC mouse model of pancreatic carcinoma, which revealed aberrantly high expression of key stress granule markers, including G3BP1, G3BP2, TIA1, and panCK, all of which showed association with NUP93 expression (Supplementary Fig. 4A). Since stress granules help maintain cell survival by regulating RNA homeostasis, and *NUP93* was associated with RNA metabolic pathways (Supplementary Fig. 4B), we hypothesized that *NUP93* promotes resistance through stress granule-related factors. Analysis of the GSE283149 dataset indicated that *NUP93* knockout reduced levels of key stress granule proteins (Supplementary Fig. 4C). Subsequent qRT-PCR in *NUP93*-modulated cells showed that *G3BP1*, a core stress granule component, was most strongly affected (Fig. 4B–E). Database and immunohistochemical analyses confirmed a positive correlation between *NUP93* and *G3BP1* in pancreatic cancer tissues (Supplementary Fig. 4D, E), and immunoblotting verified that NUP93 upregulates G3BP1 protein (Fig. 4F, G; Supplementary Fig. 4F, G).

We next assessed whether *NUP93* promotes PDAC progression through *G3BP1* and whether *G3BP1* is involved in regulating gemcitabine sensitivity. Results from in vitro functional assays showed that *G3BP1* knockout reversed the pro-proliferative effect of *NUP93* overexpression in PDAC cells (Fig. 4H, I; Supplementary Fig. 4H). CCK-8 assays indicated that *G3BP1* knockout restored gemcitabine sensitivity in *NUP93*-overexpressing PDAC cells, as reflected by decreased IC₅₀ values (Fig. 4J, K). Western blot and immunofluorescence analyses of γH2AX levels further demonstrated that *G3BP1* ablation reversed the attenuation of gemcitabine-induced DNA damage resulting from *NUP93* overexpression (Fig. 4L-N; Supplementary Fig. 4I, J). In addition, alkaline comet assays revealed that *G3BP1* knockout significantly counteracted the DNA repair promoted by *NUP93*, with *G3BP1*-deficient cells exhibiting longer comet tails and increased tail DNA percentage (Fig. 4O; Supplementary Fig. 4K, L). These data suggest that *NUP93* upregulates *G3BP1* to enhance proliferation and DNA damage repair, thereby reducing gemcitabine sensitivity.

### NUP93 interacts with SOX2 and regulates its nuclear transport

To elucidate the molecular mechanism by which *NUP93* regulates *G3BP1* expression, we further examined the expression pattern of NUP93. Overexpressed *NUP93* effectively localized to the nucleus (Supplementary Fig. 5A, B), and its co-localization with the nuclear pore marker mAb414 indicated successful incorporation into the nuclear pore complex (Supplementary Fig. 5C). Notably, the level of NUP93 in the nucleoplasm was also markedly increased, leading us to investigate whether its accumulation in the nucleoplasm might promote *G3BP1* expression through non-canonical functions. Previous studies have reported that NUP93 can bind to gene promoters and enhancers, directly and specifically regulating gene transcription [37, 38]. Based on public ChIP-seq data (GSE130656) [39], we found that NUP93 does not bind to the *G3BP1* promoter region (Supplementary Fig. 5D). Furthermore, by comparing the four NUP93-binding regions closest to the *G3BP1* transcription start site with enhancers marked by H3K27ac and H3K4me1, we observed that the nearest potential enhancer is located more than 12,000 kb away from the *G3BP1* transcription start site (enhancer–promoter interactions typically occur within tens to hundreds of kilobases, and their frequency decreases sharply with increasing distance [40, 41]). Therefore, *NUP93* likely does not regulate *G3BP1* transcription by specifically binding to its promoter or enhancer.

**Fig. 5:**
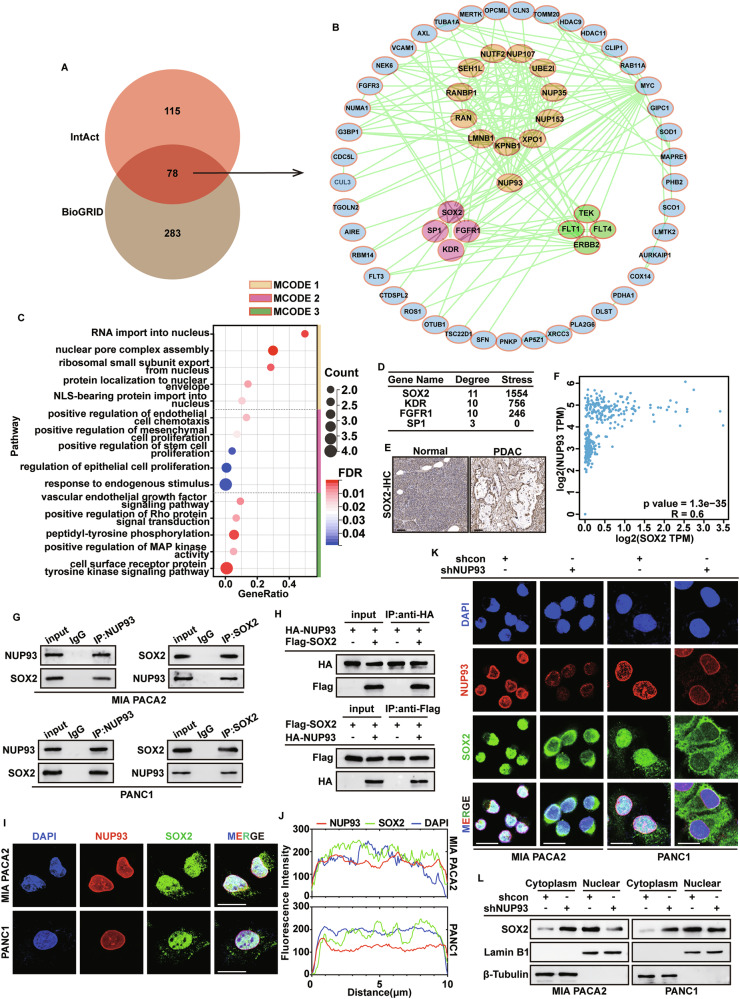
NUP93 interacts with SOX2 and regulates its nuclear transport. **A** The Venn diagram shows the interacting proteins of NUP93, and overlapping analysis with IntAct and BioGRID databases. **B** The Cytoscape visualization of the 78-gene network is overlaid with results from MCODE analysis, showing the top three modules: MCODE1 (yellow), MCODE2 (pink), and MCODE3 (green). **C** GO functional enrichment analysis of proteins in the three MCODE modules. **D** The ranking of four genes in MCODE 2 based on degree scores. **E** Representative images showing the expression of SOX2 in tumor and nontumor pancreatic tissue detected by IHC staining. Scale bar: 100 μm. **F** Correlation analysis of *NUP93* and *SOX2* expression in pancreatic cancer based on GEPIA database. **G** Co-IP assay of endogenous NUP93 and SOX2 interactions in MIA PACA2 and PANC1 cells. **H** HEK293T cells were co-transfected with Flag-SOX2 and HA-NUP93 plasmids, and exogenous NUP93-SOX2 binding was analyzed by Co-IP. **I**, **J** The localization of endogenous NUP93 and SOX2 in PDAC cell was determined by immunofluorescence assay and visualized by confocal microscopy. Red: NUP93; Green: SOX2; Blue: DAPI. Scale bar: 20 μm. **K** Immunofluorescence staining was used to determine the localization changes of SOX2 in PDAC cells after knocking down NUP93. Red: NUP93; Green: SOX2; Blue: DAPI. Scale bar: 20 μm. **L** Protein levels of SOX2 in the cytoplasm and nucleus from the indicated cells were determined using western blotting.

To further explore how *NUP93* regulates *G3BP1* transcription, we retrieved 78 predicted NUP93-interacting proteins from the InACT and BioGRID databases (Fig. 5A). A protein–protein interaction network was constructed using Cytoscape, and three key functional modules were identified via the MCODE plugin (Fig. 5B). Among these, MCODE1 contained 12 proteins primarily involved in nuclear pore complex formation and nuclear import of RNA and proteins; MCODE3 comprised four genes mainly associated with vascular endothelial growth; and MCODE2 included four proteins largely implicated in cell proliferation (Fig. 5C). Notably, among the transcription factors in MCODE2, *SOX2* occupied a central position with the highest degree score (Fig. 5D). We further analyzed *SOX2* expression in PDAC tissues and observed a significant correlation with *NUP93* levels (Fig. 5E, F). We investigated whether *NUP93* expression modulates *SOX2* levels. Similar to its effect on *G3BP1*, NUP93 likely does not act by specifically binding to the *SOX2* promoter or enhancer (Supplementary Fig. 5 E). Moreover, *NUP93* knockout data (Supplementary Fig. 5F) and subsequent RT‑PCR experiments verified that modulating *NUP93* expression has no effect on *SOX2* levels (Supplementary Fig. 5G–J). Co-immunoprecipitation assays in MIA PaCa-2 and PANC-1 cells confirmed the interaction between endogenous NUP93 and SOX2, which was further validated for exogenous prote (Fig. 5G, H). Confocal microscopy revealed nuclear co-localization of NUP93 and SOX2 (Fig. 5I, J). Given the role of NUP93 as a core component of the nuclear pore complex, we hypothesized that it may mediate the nuclear translocation of SOX2. Subsequent immunofluorescence and nuclear-cytoplasmic fractionation experiments demonstrated that *NUP93* knockdown impaired the nuclear localization of SOX2 (Fig. 5K, L). These findings indicate that NUP93 acts as a key mediator of SOX2 nuclear transport.

### NUP93 drives SOX2 nuclear import by recognizing its NSL sequence to mediate gemcitabine resistance in PDAC

**Fig. 6:**
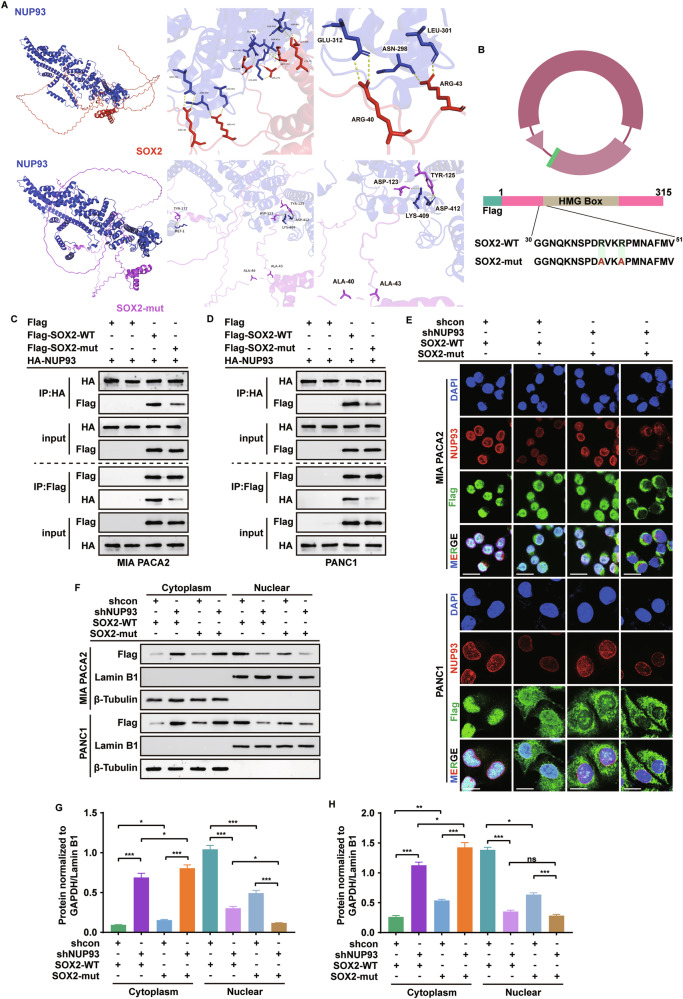
NUP93 drives SOX2 nuclear import by recognizing its NSL sequence to mediate gemcitabine resistance in PDAC. **A** Pymol visualized molecular models of the interaction between NUP93 and wild-type and mutant SOX2. **B** Pattern diagram of *SOX2* mutant. **C**, **D** PDAC cells were co-transfected with Flag-SOX2-WT/mut and HA-NUP93 plasmids, and NUP93-SOX2 binding was analyzed by Co-IP. **E** IF staining was used to determine the localization of Flag-SOX2 in PDAC cells after transfection with SOX2-WT/mut and simultaneous knockdown of NUP93. Red: NUP93; Green: Flag-SOX2; Blue: DAPI. Scale bar: 20 μm. **F–H** Protein levels of Flag-SOX2 in the cytoplasm and nucleus from the indicated cells were determined using western blotting (mean ± SD, *P < 0.05, **P < 0.01, ***P < 0.001, ns, no significance, one-way ANOVA with Tukey’s post hoc test, *n* = 3).

To further investigate the mechanism of NUP93-mediated SOX2 nuclear transport, we performed molecular docking, which revealed interactions between NUP93 and arginine residues 40 and 43 of SOX2, two residues previously identified as key components of the NSL sequence located at the N-terminal end of the HMG-box domain. Subsequent docking analyses with mutated versions of these residues indicated a weakened interaction between SOX2 and NUP93 (Fig. 6A, B). Co-immunoprecipitation experiments confirmed that the binding between NUP93 and the SOX2 mutant was substantially impaired in MIA PaCa-2 and PANC-1 cells (Fig. 6C, D). Confocal microscopy and nuclear-cytoplasmic fractionation assays further demonstrated that either *NUP93* knockdown or *SOX2* mutation significantly disrupted SOX2 nuclear localization, and their combination nearly abolished SOX2 nuclear transport (Fig. 6E–H).

To assess the functional contribution of SOX2 to NUP93-driven PDAC cell growth and gemcitabine resistance, we expressed wild-type or mutant *SOX2* in control and *NUP93*-knockdown cells. Cell viability and clonogenic assays revealed that *NUP93* knockdown, together with *SOX2* mutation, cooperatively suppressed PDAC cell growth (Supplementary Fig. 6A–D). IC₅₀ measurements indicated that this combination potently enhanced cellular sensitivity to gemcitabine (Supplementary Fig. 6E, F). Consistent with this, γH2AX detection and alkaline comet assays showed that dual disruption of *NUP93* and *SOX2* resulted in elevated DNA damage, reflected by increased γH2AX levels (Supplementary Fig. 6G–J), longer comet tail lengths, and higher tail DNA percentages (Supplementary Fig. 6K, L). These findings demonstrate that NUP93 facilitates SOX2 nuclear import via recognition of its NSL sequence, thereby promoting proliferation, DNA repair, and gemcitabine resistance.

### SOX2 transcriptionally activates *G3BP1* to promote PDAC proliferation and gemcitabine resistance

**Fig. 7:**
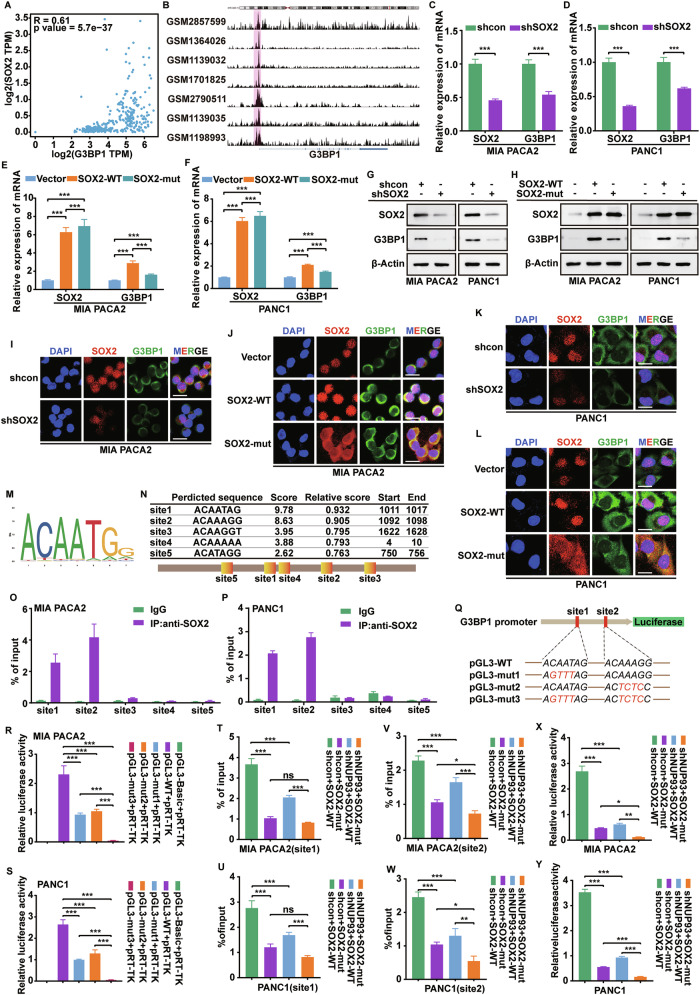
SOX2 transcriptionally activates *G3BP1* to promote PDAC proliferation and gemcitabine resistance. **A** Correlation analysis of *G3BP1* and *SOX2* expression in pancreatic cancer based on the GEPIA database. **B** UCSC Genome Browser view of SOX2 ChIP-seq enrichment at the *G3BP1* promoter region. **C**, **D** Analyze the mRNA levels of *SOX2* and *G3BP1* in MIA PaCa-2 and PANC-1 cells after knocking down *SOX2* using qRT PCR (mean ± SD, ***P < 0.001, unpaired Student’s *t* test, *n* = 3). **E**, **F** Analyze the mRNA levels of *SOX2* and *G3BP1* in MIA PaCa-2 and PANC-1 cells transfected with SOX2-WT/mut using qRT-PCR (mean ± SD, ***P < 0.001, unpaired Student’s *t* test, *n* = 3). **G**, **H** Western blot was used to detect the protein expression of SOX2 and G3BP1 in MIA PaCa-2 and PANC-1 cells after knockdown of *SOX2* and transfection with SOX2-WT/mut. **I-L** IF staining of SOX2 and G3BP1 in MIA PaCa-2 and PANC-1 cells following *SOX2* knockdown or transfection with SOX2-WT or SOX2-mut. Red: SOX2; Green: G3BP1; Blue: DAPI. Scale bar: 20 μm. **M** The DNA binding motif of SOX2 predicted by JASPAR database. **N** Prediction of SOX2-binding motifs and sites enriched in the *G3BP1* promoter using the JASPAR database. **O**, **P** ChIP-qPCR analysis was performed to determine the binding affinity of SOX2 to five *G3BP1* promoter regions in PDAC cells, showing that SOX2 bound to site1,2 regions in the *G3BP1* promoter. ChIP-qPCR with IgG was used as the negative control. **Q** Mutation patterns at sites 1 and 2 in the *G3BP1* promoter. **R**, **S** Luciferase reporter assay comparing the activity of the *G3BP1* promoter following mutation of site 1 and site 2 (mean ± SD, ***P < 0.001, Mann-Whitney *U* test, *n* = 3). **T–W** ChIP-qPCR analysis of Flag-SOX2 binding to site 1 and site 2 in the *G3BP1* promoter. Binding was assessed in MIA PaCa-2 and PANC-1 cells transfected with either wild-type (WT) or mutant (mut) SOX2, in combination with *NUP93* knockdown (mean ± SD, ***P < 0.001, Mann-Whitney U test, *n* = 3). **X**, **Y** Luciferase reporter assay measuring *G3BP1* promoter activity in MIA PaCa-2 and PANC-1 cells co-transfected with wild-type (WT) or mutant (mut) *SOX2* and subjected to *NUP93* knockdown (mean ± SD, ***P < 0.001, Mann-Whitney *U* test, *n* = 3).

We next explored whether SOX2 transcriptionally regulates *G3BP1*. Database analysis showed a positive correlation between *SOX2* and *G3BP1* in pancreatic cancer (Fig. 7A). Visualization of ChIP-seq data from the UCSC CistromeDB database revealed substantial enrichment of SOX2 at the *G3BP1* promoter region across multiple cell types (Fig. 7B). Using qRT‑PCR and western blot analysis, we found that *SOX2* knockdown suppressed, while its overexpression enhanced, *G3BP1* mRNA and protein levels in MIA PaCa-2 and PANC-1 cells. In contrast, the *SOX2* mutant exhibited a markedly diminished ability to promote *G3BP1* expression (Fig. 7C–H). Immunofluorescence analysis corroborated these results, showing a corresponding decrease in G3BP1 protein levels upon *SOX2* knockdown and an increase following wild-type *SOX2* overexpression. Notably, the upregulation of *G3BP1* expression was substantially weakened by the *SOX2* mutant, due to its impaired nuclear localization (Fig. 7I–L). We then retrieved the SOX2 target motif from the JASPAR database and predicted five potential binding sites within the *G3BP1* promoter (Fig. 7M, N). Subsequent ChIP‑PCR experiments confirmed specific binding of SOX2 to site1 and site2 (Fig. 7O, P). Luciferase reporter assays with mutated versions of these sites showed that mutation at either site1 or site2 significantly reduced promoter activity, with a particularly pronounced effect when both sites were altered (Fig. 7 Q-S). We further evaluated the binding and transcriptional activity of wild-type versus mutant *SOX2* at these promoter regions. Both *NUP93* knockdown and *SOX2* mutation impaired SOX2 binding to site1 and site2 (Fig. 7T-W) and correspondingly decreased luciferase activity (Fig. 7X, Y), with a synergistic inhibitory effect observed when both interventions were combined. These data indicate that SOX2 directly activates *G3BP1* transcription.

We next assessed the functional role of the *SOX2*/*G3BP1* axis in PDAC cell proliferation and gemcitabine resistance. Knockdown of *G3BP1* in the context of *SOX2* mutation cooperatively suppressed pancreatic cancer cell growth and clonogenicity, as determined by CCK-8 and colony formation assays (Supplementary Fig. 7A–C). IC₅₀ measurements corresponded with a pronounced increase in cellular sensitivity to gemcitabine under these conditions (Supplementary Fig. 7D, E). Furthermore, γH2AX quantification (Supplementary Fig. 7F–I) and alkaline comet assays (Supplementary Fig. 7J–L) revealed that combined *SOX2* mutation and *G3BP1* knockdown led to elevated γH2AX levels, longer comet tail lengths, and increased tail DNA percentages—collectively indicative of enhanced DNA damage. Thus, *SOX2* promotes proliferation and gemcitabine resistance by transcriptionally activating G3BP1.

### The *SOX2*-*G3BP1* axis confers gemcitabine resistance and promotes tumor growth in PDAC

Given the role of *G3BP1* as a downstream effector of *NUP93*/*SOX2*, we investigated its mechanism in gemcitabine resistance. As a core component promoting cytoplasmic stress granule formation under various stresses, G3BP1 selectively retains and facilitates the translation of pro-survival mRNAs. Given that homologous recombination repair, particularly RAD51 filament formation (Supplementary Fig. 8A), represents a crucial mechanism of gemcitabine resistance, we investigated the potential link between *G3BP1* and *RAD51*. Analysis of pancreatic cancer datasets revealed a significant correlation between *G3BP1* and *RAD51* expression (Supplementary Fig. 8 B). Through the RBPsuit database, we predicted potential binding between G3BP1 protein and *RAD51* mRNA (Supplementary Fig. 8C, D). Further analysis using the Starbase database identified the MOTIF sequence of G3BP1 binding targets and corresponding regions on *RAD51* mRNA, with three binding sites located within *RAD51* exons (Supplementary Fig. 8E, F), suggesting G3BP1 may regulate *RAD51* RNA stability and expression. Experimentally, *G3BP1* knockdown in MIA PaCa-2 and PANC-1 cells significantly reduced both *RAD51* mRNA and protein levels (Supplementary Fig. 8G, H). RNA stability assays demonstrated that G3BP1 depletion accelerated *RAD51* mRNA degradation (Supplementary Fig. 8I). Furthermore, RNA immunoprecipitation (RIP) experiments confirmed direct binding between G3BP1 and *RAD51* mRNA (Supplementary Fig. 8 J). These findings indicate that G3BP1 promotes gemcitabine resistance by stabilizing *RAD51* mRNA and facilitating homologous recombination repair.

**Fig. 8:**
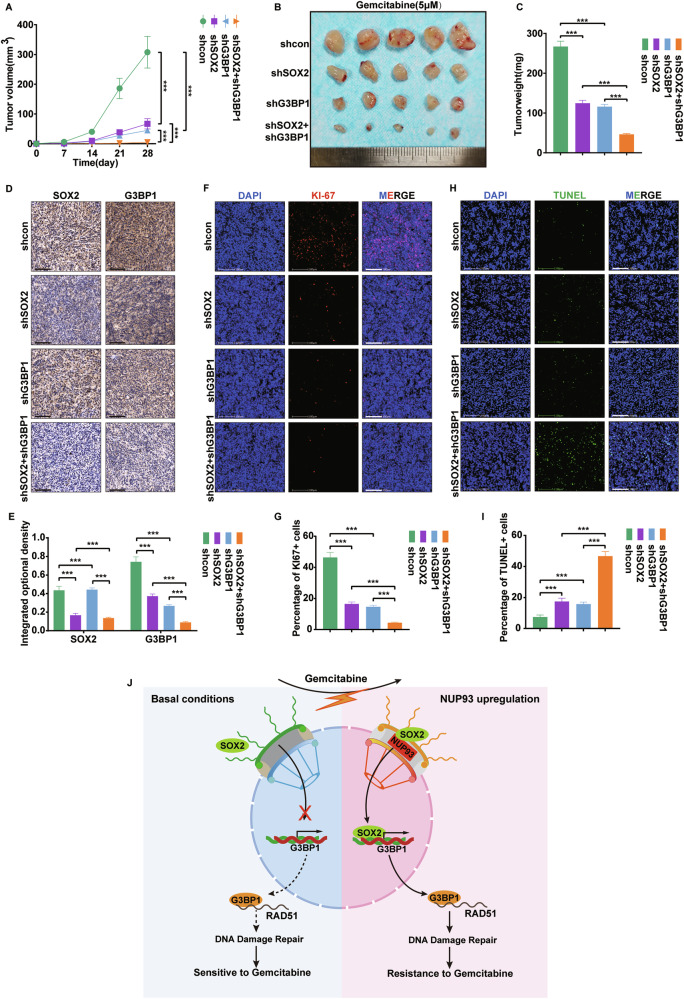
The *SOX2*-*G3BP1* axis confers gemcitabine resistance and promotes tumor growth in PDAC. **A** Growth curve analysis of xenograft tumor in nude mice (mean ± SD, ***P < 0.001, vs. control group, two-way repeated-measures ANOVA, *n* = 5). **B** Subcutaneous xenograft model with combinatorial(50 mg/kg) *SOX2*/*G3BP1* knockdown and gemcitabine treatment in BALB/c nude mice (*n* = 5 per group). **C** Weight analysis of xenograft tumors in nude mice (mean ± SD, ***P < 0.001, one-way ANOVA with Tukey’s post hoc test, *n* = 5). **D**, **E** Representative images and integrated optional density of SOX2 and G3BP1 IHC staining in different groups of xenograft tumor tissues. Scale bar: 100 μm. (mean ± SD, ***P < 0.001, Mann-Whitney U test, *n* = 3) **F**, **G** Representative images of Ki-67 IF staining in different groups of xenograft tumor tissues. Red: Ki67; Blue: DAPI. Scale bar: 100 μm. (mean ± SD, ***P < 0.001, Mann-Whitney *U* test, *n* = 3) **H, I** Representative images of TUNEL IF staining in different groups of xenograft tumor tissues. Green: TUNEL; Blue: DAPI. Scale bar: 100 μm. (mean ± SD, ***P < 0.001, Mann-Whitney *U* test, *n* = 3). **J** Schematic diagram of the mechanism by which the *NUP93*-*SOX2* axis promotes tumor growth and confers gemcitabine resistance in pancreatic cancer.

To validate the in vivo role of the *SOX2*/*G3BP1* axis in PDAC growth and gemcitabine resistance, we established xenograft models using PANC-1 cells. Both *SOX2* and *G3BP1* knockdown significantly suppressed tumor growth rates, volume, and weight, while synergistically enhancing sensitivity to gemcitabine (Fig. 8A–C). Immunohistochemical analysis demonstrated that *SOX2* knockdown reduced G3BP1 expression (Fig. 8D, E) and inhibited tumor cell proliferation, as measured by Ki-67 staining (Fig. 8F, G). *G3BP1* knockdown further potentiated the cytotoxic effects of gemcitabine chemotherapy (Fig. 8H, I). In summary, NUP93-mediated SOX2 nuclear translocation transcriptionally activates *G3BP1*, which stabilizes *RAD51* mRNA to facilitate homologous recombination repair, thereby promoting PDAC proliferation and gemcitabine resistance (Fig. 8J).

## Discussion

Our study reveals that *NUP93* is overexpressed in PDAC and associated with poor patient outcomes. We further establish a novel function for *NUP93* in driving tumorigenesis and conferring gemcitabine resistance through enhanced DNA damage repair. Mechanistically, we propose a model whereby NUP93 binds to the NSL motif of SOX2, promoting its nuclear accumulation. This event triggers *G3BP1* transcription and activates the homologous recombination repair pathway, ultimately protecting cells from gemcitabine. Therapeutically, *NUP93* inhibition effectively curbs tumor growth and sensitizes PDAC to gemcitabine.

*NUP93*, together with *NUP205*, *NUP188*, *NUP155*, and *NUP35*, constitutes the inner ring complex of the nuclear pore [42]. Mutations in *NUP93* are known to cause steroid-resistant nephrotic syndrome [22]. Numerous studies have reported that *NUP93* is overexpressed in multiple malignancies, where it promotes cancer cell proliferation and metastasis. Recent research has further revealed its tumor-promoting mechanisms in specific contexts. For instance, in liver cancer, NUP93 enhances the phosphorylation of β-catenin at the S675 site and facilitates its nuclear accumulation, which in turn promotes *NUP93* transcription by binding to the transcription factor LEF1, forming a positive feedback loop [27]. In breast cancer, NUP93 leverages its robust nucleocytoplasmic transport capacity to activate MYC, thereby driving tumor progression [26]. In addition, Bin Chen et al. reported that NUP93 mediates the nuclear translocation of the mitochondrial factor HIGD1A upon DNA damage, thereby regulating homologous recombination and cellular sensitivity to radiation and chemicals [43]. Beyond regulating antiviral immunity [44], *NUP93* is linked to immunosuppression and poor prognosis in clear cell renal cell carcinoma via an IFN-γ response signature [45]. Its capacity to activate macrophage and T-cell signaling, as shown by Manish et al. [46], highlights its broader role in immune modulation and its potential in reshaping the tumor immune microenvironment in diverse cancers. In this study, we found that *NUP93* is overexpressed in PDAC and correlates with poor prognosis. We further demonstrated that *NUP93* promotes tumor growth and gemcitabine resistance in PDAC by upregulating the expression of *RAD51*—a key executor of homologous recombination repair—thereby enhancing the DNA damage repair response [47]. Given its strong association with malignancy, elucidating the precise mechanism by which *NUP93* activates DNA damage repair may offer therapeutic strategies to inhibit tumor growth and restore gemcitabine sensitivity.

The transcription factor *SOX2* collaborates with various co-factors to orchestrate cell-type-specific gene expression programs that underlie pluripotency, thereby finely regulating cell fate [48]. Previous studies have revealed the role of *SOX2* in mediating chemotherapy resistance across multiple cancers. The work by Sanjit et al. suggested that *SOX2* contributes to drug resistance in pancreatic tumor organoids [49]. Consistent with our findings, research by Jia et al. indicated that reduced *SOX2* expression effectively sensitizes pancreatic cancer cells to gemcitabine treatment [50]. In embryonic stem cells, *SOX2* interacts directly with the XPC nucleotide excision repair complex, recruiting it to specific genomic loci, thereby playing a dual role in transcriptional regulation and DNA repair, which is crucial for maintaining pluripotency and genomic integrity [51, 52]. Our study further demonstrates that the nuclear localization of SOX2 is mediated by NUP93, which in turn activates *G3BP1* expression, leading to elevated *RAD51* levels and consequently promoting DNA damage repair and gemcitabine resistance.

Chemotherapy resistance in pancreatic cancer represents a major obstacle in anticancer treatment, with underlying molecular mechanisms that are complex and multifactorial. Recent studies have proposed a potential novel strategy to overcome chemotherapy resistance by targeting the PI3K/Akt pathway [53]. Another study demonstrated that FTO demethylates *NEDD4* RNA in an m6A-dependent manner, which subsequently modulates *PTEN* expression levels. This alteration in turn affects the PI3K/AKT signaling pathway and ultimately influences the chemosensitivity of pancreatic cancer cells to gemcitabine [54]. The homologous recombination repair (HR) pathway, which is the focus of our research, plays an especially critical role in gemcitabine resistance in pancreatic cancer [8, 55]. Several studies have indicated that both m6A methylation modifications [56, 57] and the PI3K/AKT pathway [58, 59] can reduce chemosensitivity by affecting homologous recombination repair. *RAD51*, as a core component of the HR pathway, forms nuclear foci at DNA damage sites and mediates high-fidelity DNA repair [60, 61]. Aberrant *RAD51*-mediated repair has been recognized as a key driver of gemcitabine resistance, contributing to chemotherapy failure across multiple cancer types [62, 63]. Conversely, impairing homologous recombination or knocking down *RAD51* has been shown to suppress tumor progression and improve outcomes in cancer patients [10, 64]. However, the upstream regulatory mechanisms controlling *RAD51* expression in pancreatic cancer remain incompletely understood. This mechanism suggests a potential explanation for the sustained high expression of *RAD51* in pancreatic cancer and implies a novel pathway through which stress granules may influence chemotherapy sensitivity by regulating key DNA repair genes.

While the present findings systematically elucidate the critical role of NUP93 in mediating nuclear transport of SOX2 and its downstream *G3BP1*/*RAD51* pathway in gemcitabine resistance in pancreatic cancer, several aspects merit further investigation. The cell lines and subcutaneous xenograft mouse models employed in this work may not fully recapitulate the complex interactions between *NUP93* and the tumor microenvironment in patients, including stromal and immune components along with biomechanical factors. Future studies would benefit from exploring more clinically relevant systems, such as patient-derived organoids or immunocompetent pancreatic cancer mouse models, to validate and extend these findings. Additionally, as a core component of the nuclear pore complex, the substrate selectivity and specificity of *NUP93* remain to be elucidated in future studies. In addition, the molecular mechanisms governing its precise assembly into the nuclear pore under varying physiological or pathological conditions, as well as its noncanonical, moonlighting functions independent of classical nucleocytoplasmic transport, warrant further investigation [38]. Beyond its demonstrated role in facilitating SOX2 nuclear import, it would be valuable to systematically examine whether NUP93 participates in nuclear transport of other transcription factors involved in DNA damage repair or chemotherapy response. Furthermore, although this work focused on the function of G3BP1 in stabilizing *RAD51* mRNA to promote homologous recombination repair, G3BP1 remains a core stress granule protein with potential multifaceted roles. Under gemcitabine treatment conditions, whether G3BP1 influences chemotherapy-resistant phenotypes through regulation of other RNA targets or via non-RNA-dependent mechanisms represents a promising direction for further exploration. A deeper understanding of these aspects could provide a more comprehensive perspective on the multifunctional nature of G3BP1 in chemoresistance.

In conclusion, the current study reveals a previously uncharacterized role for the *NUP93*-*SOX2*-*G3BP1* signaling axis in promoting pancreatic ductal adenocarcinoma (PDAC) proliferation and chemoresistance. We demonstrate that *NUP93*, which is frequently upregulated in PDAC and correlates with poor prognosis, interacts with and facilitates the nuclear import of the transcription factor SOX2 by recognizing its nuclear localization sequence. Nuclear SOX2 in turn transcriptionally activates the stress granule core protein *G3BP1*, which post-transcriptionally stabilizes *RAD51* mRNA to enhance homologous recombination repair, thereby sustaining DNA damage repair and conferring gemcitabine resistance. These findings not only delineate a cohesive signaling pathway driving PDAC progression and therapy resistance but also suggest that targeting the *NUP93*-*SOX2*-*G3BP1* axis could represent a promising therapeutic strategy to suppress tumor growth and overcome gemcitabine resistance in PDAC.

## Supplementary information

Supplementary Figure

Original Western blots

## Data Availability

All data are included within the main text and supplementary files.
