# Supplementary material for: NUP93 facilitates the nuclear import of SOX2 to activate G3BP1 transcription and impairs gemcitabine response in pancreatic cancer
Source: Cell Death Dis. 2026 Mar 28;17(1):423. doi: 10.1038/s41419-026-08586-4 (PMC13149976; doi:10.1038/s41419-026-08586-4)
Supplement: Supplementary file 1 — Supplementary Figure [file 41419_2026_8586_MOESM1_ESM.docx]

**Supplemental Figure**

**Supplemental Fig.1**

**
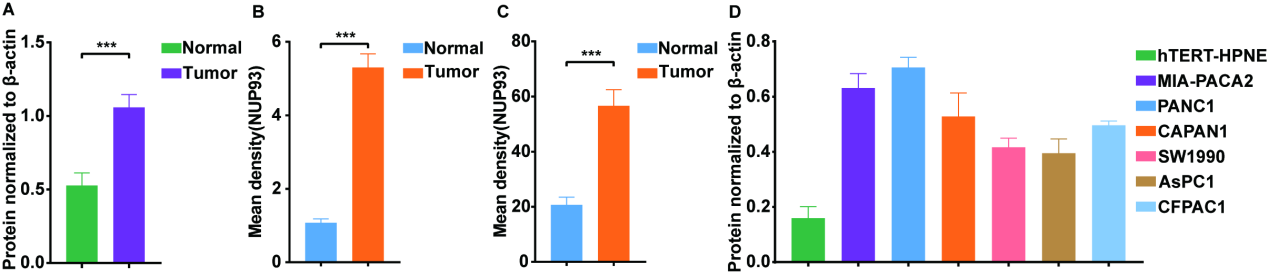
**

**Supplemental Fig.1** **A** Densitometry analysis of Western blot bands from Fig. 1H. **B** Comparison of the average NUP93 IHC staining intensity between PDAC and matched adjacent non-tumor tissues from Fig. 1I. **C** Comparison of average fluorescence intensity of NUP93 in Fig 1J. **D** Densitometry analysis of Western blot bands from Fig. 1K.(mean ± SD, ***P < 0.001, Mann-Whitney U test, n = 3)

**Supplemental Fig.2**


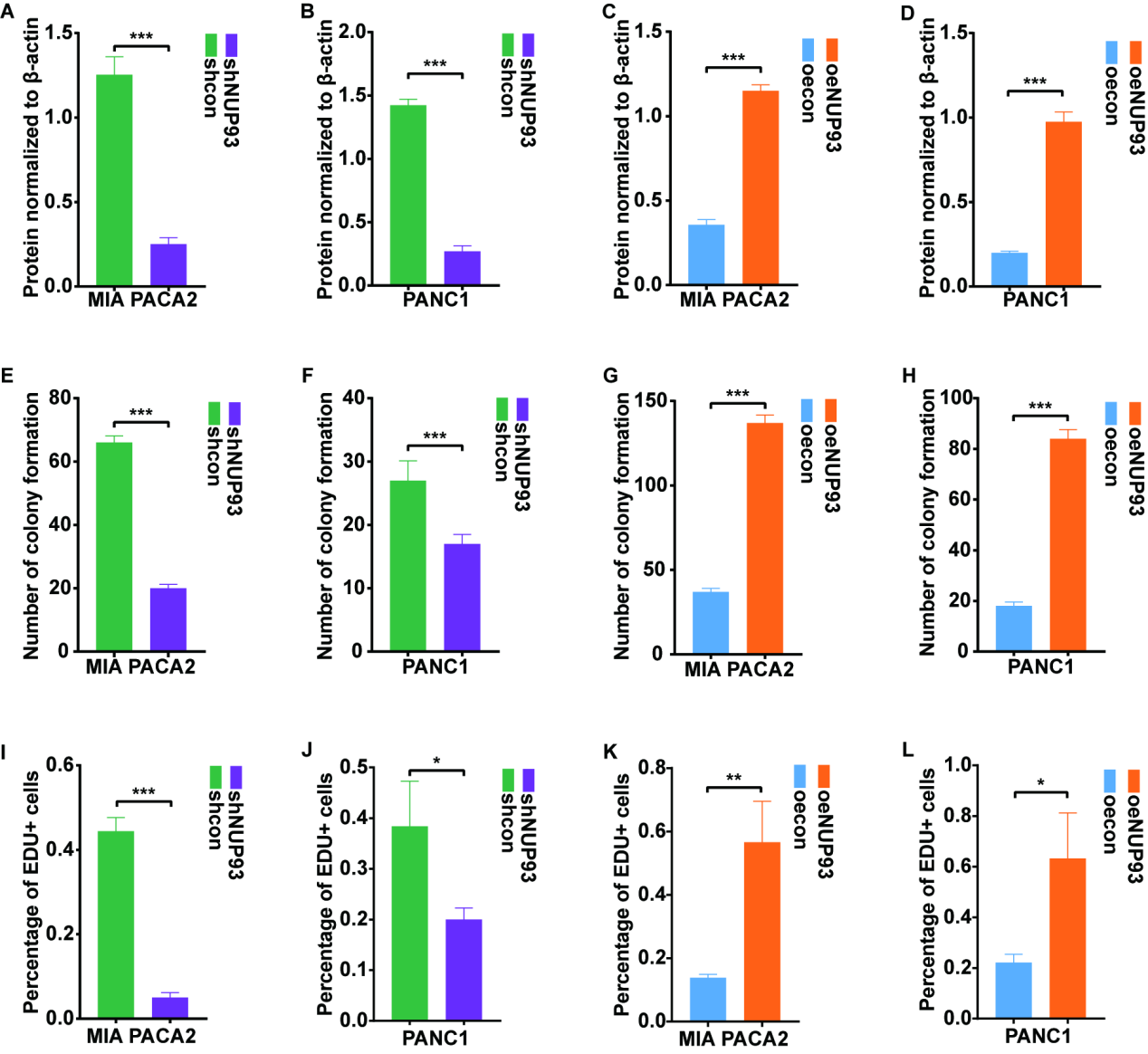


**Supplemental Fig.2** **A-D** Densitometry analysis and comparison of Western blot bands from Fig 2A,B. **E-H** Compare the number of clones formed before and after knocking down or overexpressing *NUP93* in Fig 2E, F. **I-L** Compare the percentage of EDU positive cells before and after knocking down or overexpressing *NUP93* in Fig 2G,H.(mean ± SD, *P < 0.05, ***P < 0.001, Mann-Whitney U test, n = 3)

**Supplemental Fig.3**


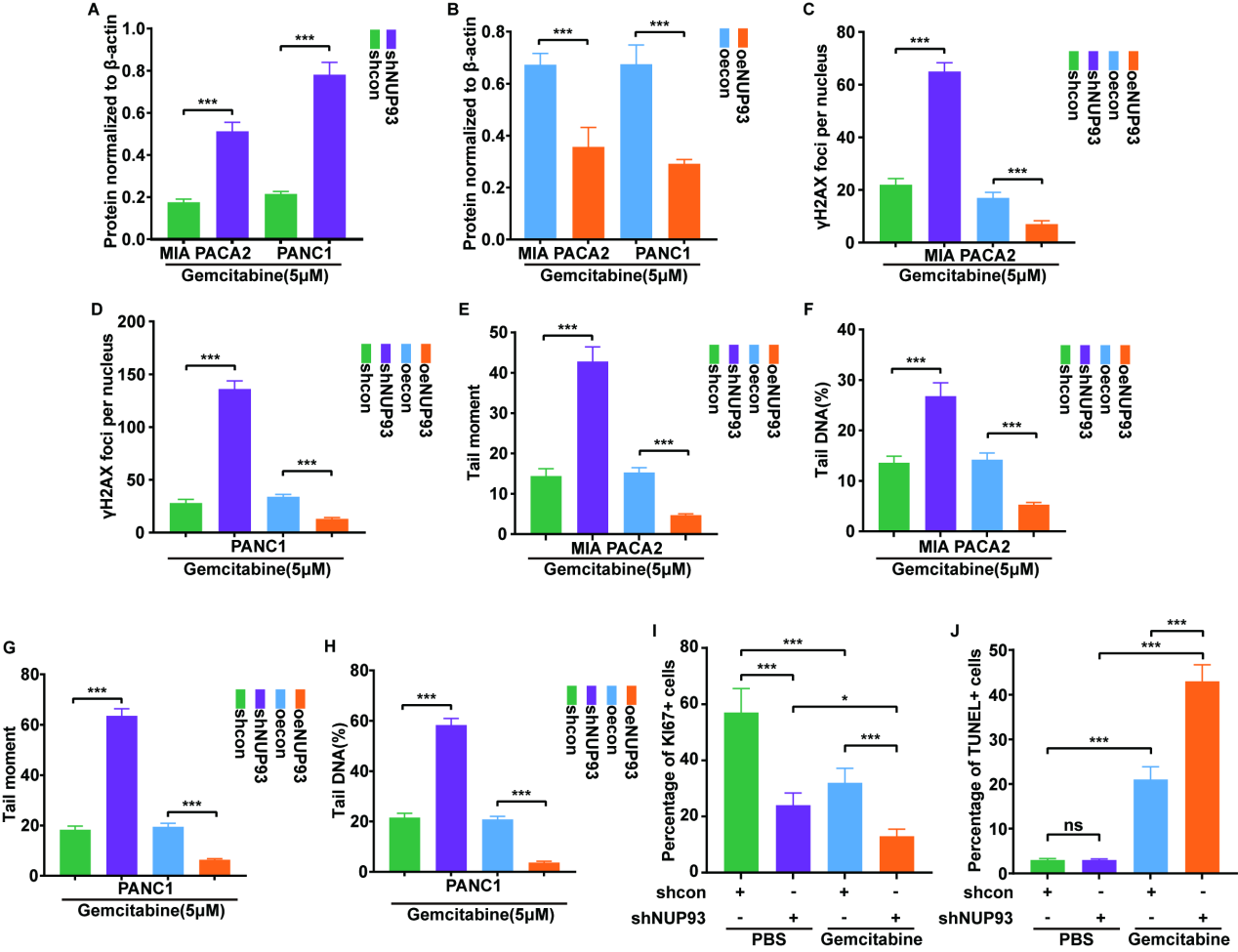


**Supplemental Fig.3** **A,B** Densitometry analysis of Western blot bands from Fig.3 F,G. **C,D** Count comparison and comparison of γH2AX focal counts before and after knocking down or overexpressing *NUP93* in Fig 3H.(mean ± SD, *P < 0.05, **P < 0.01, ***P < 0.001, ns, no significance, one-way ANOVA with Tukey’s post hoc test, n =20) **E-H** Count comparison of comet tail length and tail DNA percentage before and after knocking down or overexpressing *NUP93* in Fig 3I. **I** The percentage statistics and comparison of Ki-67 positive cells in different groups of samples in Fig 3M. **J** The percentage statistics and comparison of TUNEL positive cells in different groups of samples in Figure 3N. (mean ± SD, *P < 0.05, **P < 0.01, ***P < 0.001, ns, no significance, one-way ANOVA with Tukey’s post hoc test, n =3)

**Supplemental Fig.4**


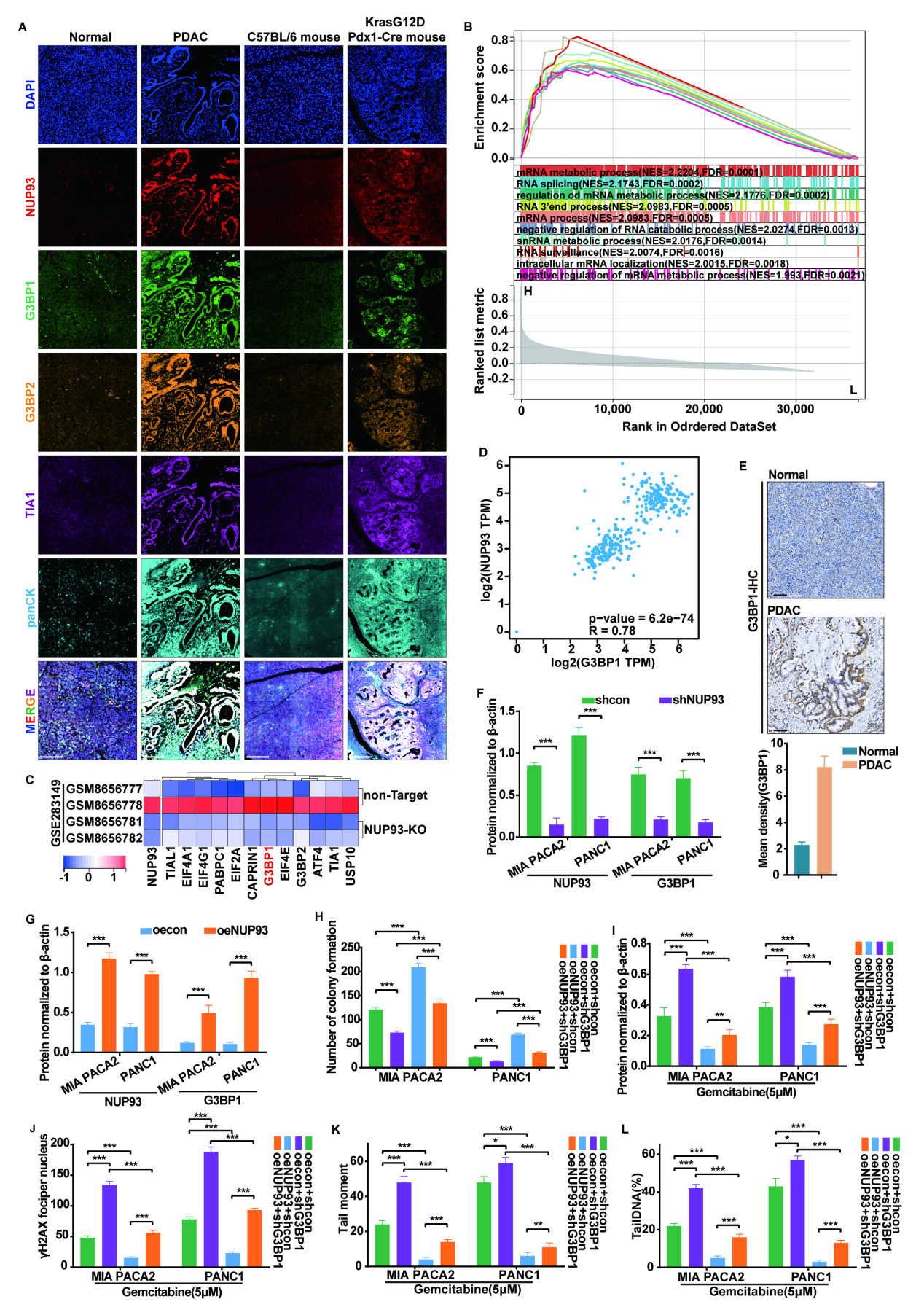


**Supplemental Fig.4** **A** Representative multiplex immunofluorescence images showing the co-localization of NUP93, G3BP1, G3BP2, TIA1, and panCK in pancreatic ductal adenocarcinoma (PDAC) tissues and KPC mouse pancreatic tumors. Red: NUP93; Green: G3BP1; Orange: G3BP2; Purple: TIA1; Cyan: pan-CK; Blue: DAPI. Scale bar, 100 μm. **B** GSEA performed using TCGA database, showing *NUP93*-related enrichment plot of RNA metabolism-related pathways. **C** The heatmap shows the relative expression of key stress granule proteins before and after *NUP93* knockout in GSE283149. **D** Correlation analysis of *NUP93* and *G3BP1* expression in pancreatic cancer based on GEPIA database. **E** Representative images showing the expression of G3BP1 in tumor and nontumor pancreatic tissue detected by IHC staining. Scale bar: 100 μm.(mean ± SD, *P < 0.05, ***P < 0.001, Mann-Whitney U test, n = 3) **F,G** Densitometry analysis of Western blot bands from Fig. 4F,G (mean ± SD, *P < 0.05, ***P < 0.001, Mann-Whitney U test, n = 3). **H,I** Compare the number of clones formed after knocking down *G3BP1* and overexpressing *NUP93* in Fig 4I. **J** Count comparison and comparison of γH2AX focal counts in Fig 4N. **K,L** Count comparison of comet tail length and tail DNA percentage in Fig 3O. (mean ± SD, *P < 0.05, **P < 0.01, ***P < 0.001, ns, no significance, one-way ANOVA with Tukey’s post hoc test, n =3)

**Supplemental Fig.5**


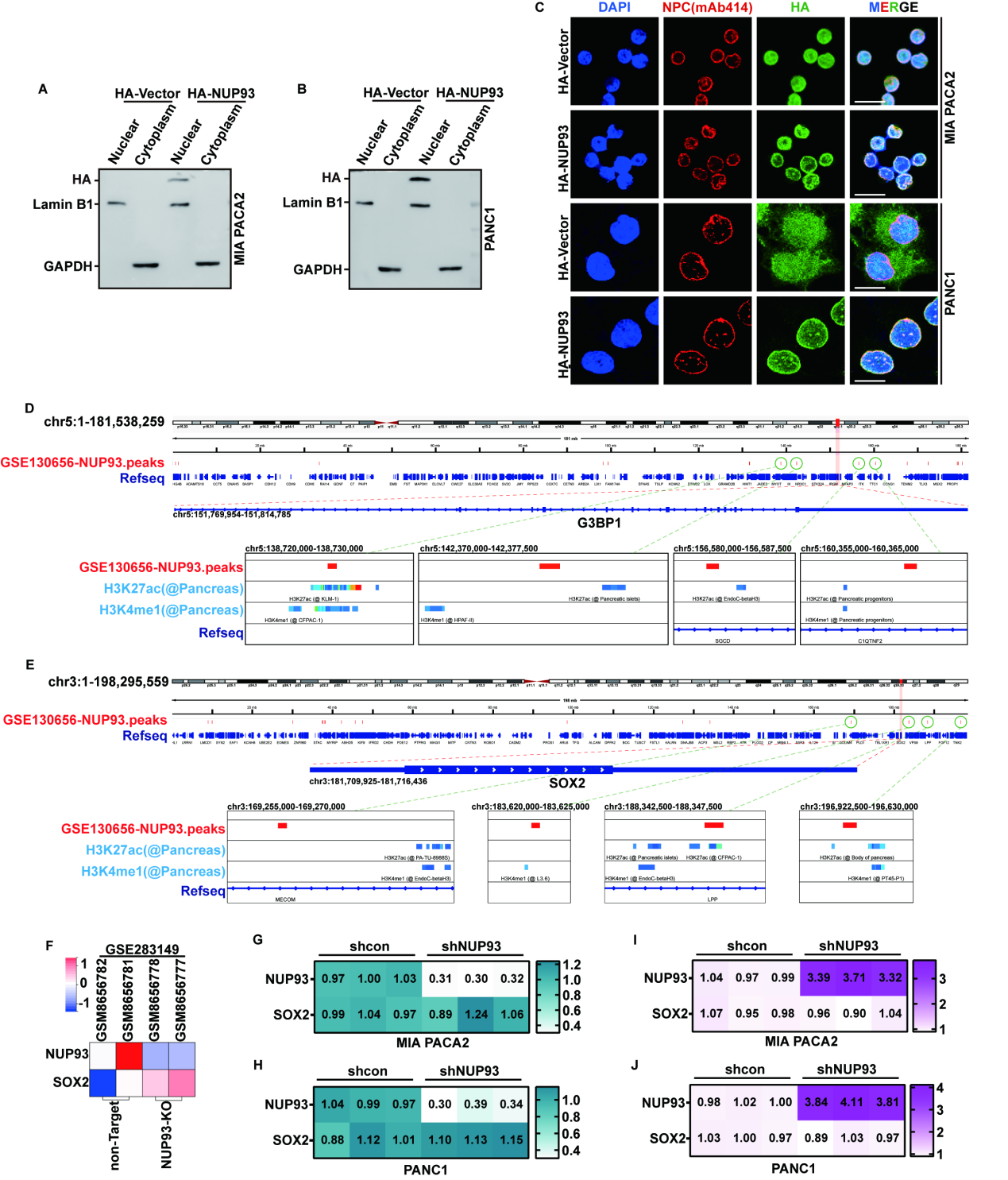


**Supplemental Fig.5 A,B** Western blot analysis of HA-NUP93 expression in nuclear and cytoplasmic fractions following subcellular fractionation. **C** Immunofluorescence staining showing the localization of HA-NUP93 and mAb414. **D,E** IGV visualization comparing the genomic tracks of NUP93 ChIP-seq (GSE130656) with enhancer regions marked by H3K27ac and H3K4me1 in pancreatic cancer. **F** The heatmap shows the relative expression of key stress granule proteins before and after *NUP93* knockout in GSE283149. **G-J** The mRNA levels of *NUP93* and *SOX2* were analyzed by qRT-PCR in MIA PaCa-2 and PANC-1 cells. The values in the heatmap represent relative mRNA expression normalized to *GAPDH*.

**Supplemental Fig.6**


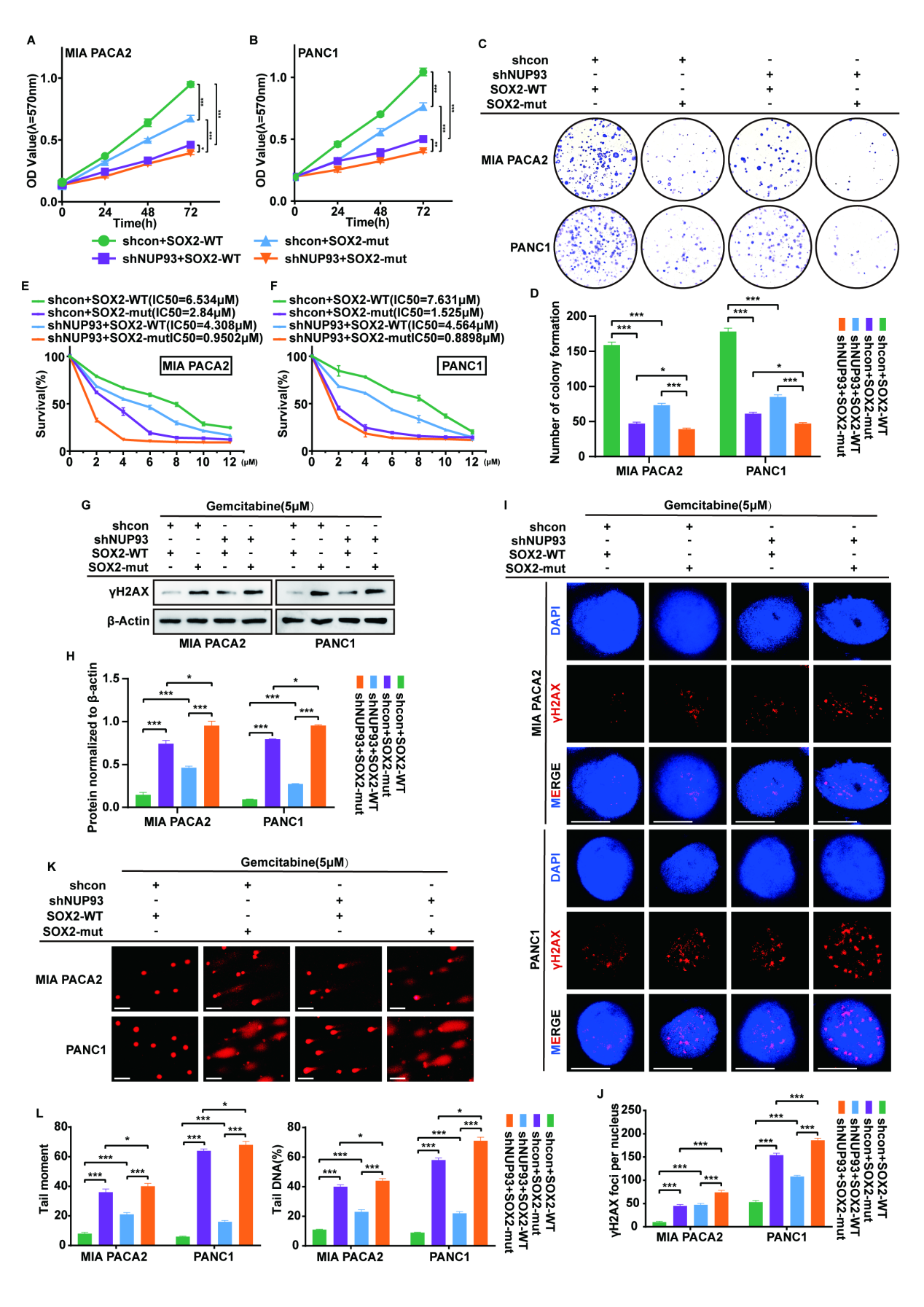


**Supplemental Fig.6** **A-D** Cell proliferation measured by CCK-8  (mean ± SD, ***P < 0.001, vs. control group, two-way repeated-measures ANOVA, n = 6) and colony formation assay (mean ± SD, *P < 0.05, **P < 0.01, ***P < 0.001, ns, no significance, one-way ANOVA with Tukey’s post hoc test, n =3) in MIA PaCa-2 and PANC-1 cells after transfection with *SOX2*-WT/mut and simultaneous knockdown of *NUP93*. **E,F** Viability of MIA PaCa-2 and PANC-1 cells analysed using CCK8 assay after treatment with various concentrations of gemcitabine for 48 h, with IC50 values displayed (mean ± SD, ***P < 0.001, vs. control group, two-way repeated-measures ANOVA, n = 6). **G,H** Expression level of γH2AX in PDAC cells treated with gemcitabine (5 µM) for 48h analysed using western blot analysis (mean ± SD, *P < 0.05, **P < 0.01, ***P < 0.001, ns, no significance, one-way ANOVA with Tukey’s post hoc test, n =3). **I,J** γH2AX distribution in PDAC cells treated with gemcitabine (5 µM) for 48h analysed using IF. γH2AX is stained red, and the nucleus is stained blue. Scale bar=20μm.(mean ± SD, *P < 0.05, **P < 0.01, ***P < 0.001, ns, no significance, one-way ANOVA with Tukey’s post hoc test, n =20) **K,L** Representative images from alkaline comet assays and count comparison of comet tail length and tail DNA percentage ,after transfection with *SOX2*-WT/mut and simultaneous knockdown of *NUP93*.(mean ± SD, *P < 0.05, **P < 0.01, ***P < 0.001, ns, no significance, one-way ANOVA with Tukey’s post hoc test, n =3)

**Supplemental Fig.7**


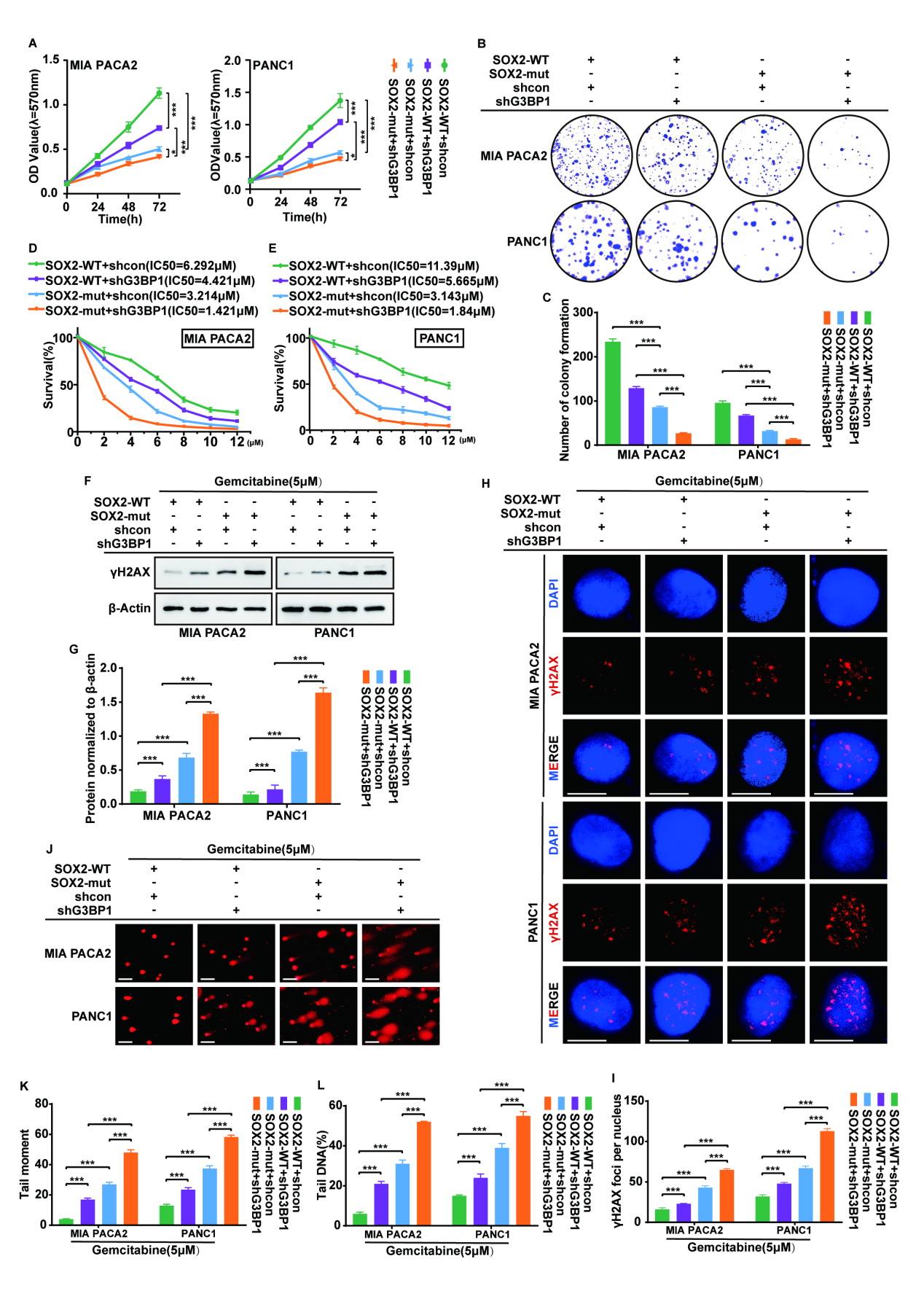


**Supplemental Fig.7** **A-C** Cell proliferation measured by CCK-8 (mean ± SD, ***P < 0.001, vs. control group, two-way repeated-measures ANOVA, n = 6) and colony formation assay (mean ± SD, *P < 0.05, **P < 0.01, ***P < 0.001, ns, no significance, one-way ANOVA with Tukey’s post hoc test, n =3) in MIA PaCa-2 and PANC-1 cells after transfection with *SOX2*-WT/mut and simultaneous knockdown of *G3BP1*. **D,E** Viability of MIA PaCa-2 and PANC-1 cells analysed using CCK8 assay after treatment with various concentrations of gemcitabine for 48 h, with IC50 values displayed (mean ± SD, ***P < 0.001, vs. control group, two-way repeated-measures ANOVA, n = 6). **F,G** Expression level of γH2AX in PDAC cells treated with gemcitabine (5 µM) for 48h analysed using western blot analysis (mean ± SD, *P < 0.05, **P < 0.01, ***P < 0.001, ns, no significance, one-way ANOVA with Tukey’s post hoc test, n =3). **H,I** γH2AX distribution in PDAC cells treated with gemcitabine (5 µM) for 48h analysed using IF. γH2AX is stained red, and the nucleus is stained blue. Scale bar=20μm.(mean ± SD, *P < 0.05, **P < 0.01, ***P < 0.001, ns, no significance, one-way ANOVA with Tukey’s post hoc test, n =20) **J-L** Representative images from alkaline comet assays and count comparison of comet tail length and tail DNA percentage ,after transfection with *SOX2*-WT/mut and simultaneous knockdown of *G3BP1*.(mean ± SD, *P < 0.05, **P < 0.01, ***P < 0.001, ns, no significance, one-way ANOVA with Tukey’s post hoc test, n =3)

**Supplemental Fig.8**


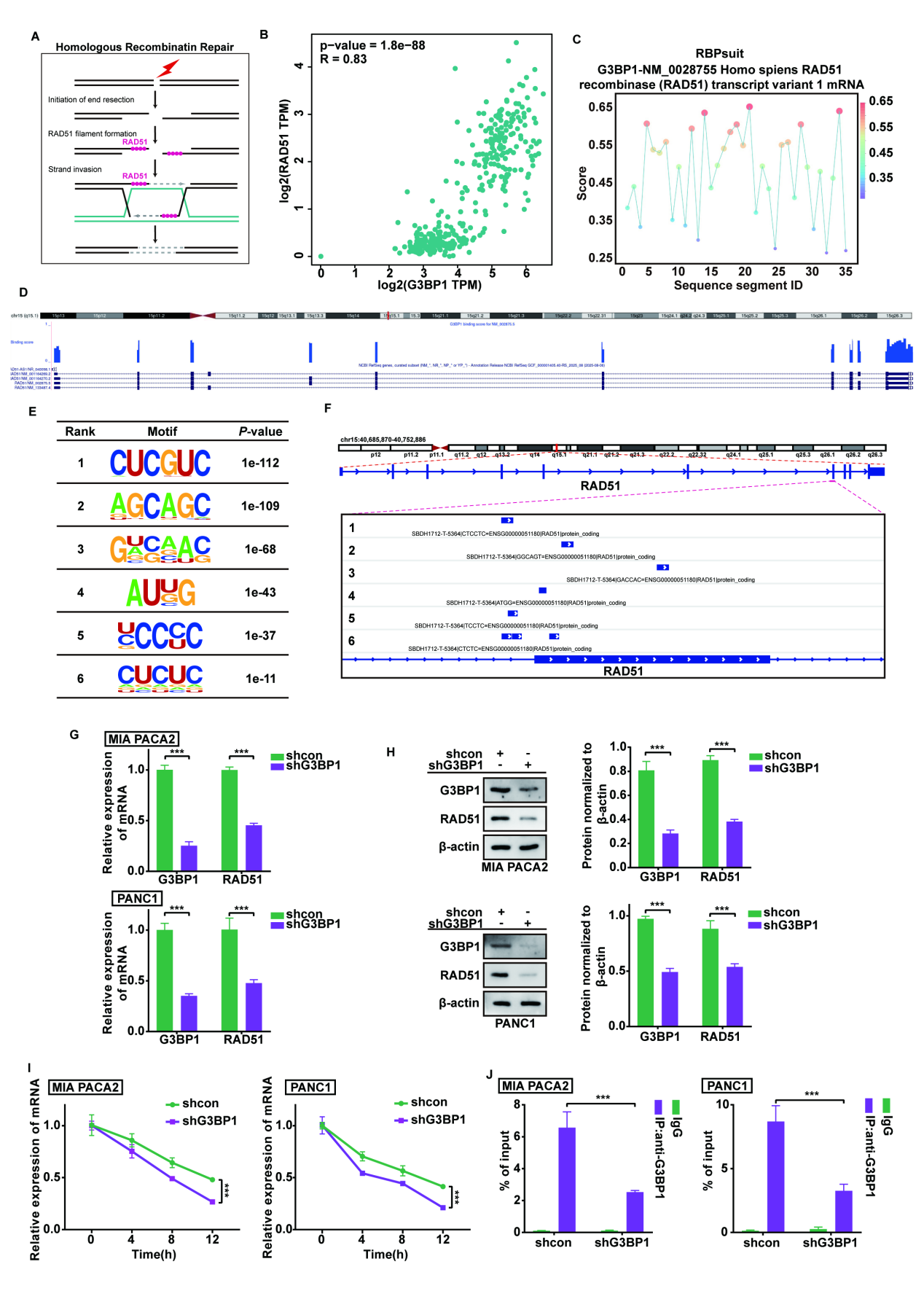


**Supplemental Fig.8** **A** Schematic diagram of key pathways for homologous recombination repair. **B** Correlation analysis of *RAD51* and *G3BP1* expression in pancreatic cancer based on GEPIA database. **C,D** The RBPsuit website predicted the potential interaction score and visualization between G3BP1 and *RAD51*mRNA. **E** The six G3BP1 binding motifs predicted by Starbase website show significant differences. **F** Visualize the interaction sites on the *RAD51* gene corresponding to the binding motif. **G** Analyze the mRNA levels of *G3BP1* and *RAD51* in MIA PaCa-2 and PANC-1 cells after knocking down *G3BP1* using qRT PCR (mean ± SD, ***P < 0.001, unpaired Student’s t test, n=3). **H** Analyze the protein levels of G3BP1 and RAD51 in MIA PaCa-2 and PANC-1 cells after knocking down *G3BP1* using western blot (mean ± SD, *P < 0.05, ***P < 0.001, Mann-Whitney U test, n = 3). **I** *RAD51* mRNA decay rates upon *G3BP1* knockdown were measured by qRT-PCR in MIA PaCa-2 and PANC-1 cells after transcriptional arrest with Actinomycin D (5µg/mL). (mean ± SD, ***P < 0.001, vs. control group, two-way repeated-measures ANOVA, n = 6) **J** G3BP1 binding to *RAD51* mRNA assessed by RIP-qPCR in control and *G3BP1*-knockdown MIA PaCa-2 and PANC-1 cells (mean ± SD, ***P < 0.001, Mann-Whitney U test, n = 3).
