# Supplementary material for: NUP93 facilitates the nuclear import of SOX2 to activate G3BP1 transcription and impairs gemcitabine response in pancreatic cancer
Source: Cell Death Dis. 2026 Mar 28;17(1):423. doi: 10.1038/s41419-026-08586-4 (PMC13149976; doi:10.1038/s41419-026-08586-4)
Supplement: Supplementary file 2 — Original Western blots [file 41419_2026_8586_MOESM2_ESM.docx]

**Fig.1 H**

NUP93


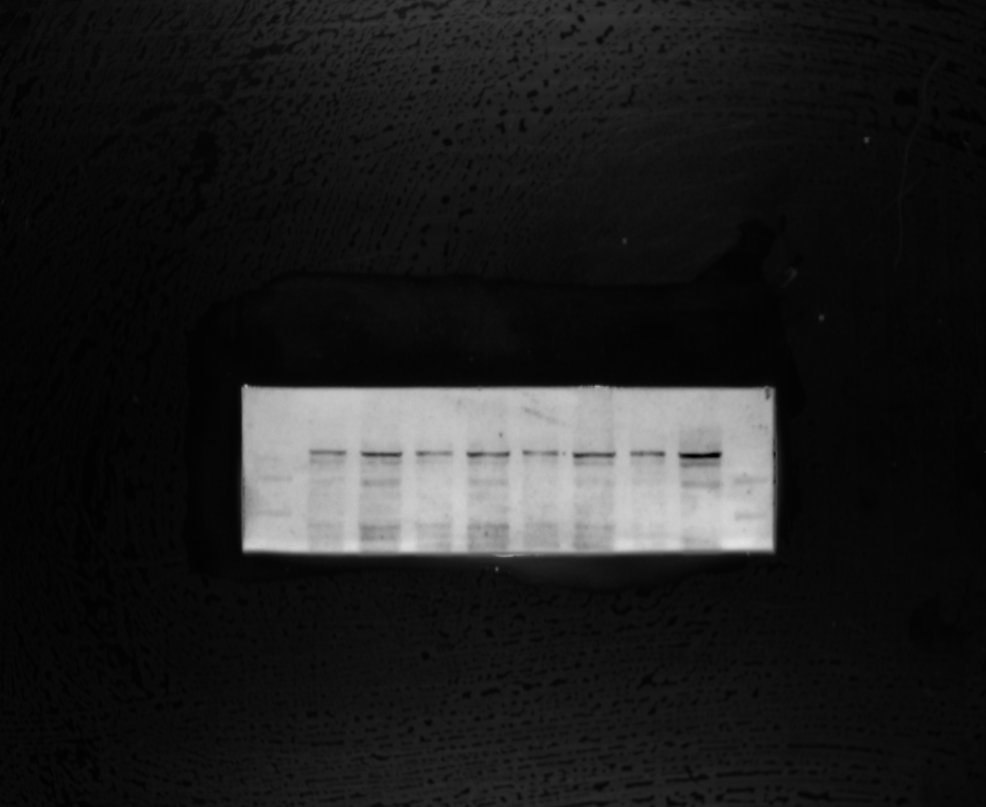


**KDa**

70

100

β-Actin


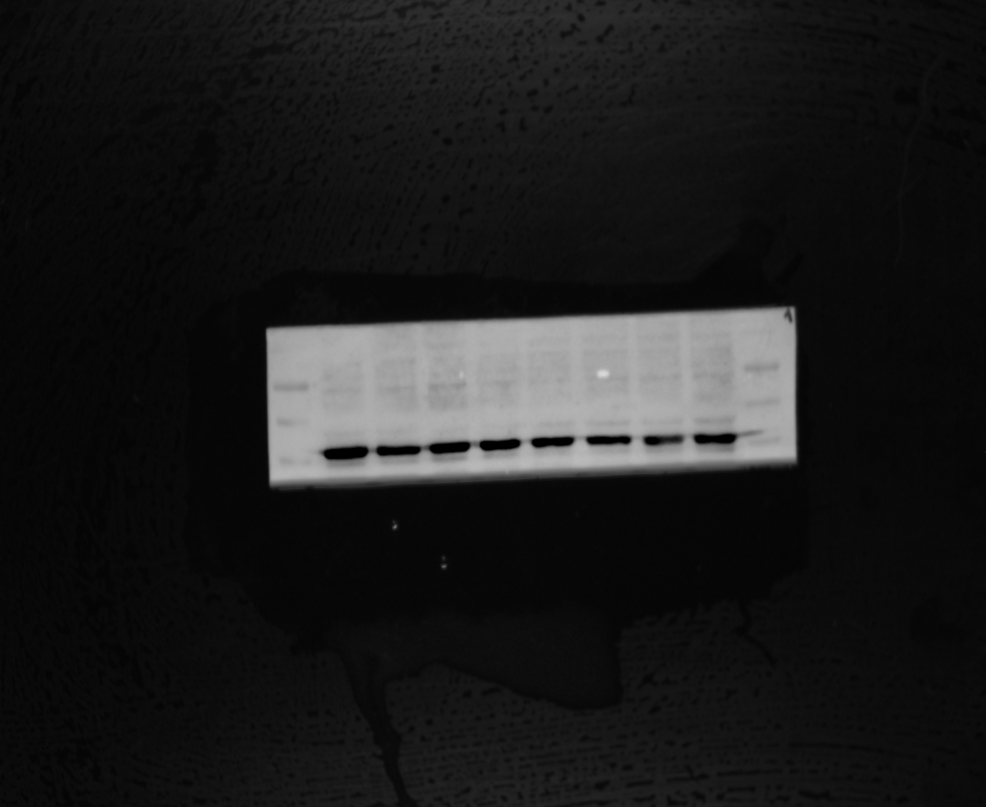


**KDa**

55

43

NUP93


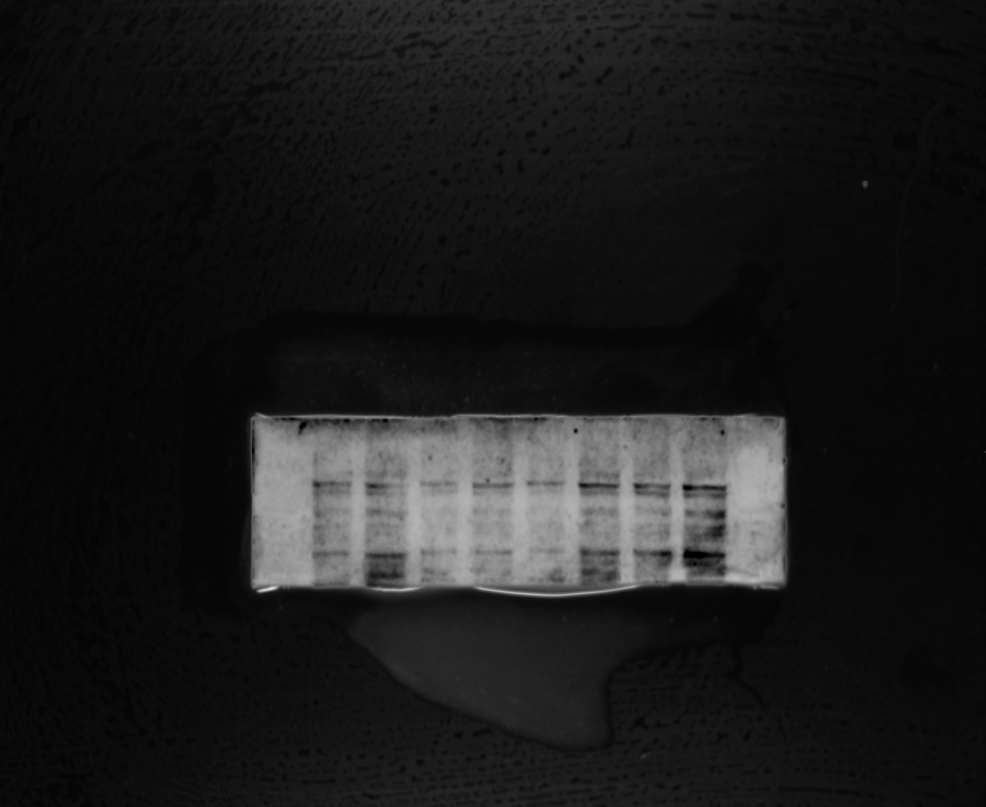


**KDa**

100

70

β-Actin


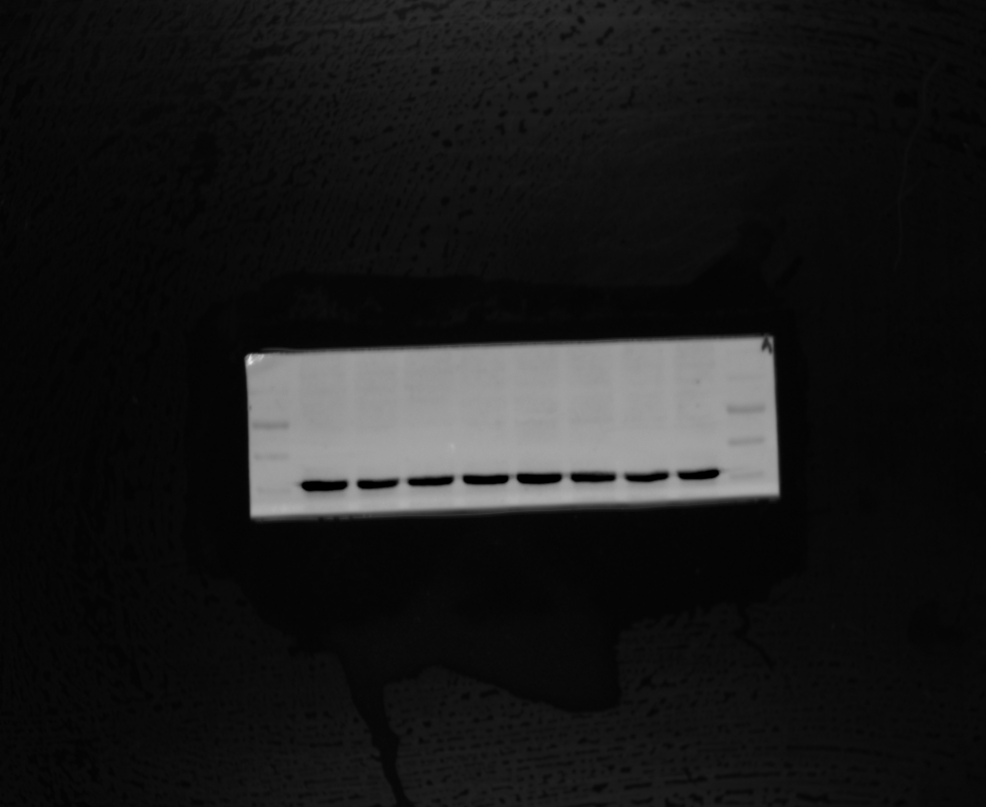


**KDa**

55

43

**Fig.1 K**

NUP93


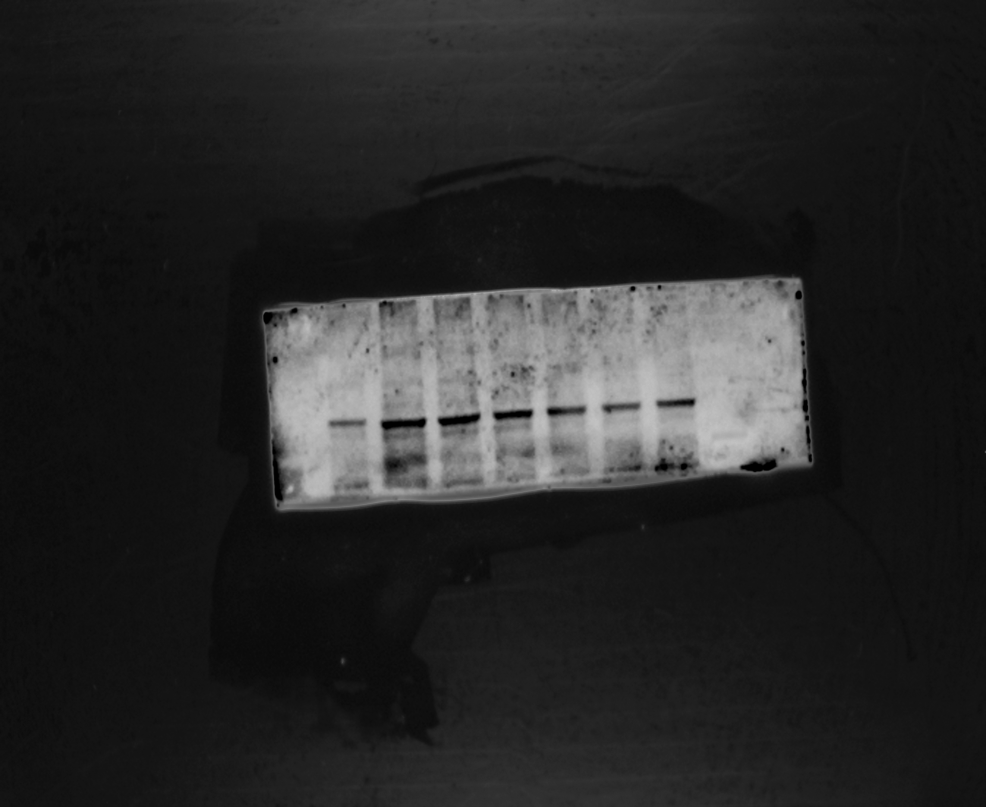


**KDa**

100

70

β-Actin


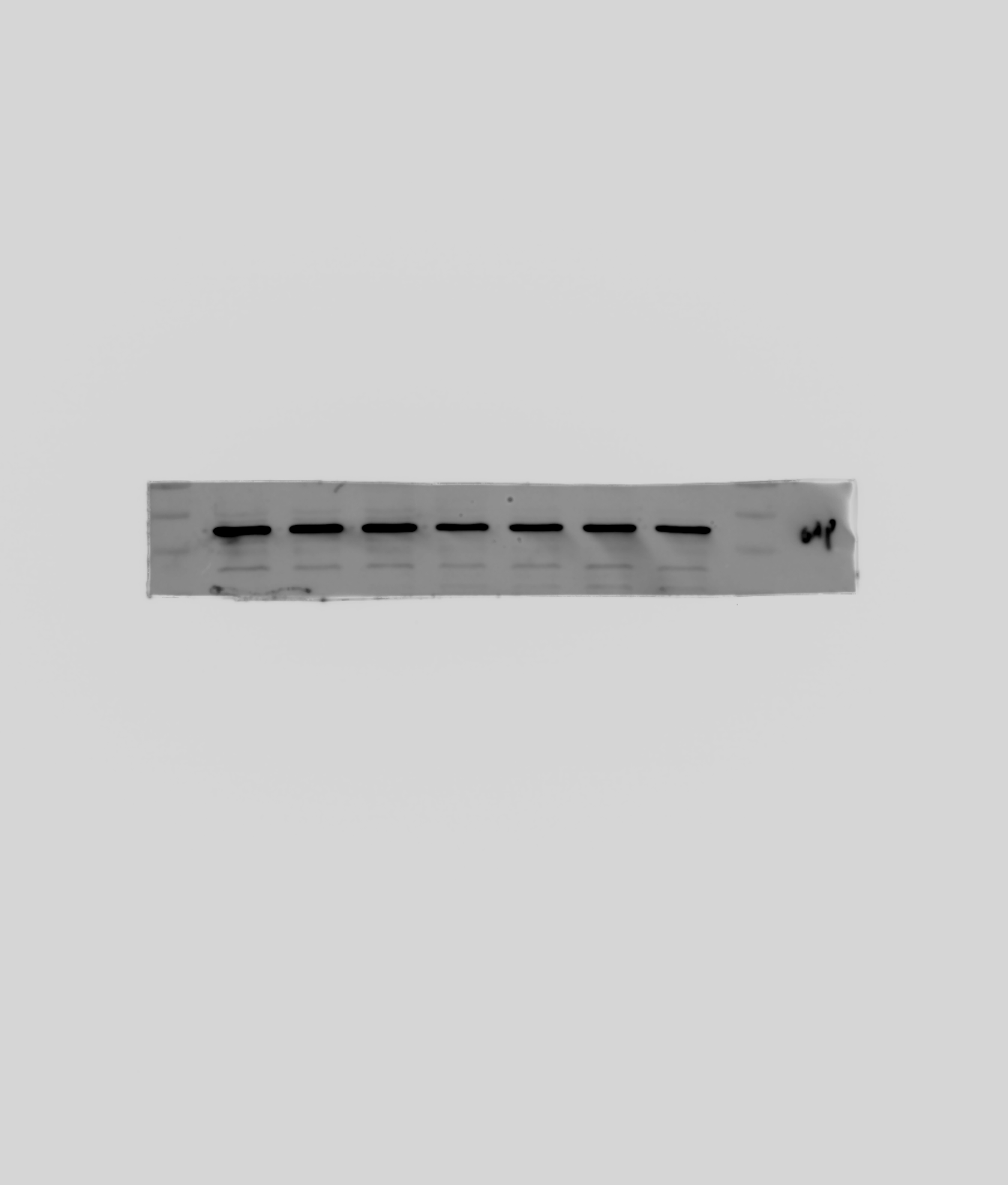


**KDa**

55

43

**Fig.2 A**

NUP93


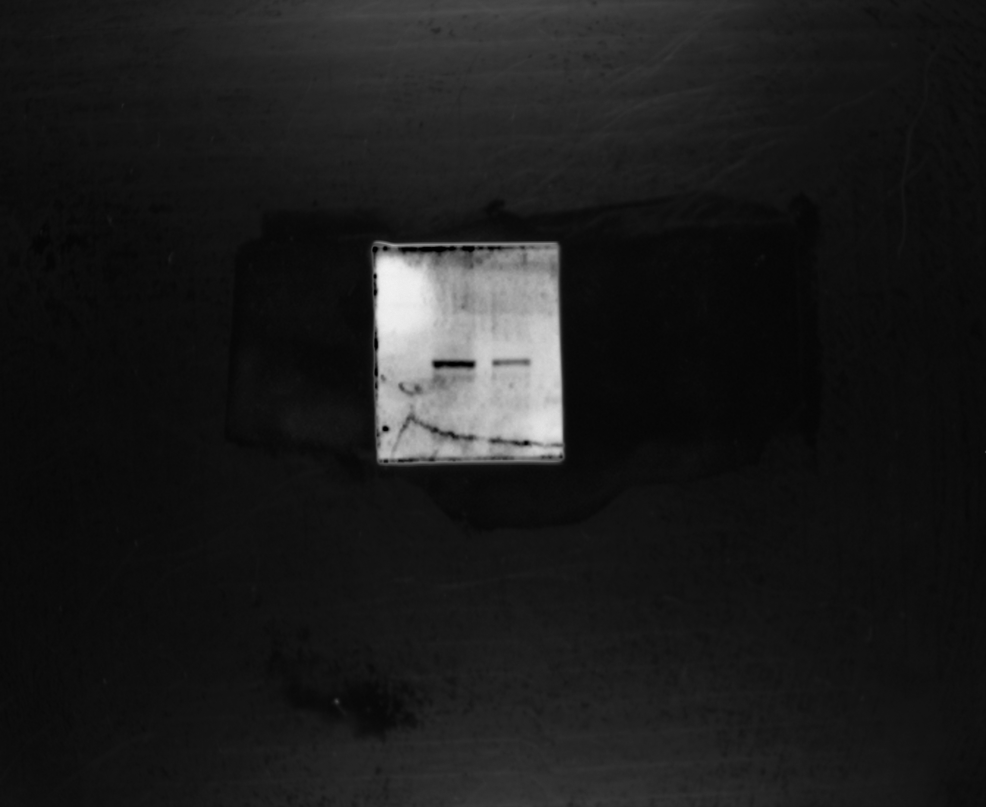


**KDa**

100

70

β-Actin


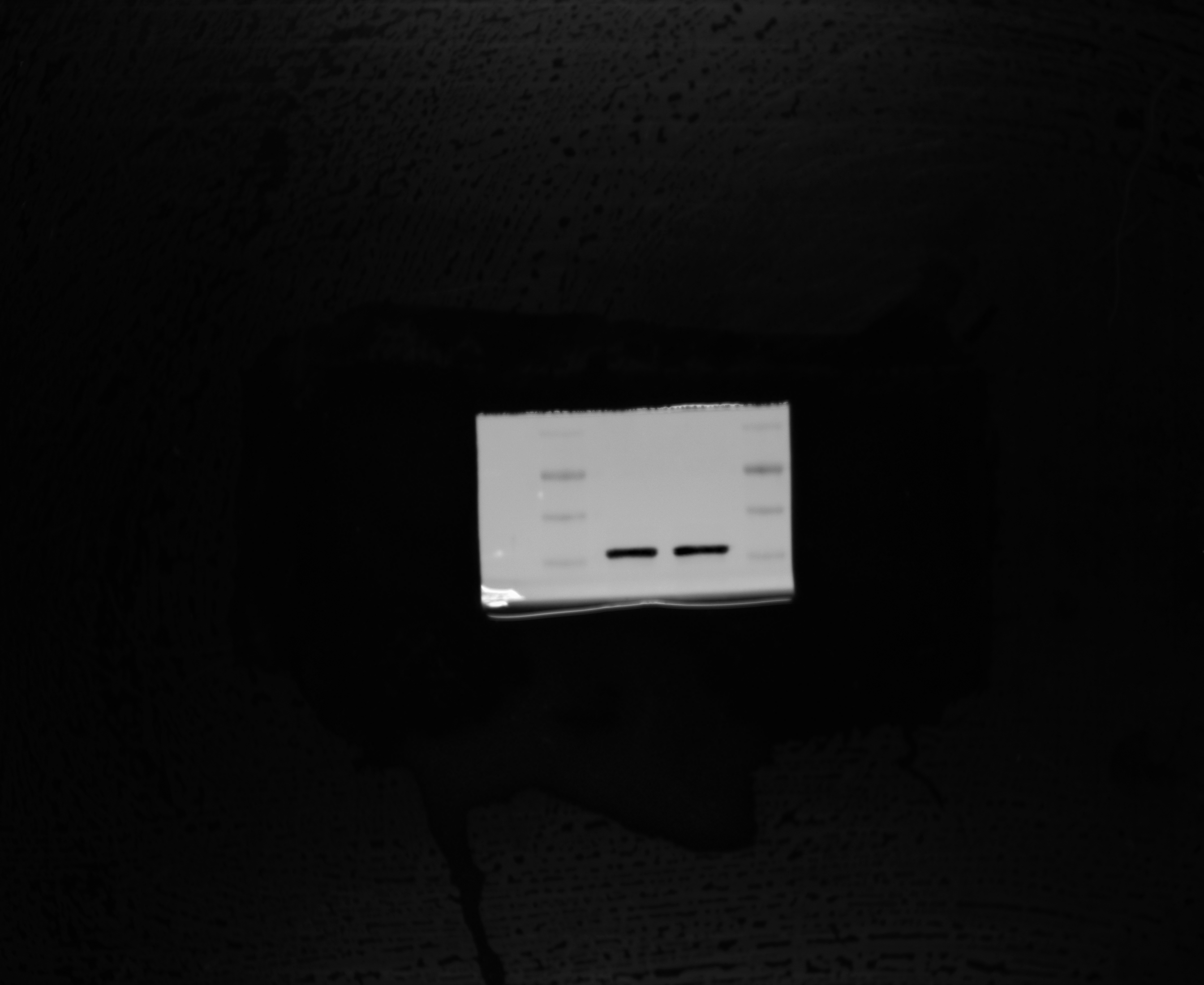


**KDa**

55

43

NUP93


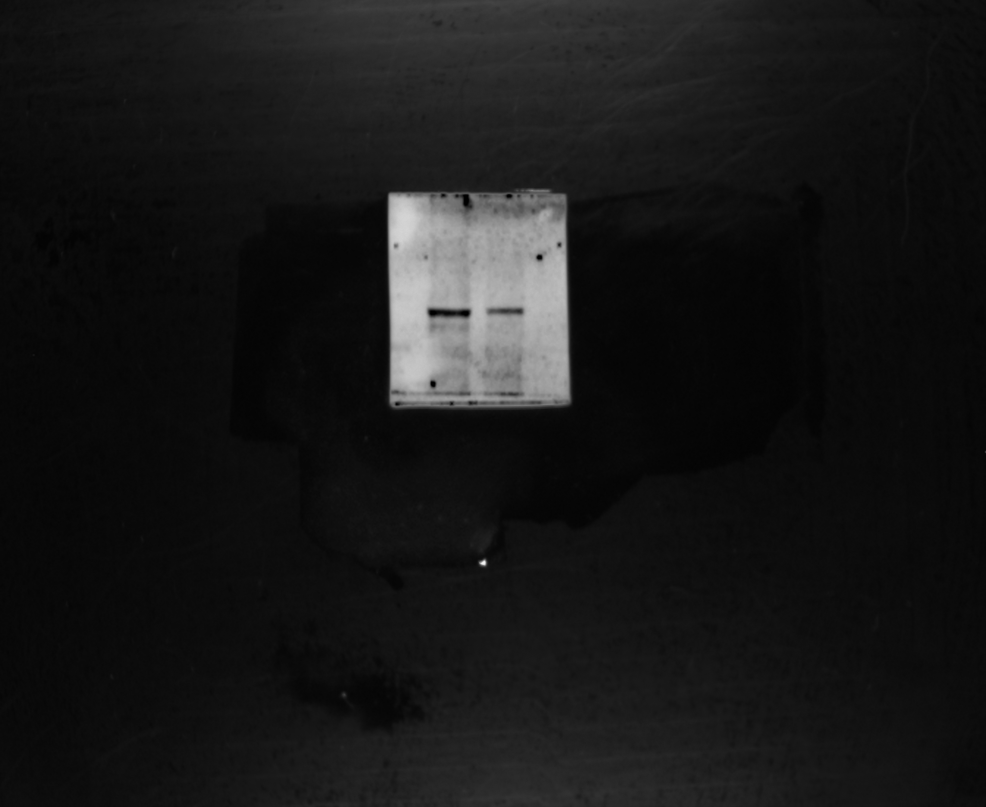


**KDa**

100

70

β-Actin


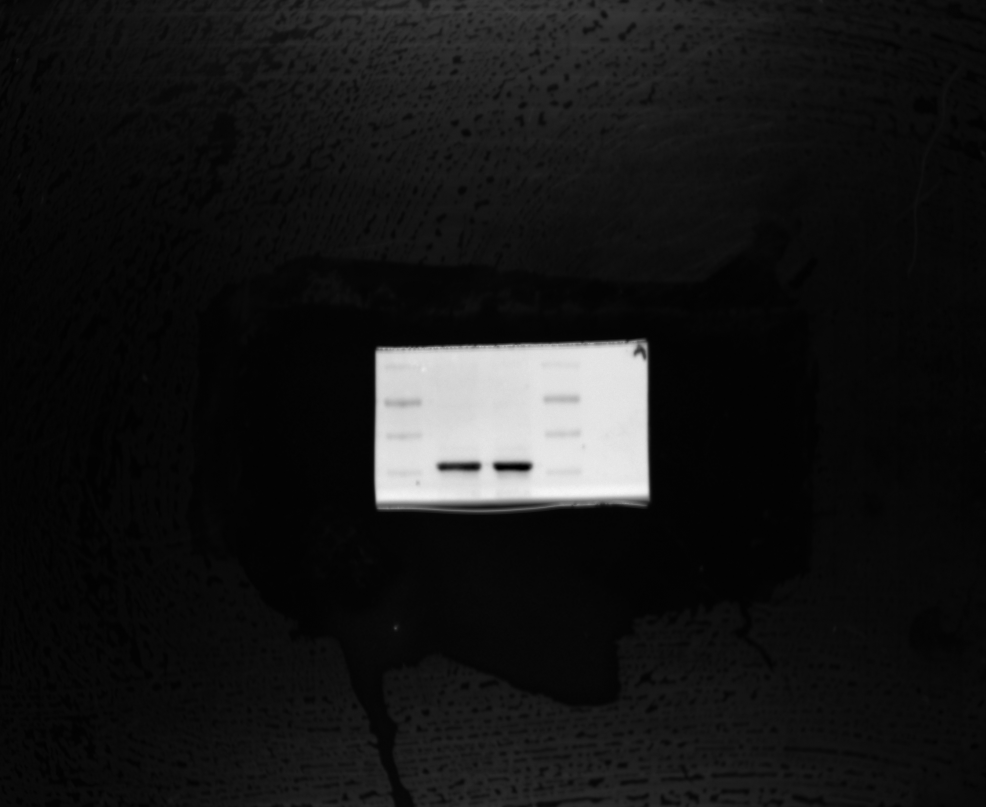


**KDa**

55

43

**Fig.2 B**

NUP93


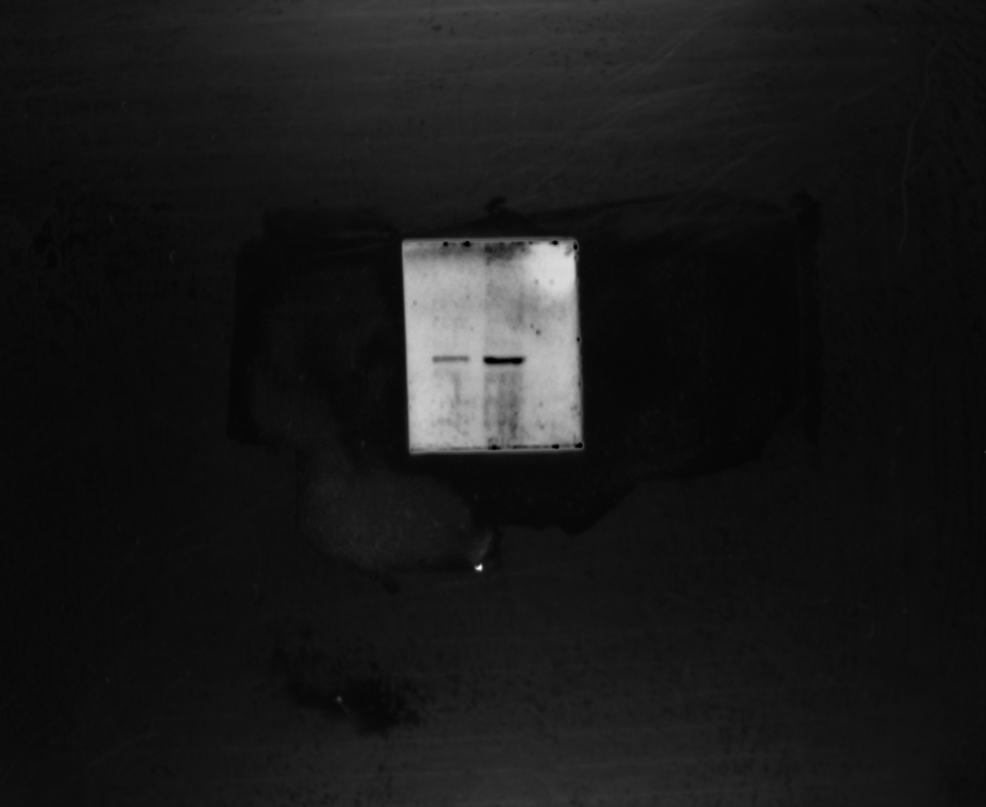


**KDa**

100

70

β-Actin


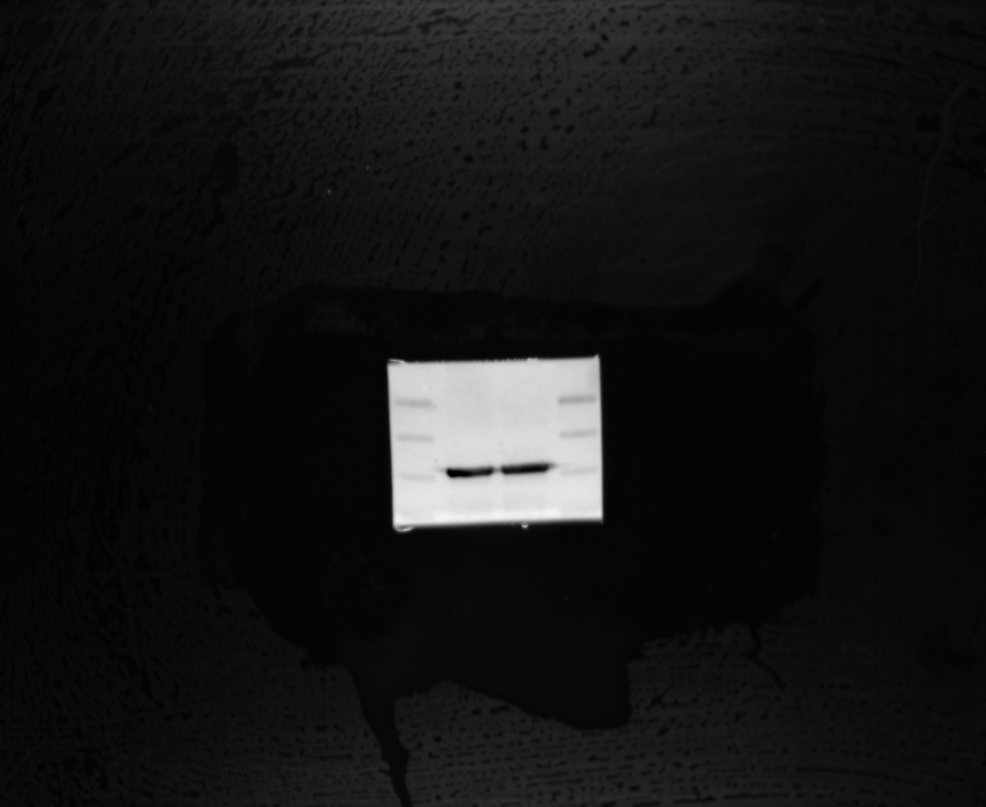


**KDa**

55

43

NUP93


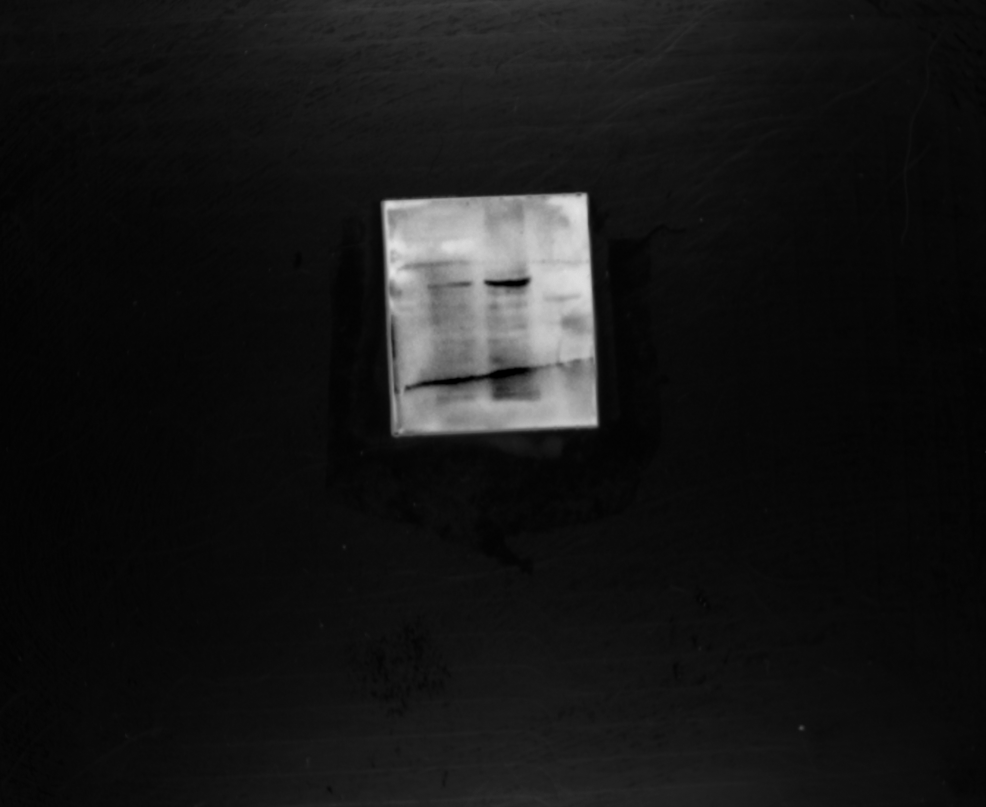


**KDa**

100

70

β-Actin


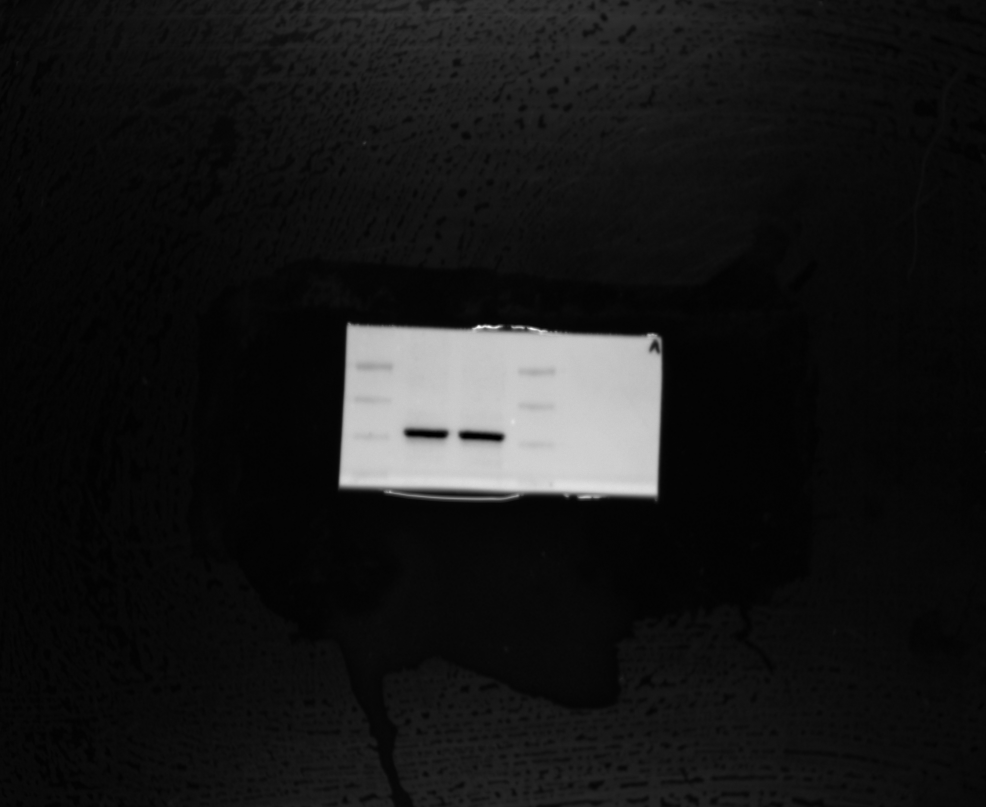


**KDa**

55

43

**Fig.3 F**

γH2AX


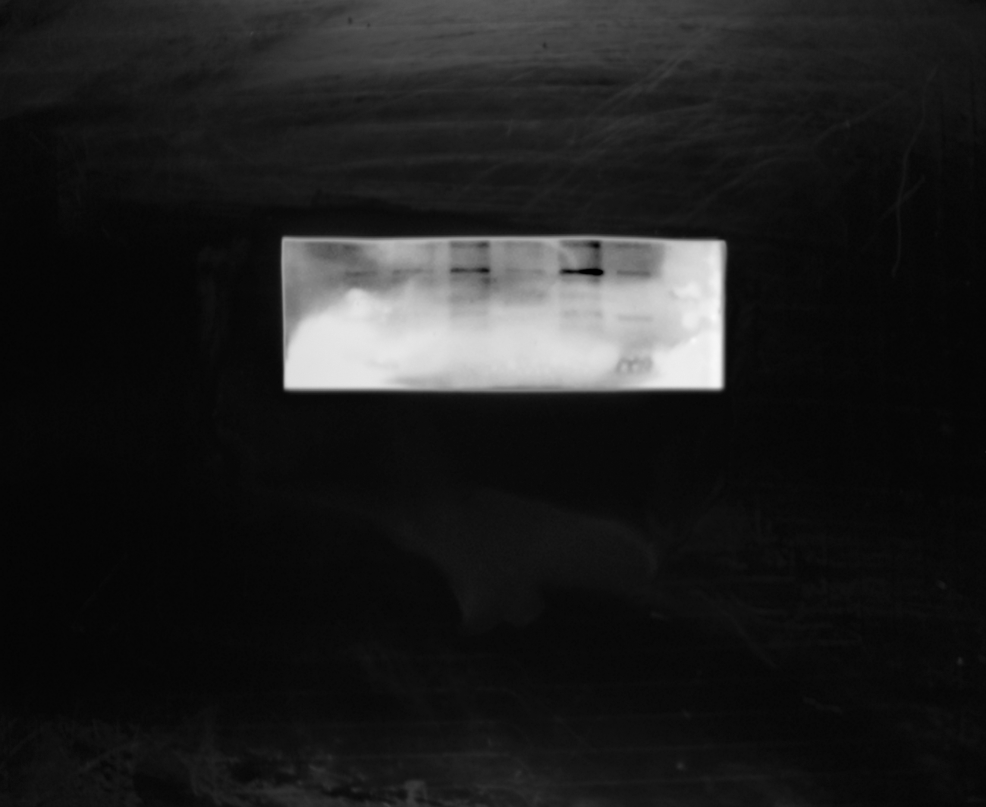


**KDa**

15

8

β-Actin


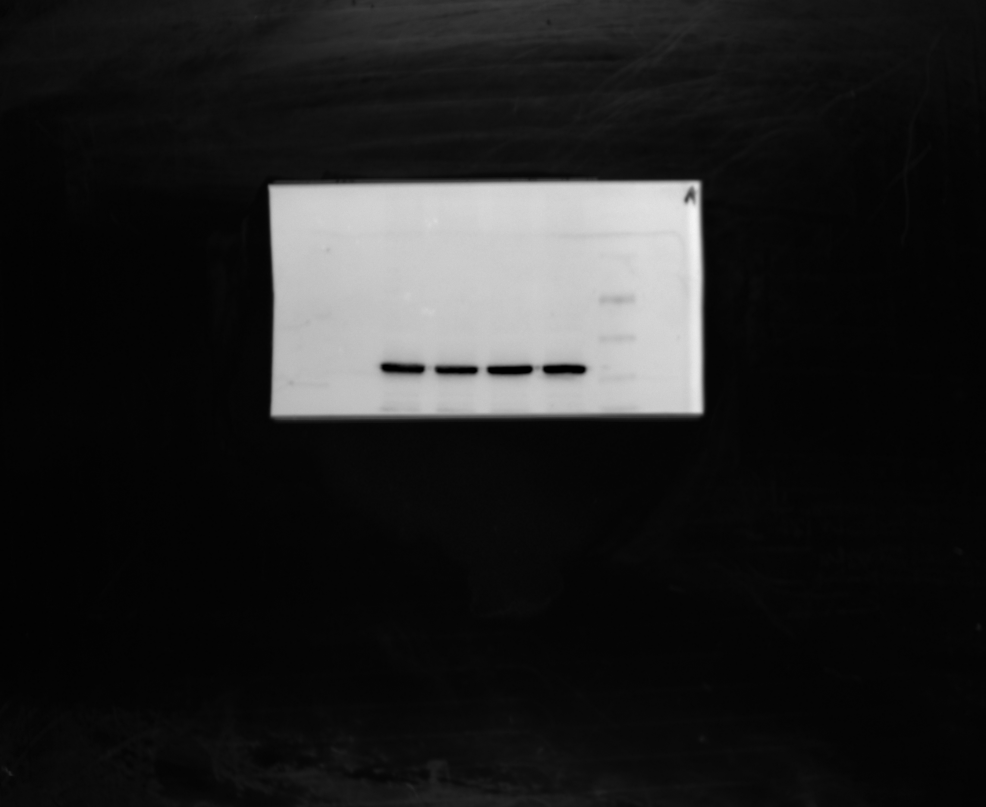


**KDa**

55

43

**Fig.3 G**

γH2AX


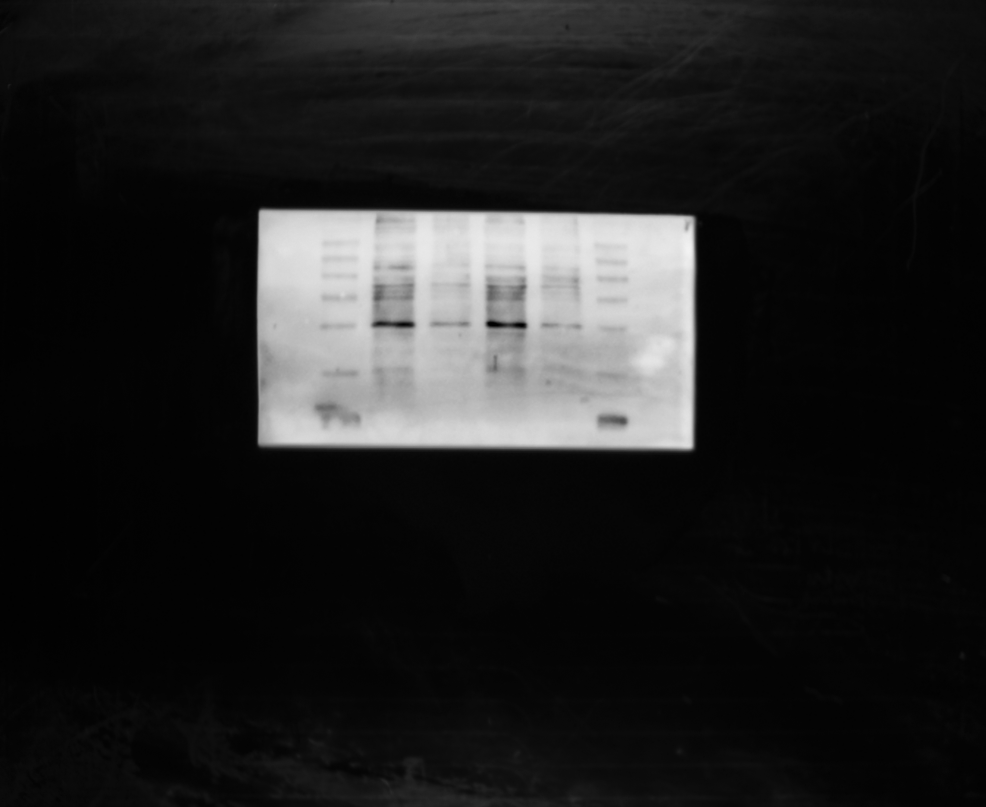


**KDa**

15

8

β-Actin


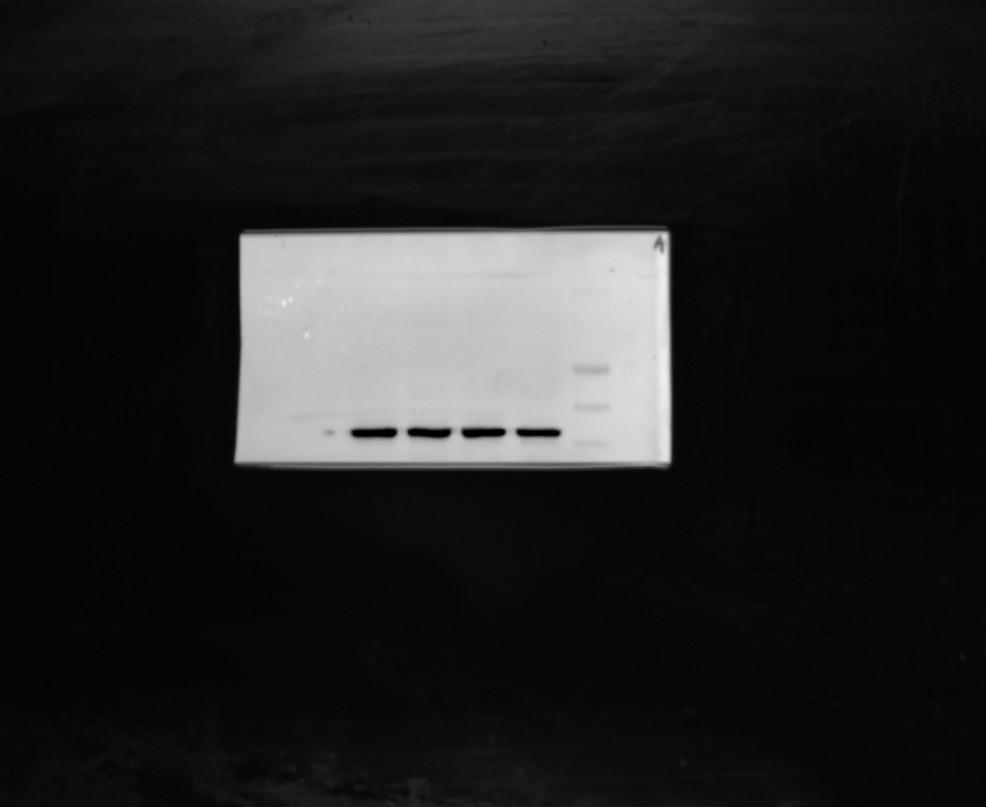


**KDa**

55

43

**Fig.4 F**

NUP93


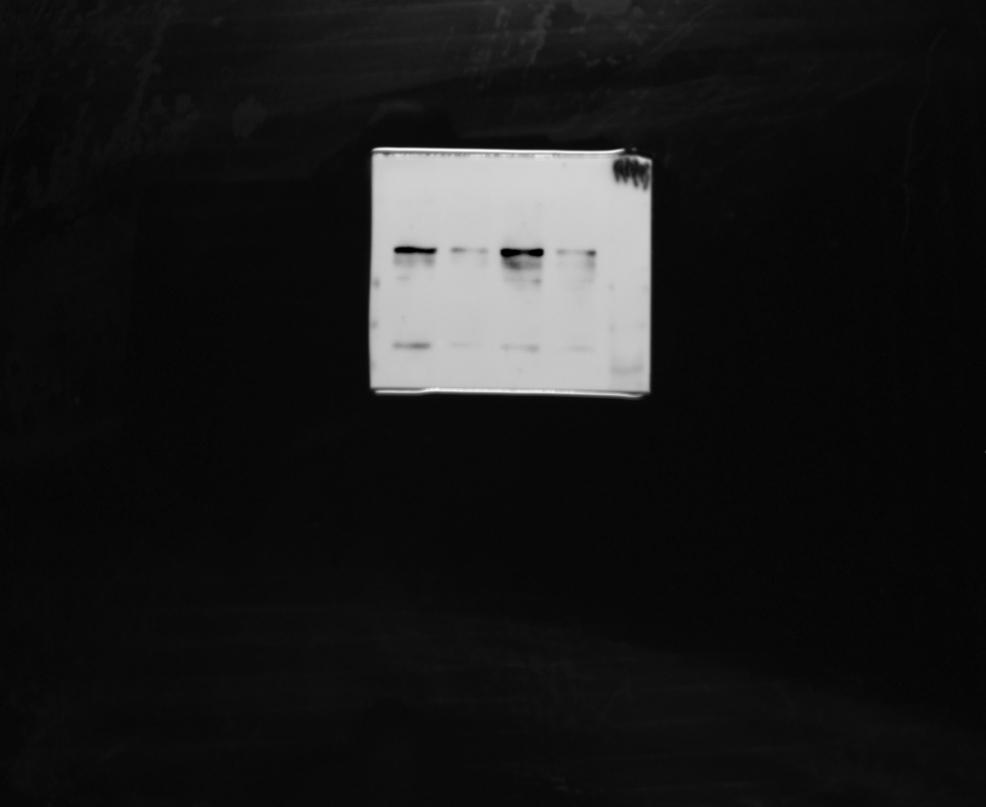


**KDa**

100

70

G3BP1


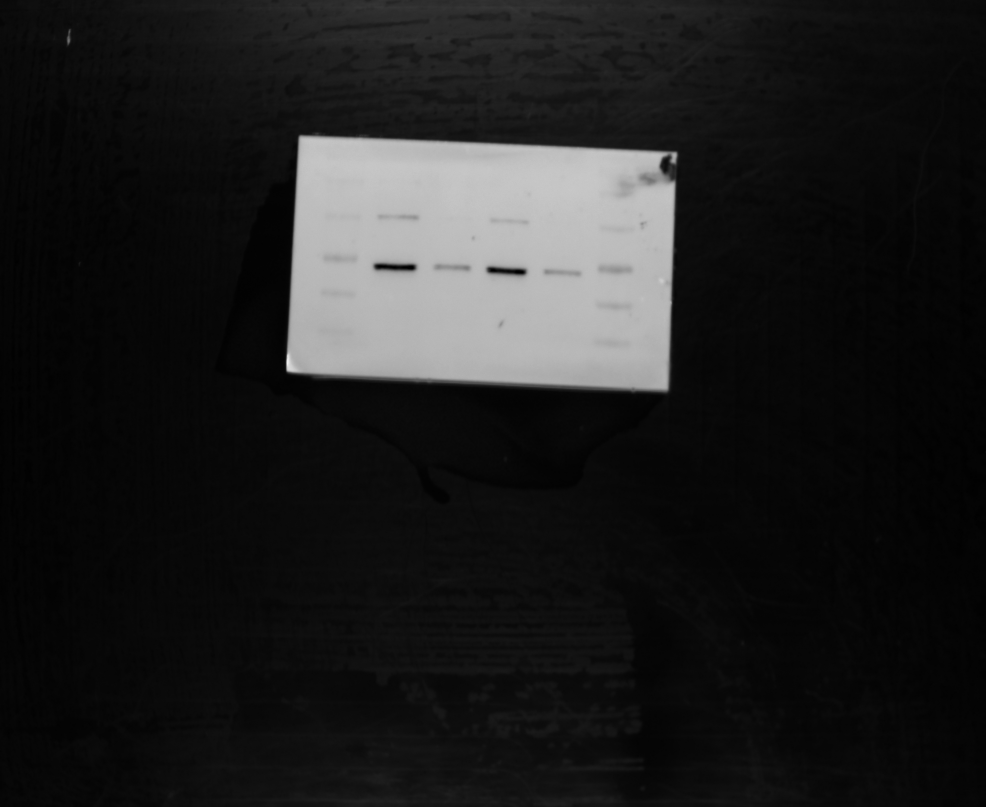


**KDa**

70

55

β-Actin


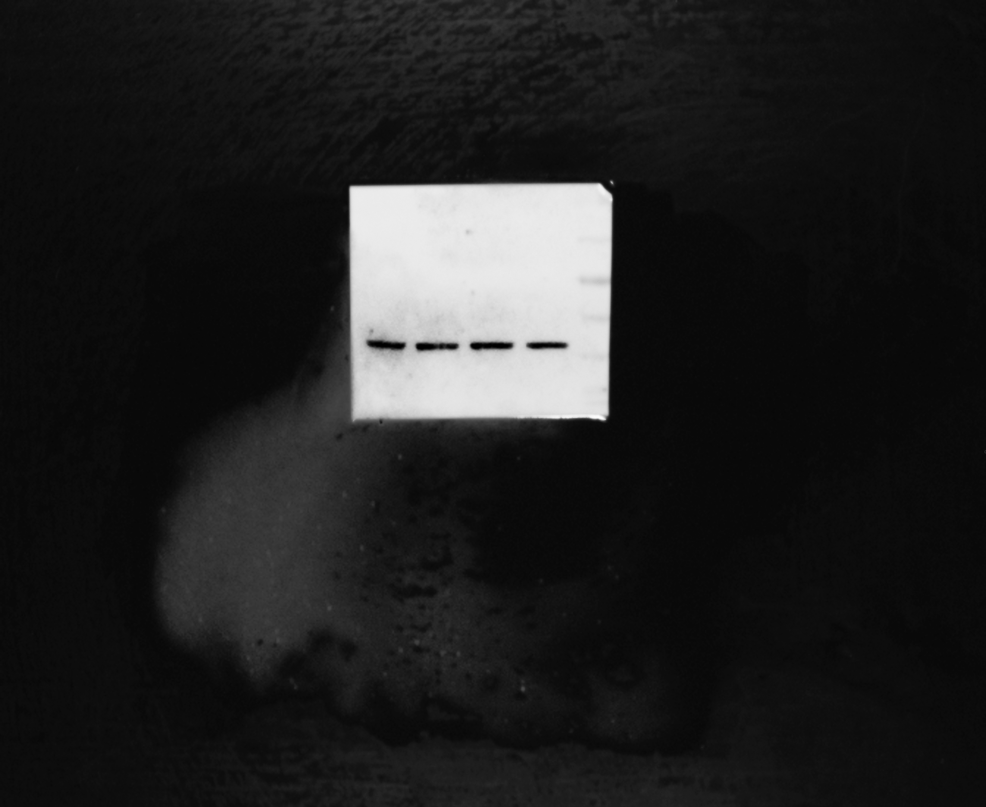


**KDa**

55

43

**Fig.4 G**

NUP93


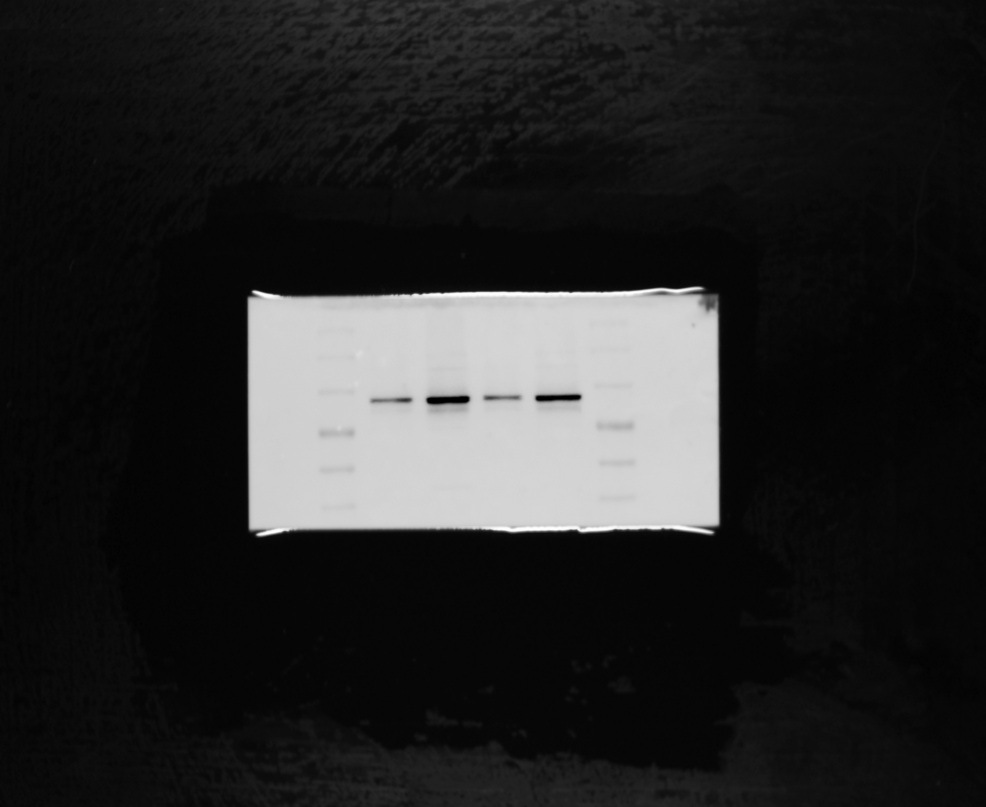


**KDa**

100

70

G3BP1


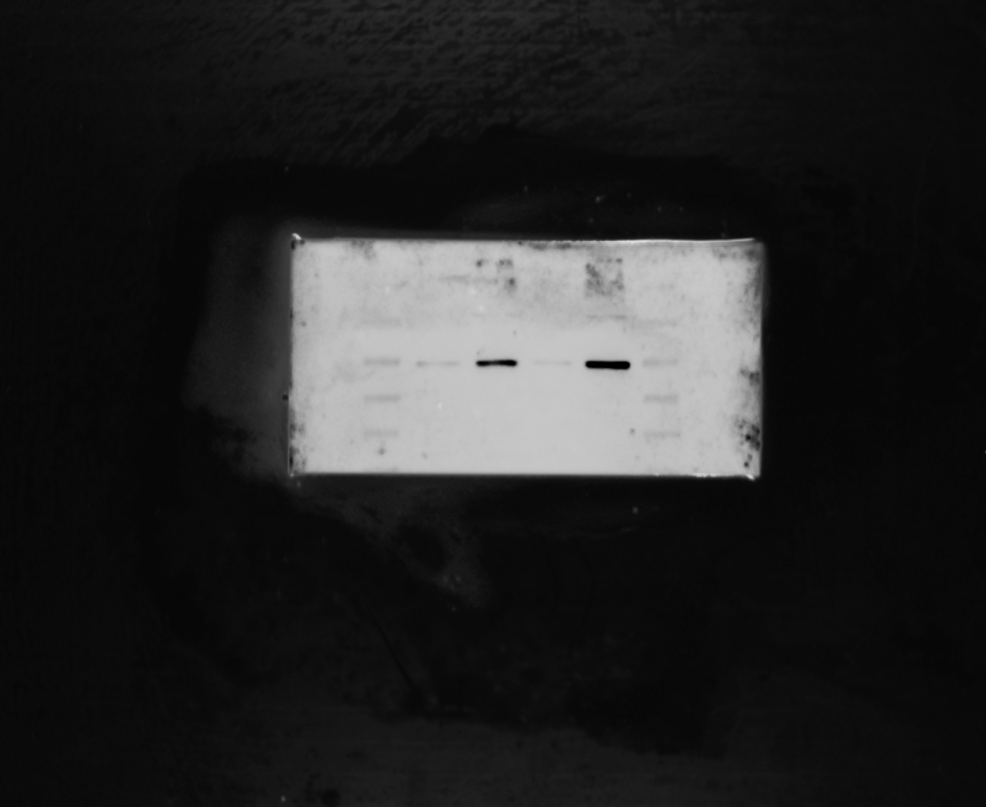


**KDa**

70

55

β-Actin


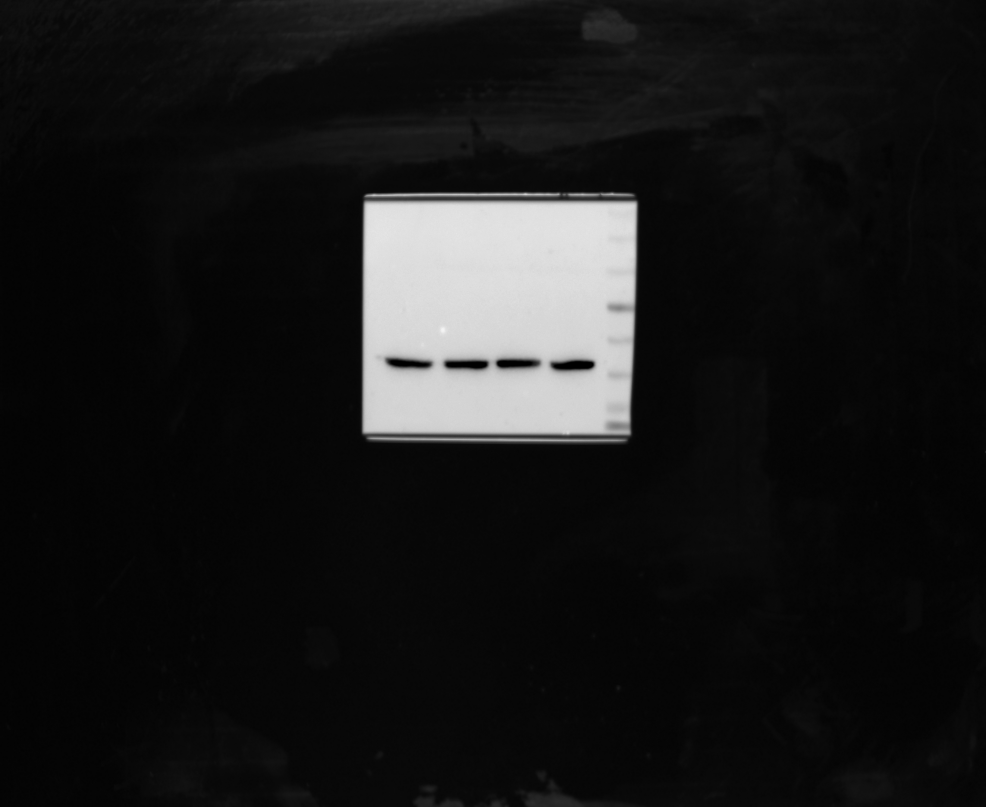


**KDa**

55

43

**Fig.4 L**

γH2AX


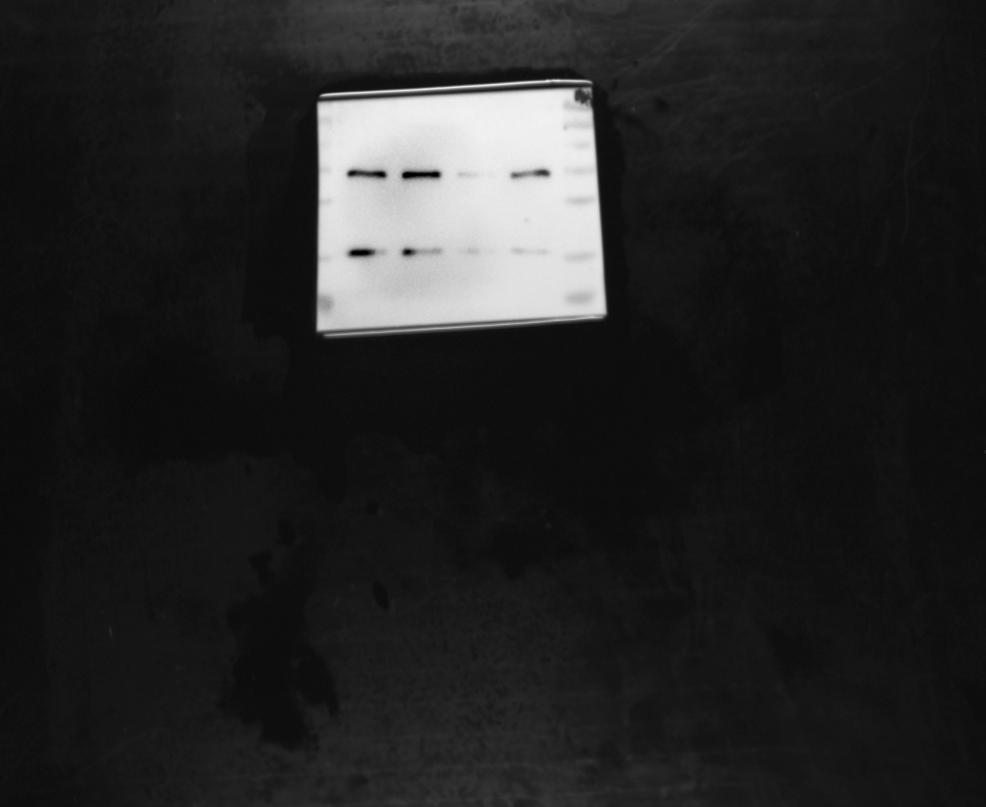


**KDa**

15

8

β-Actin


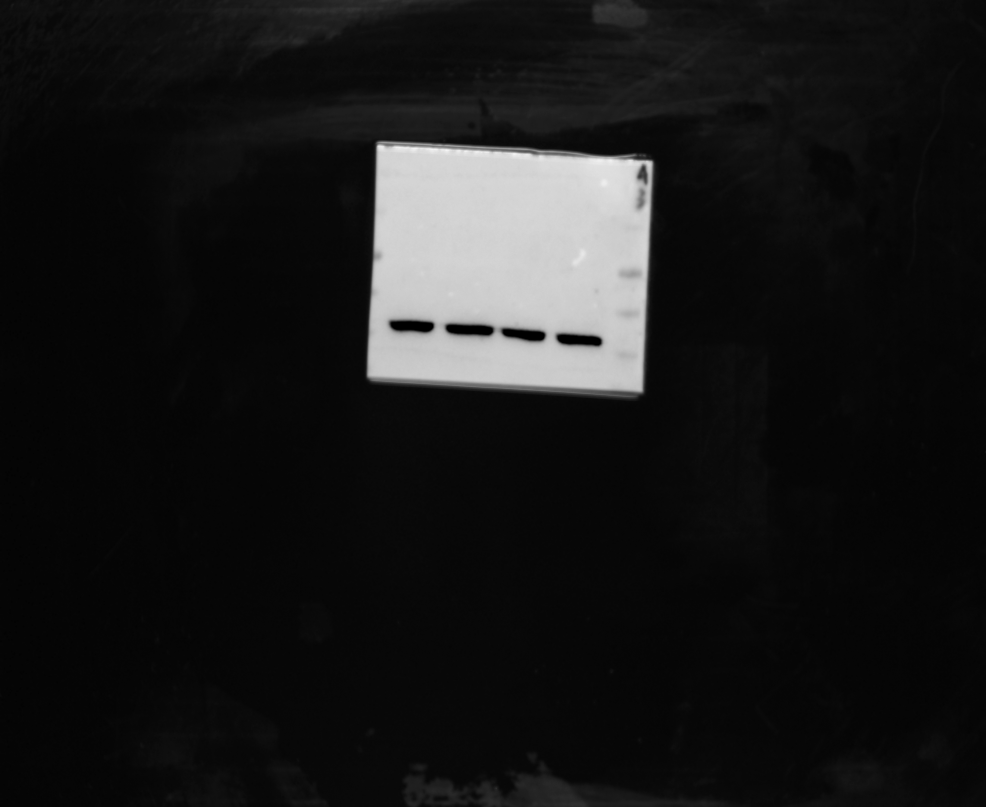


**KDa**

55

43

**Fig.4 M**

γH2AX


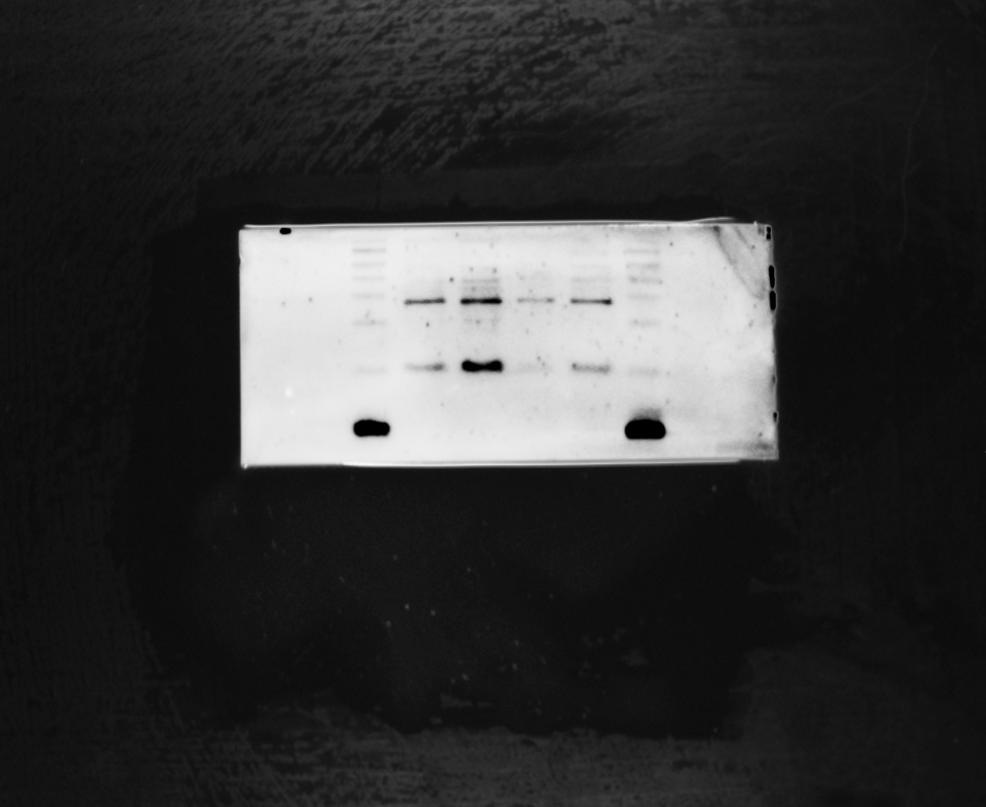


**KDa**

15

8

β-Actin


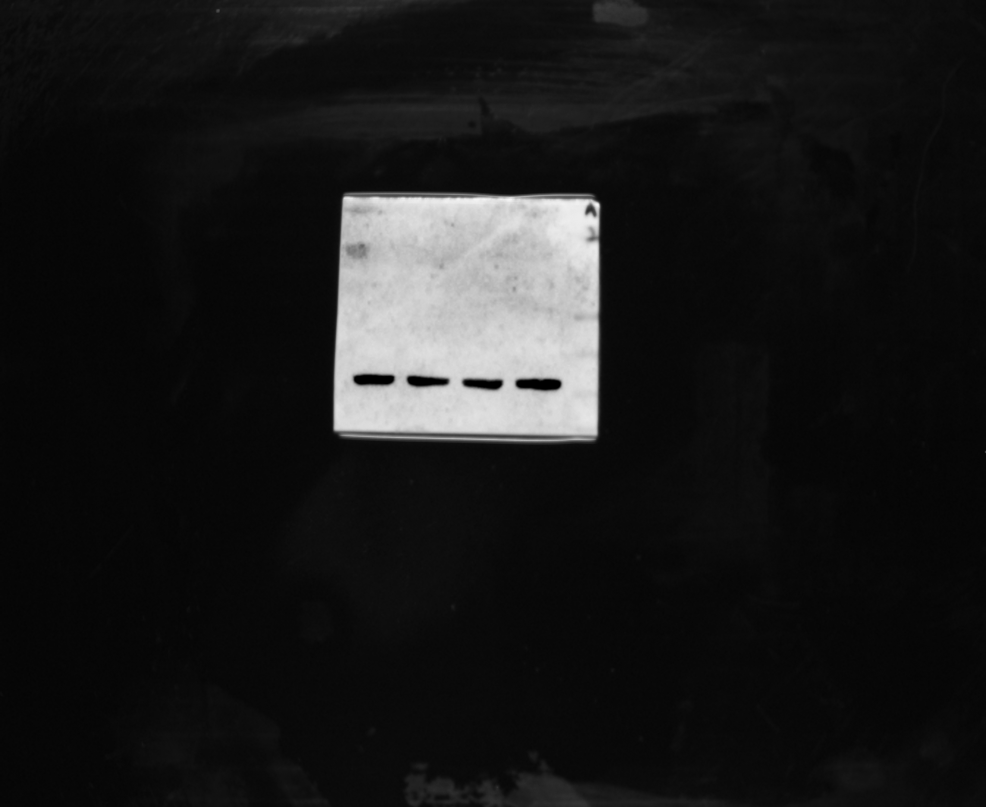


**KDa**

55

43

**Fig.5 G**

NUP93


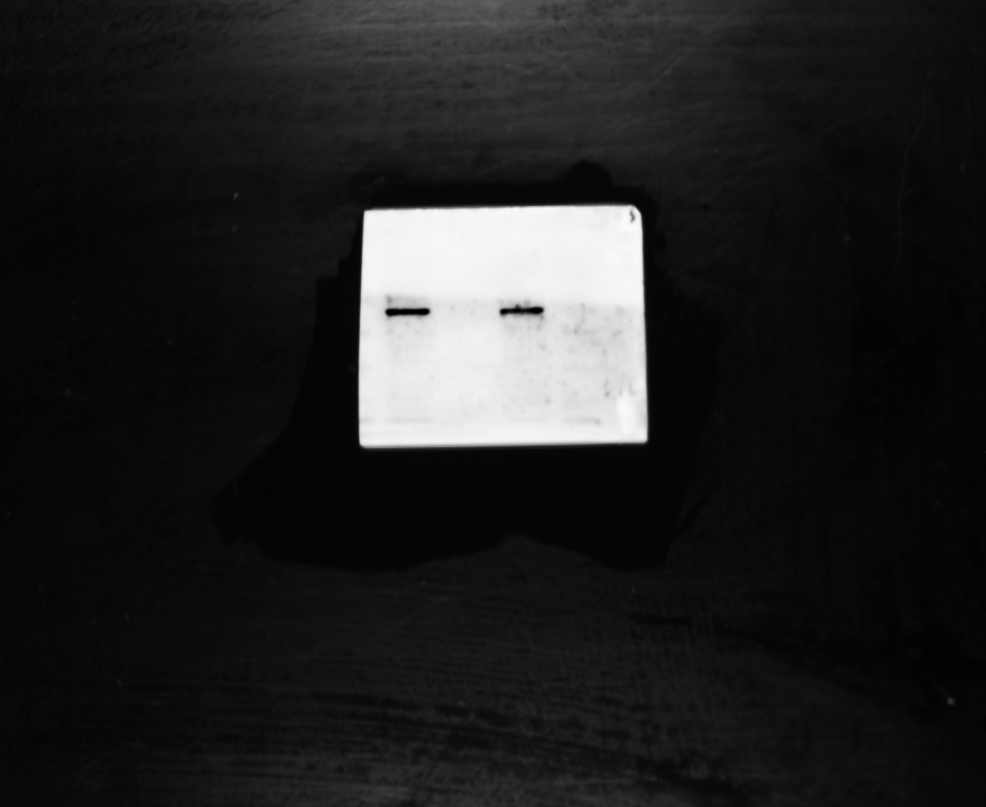


**KDa**

100

70

SOX2


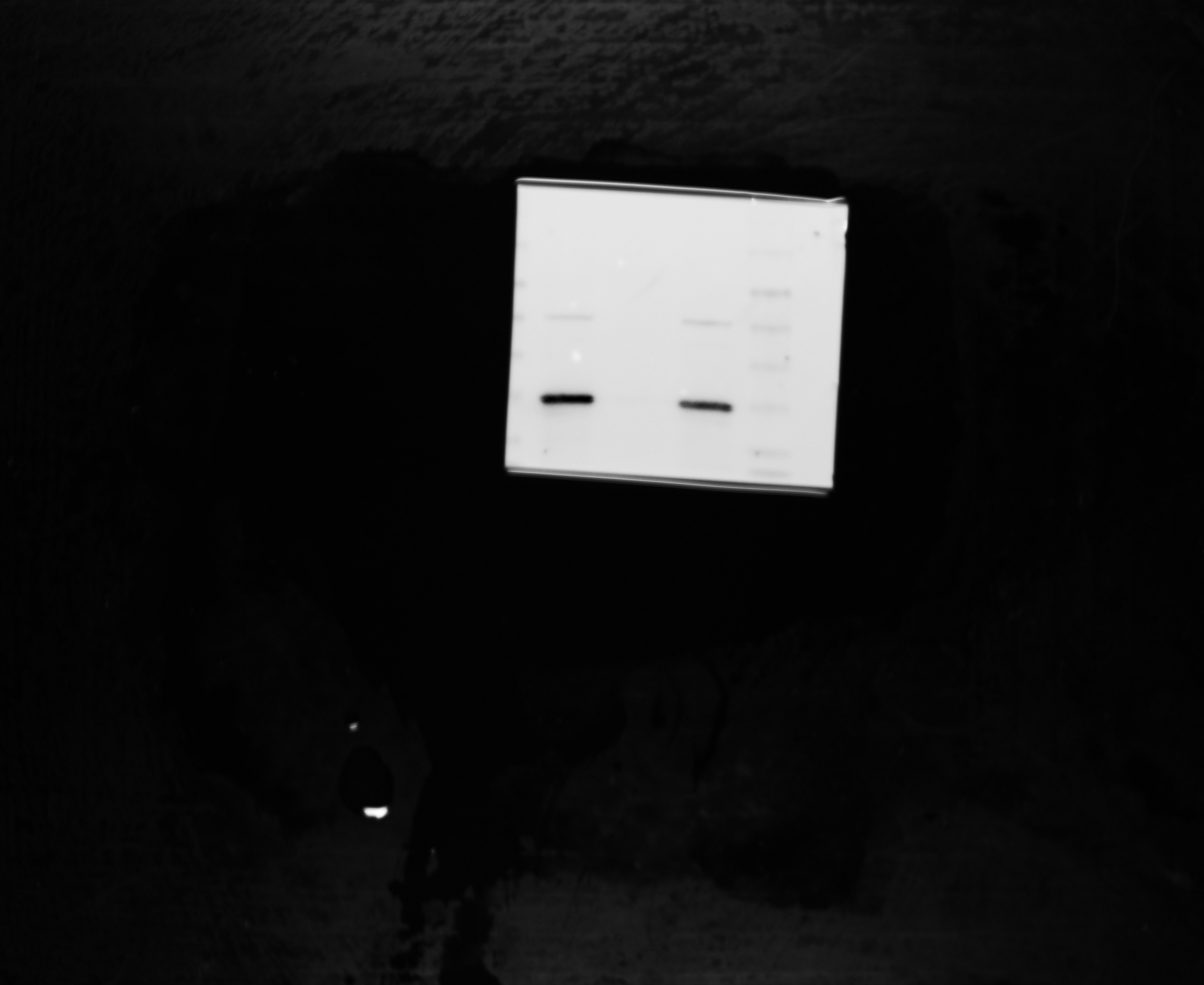


**KDa**

43

34

NUP93


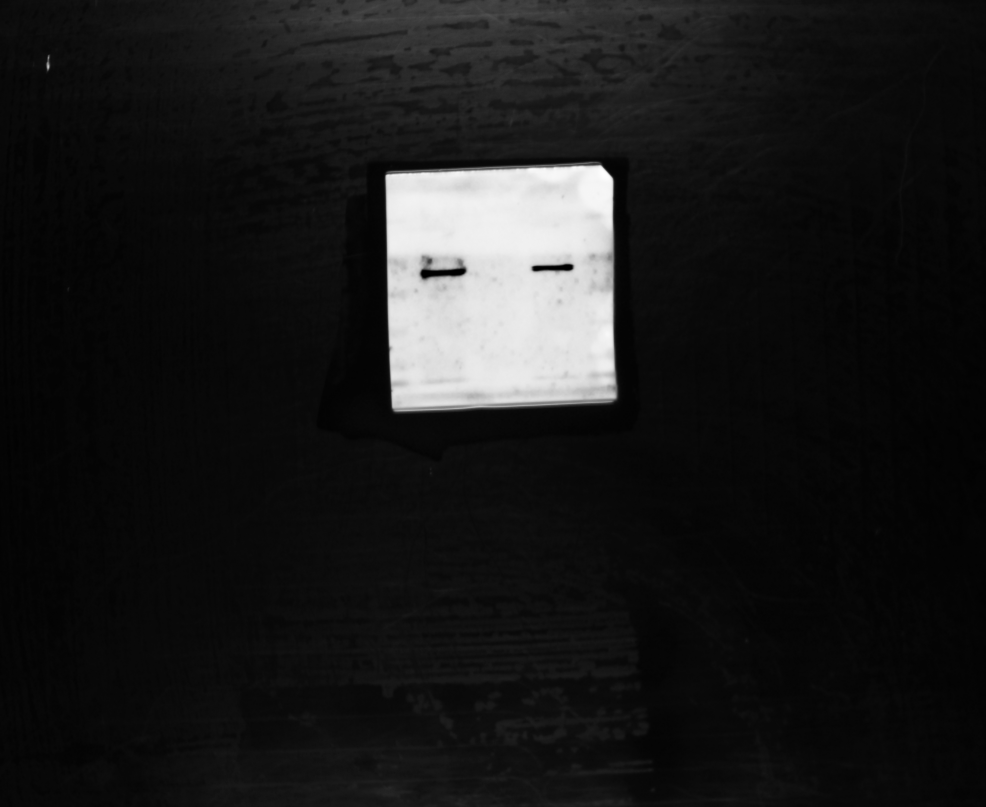


**KDa**

100

70

SOX2


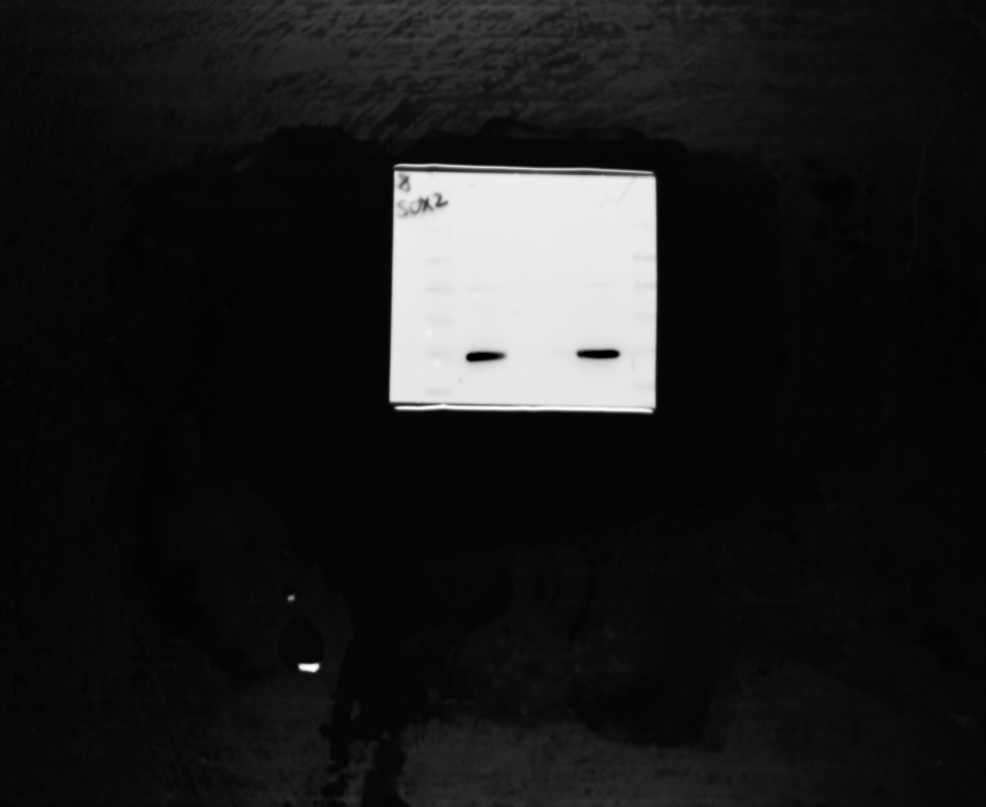


**KDa**

43

34

NUP93


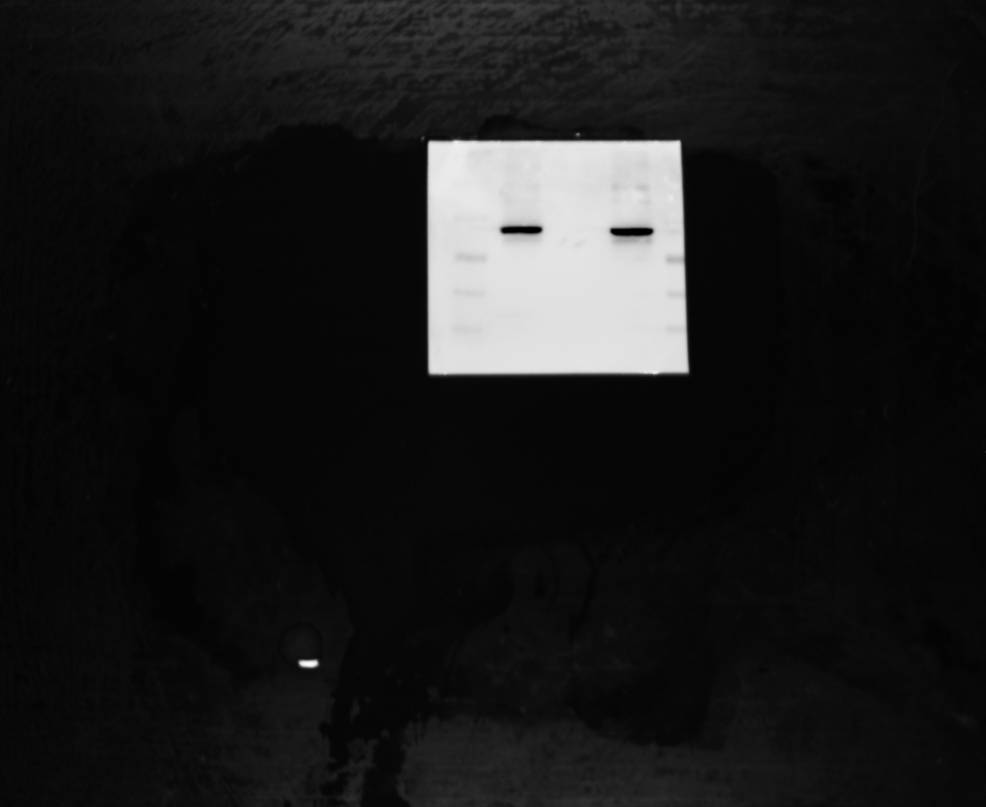


**KDa**

100

70

SOX2


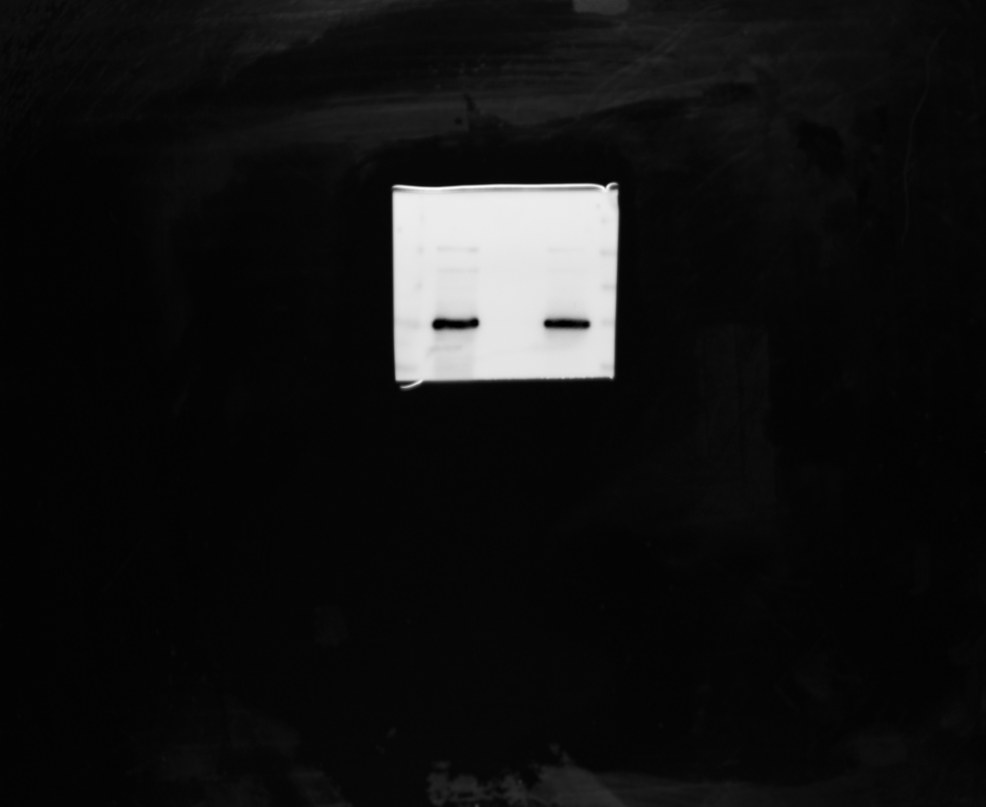


**KDa**

43

34

NUP93


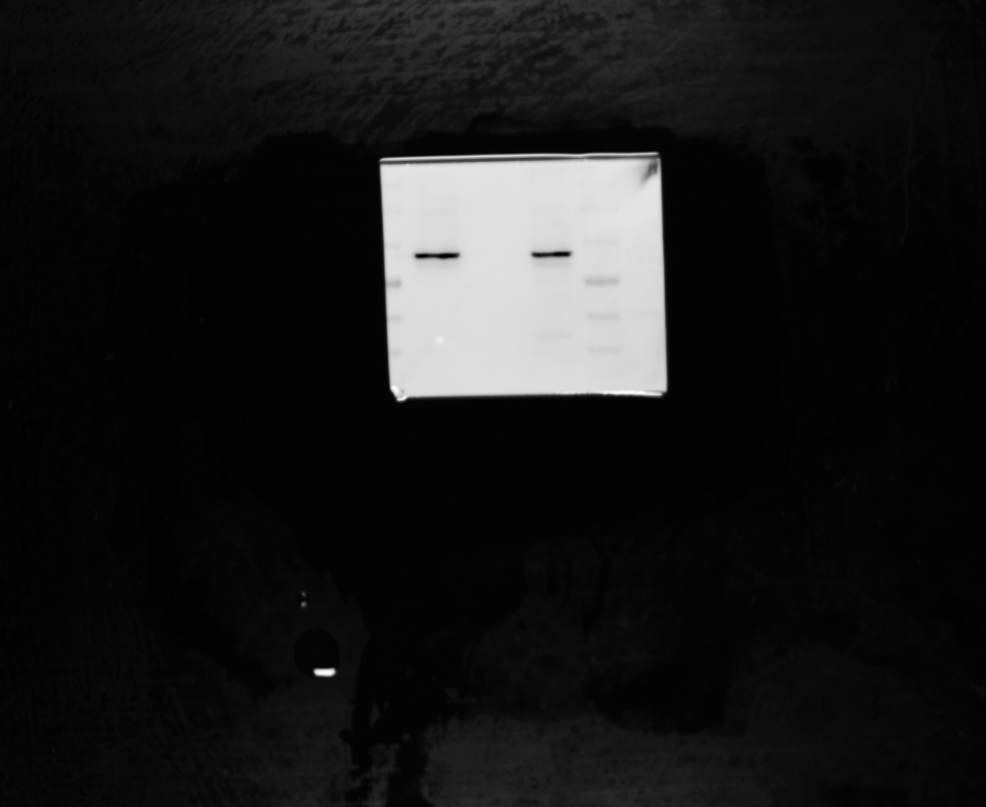


**KDa**

100

70

SOX2


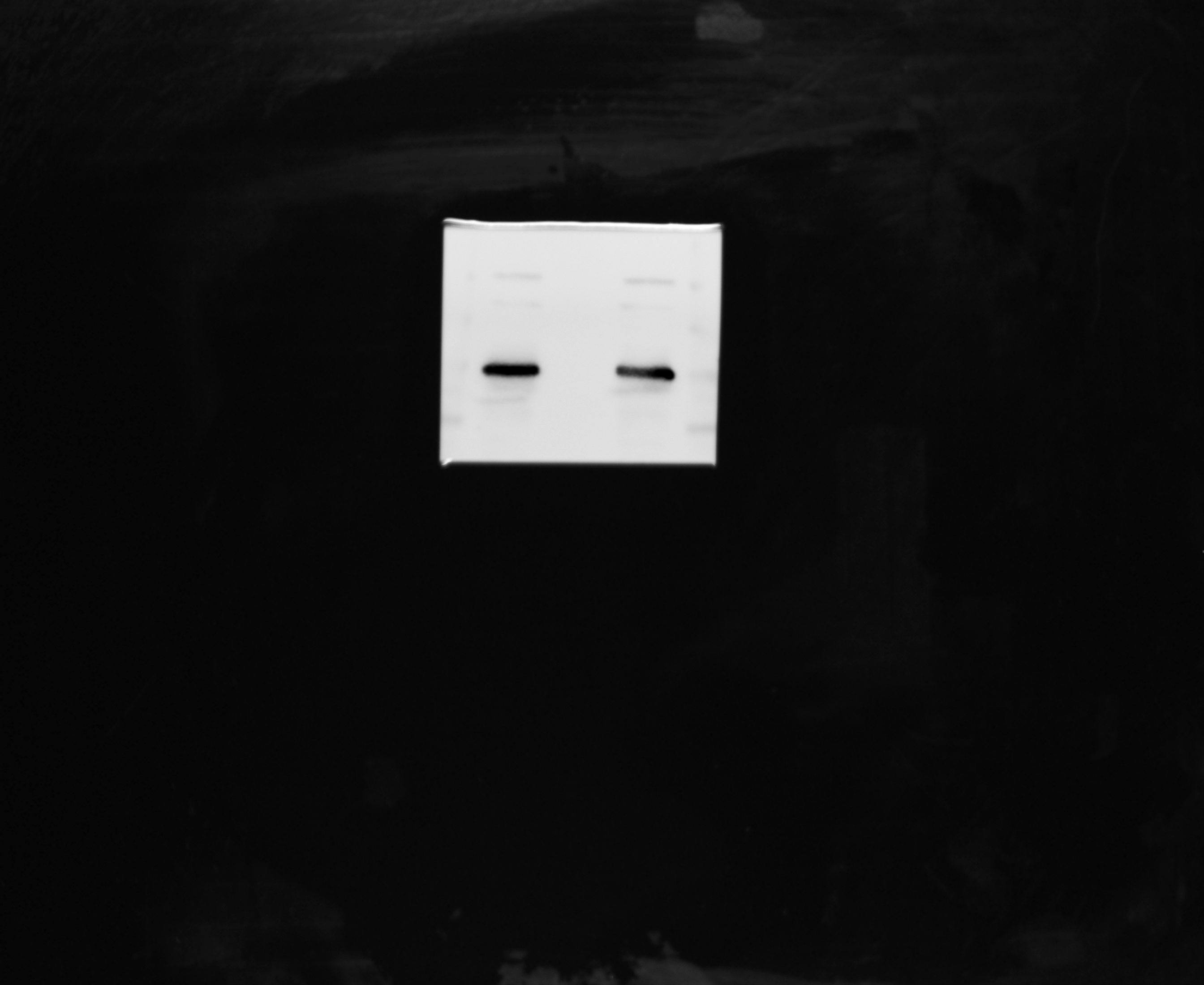


**KDa**

43

34

**Fig.5 H**

HA


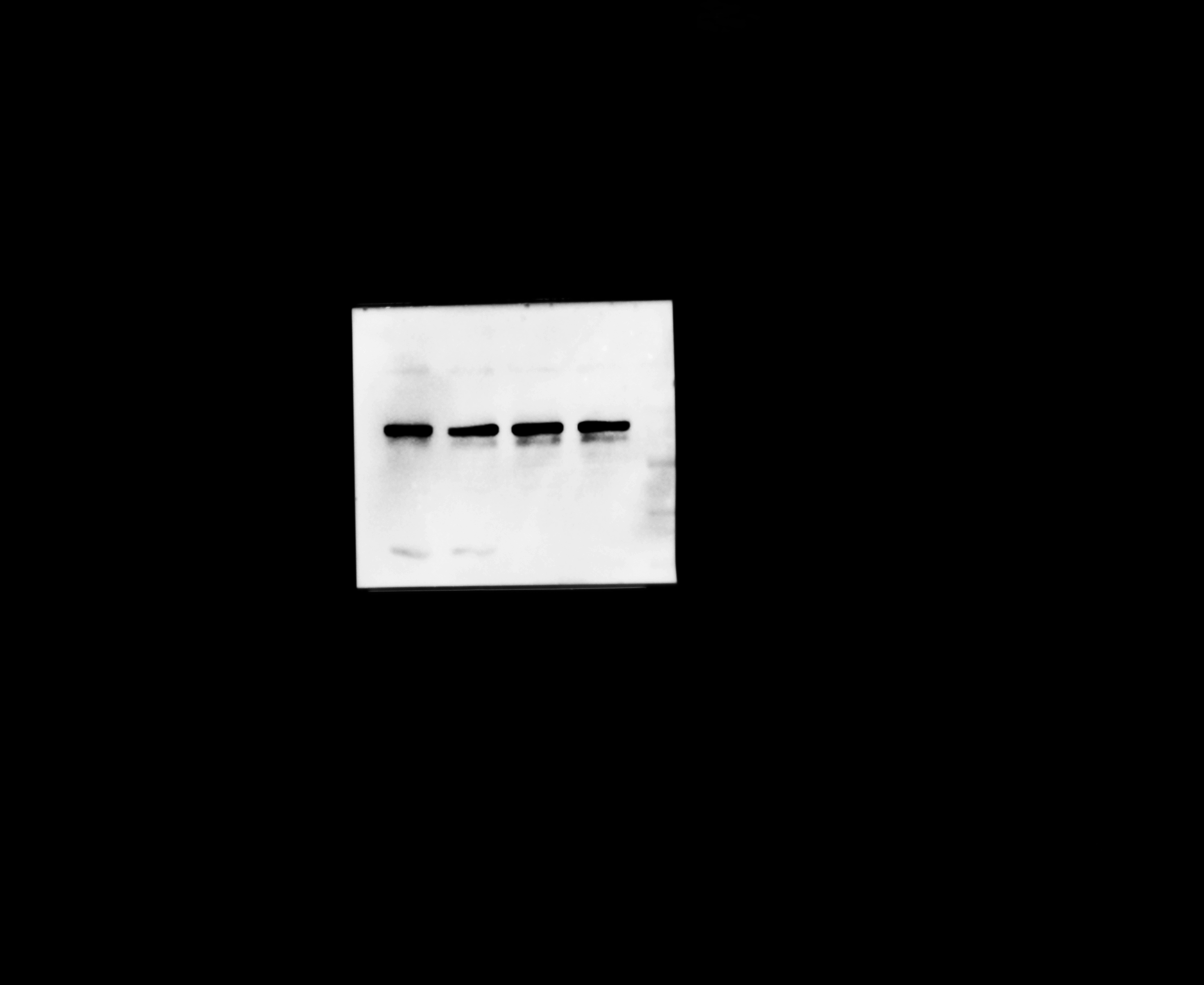


**KDa**

100

70

Flag


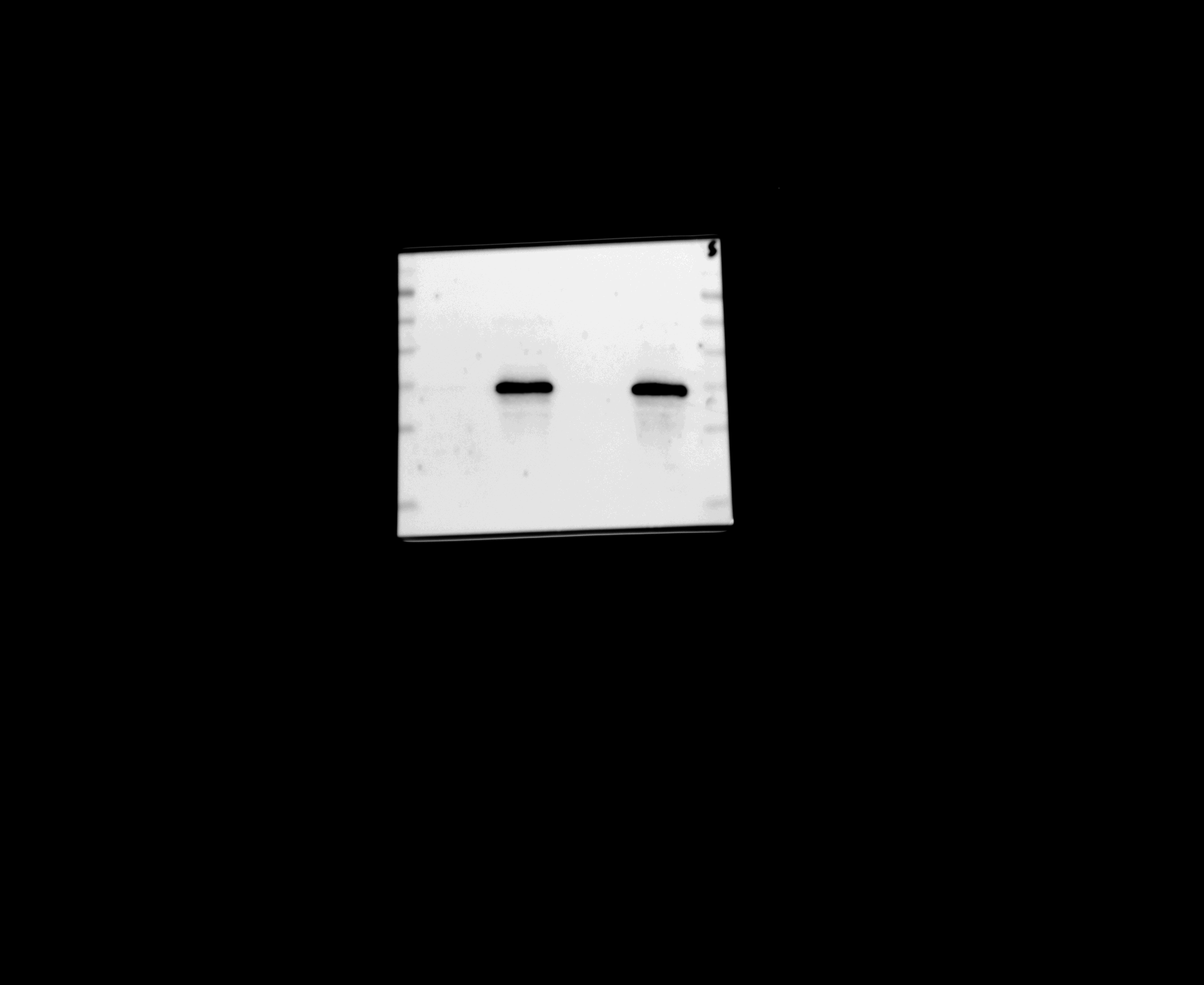


**KDa**

43

34

Flag


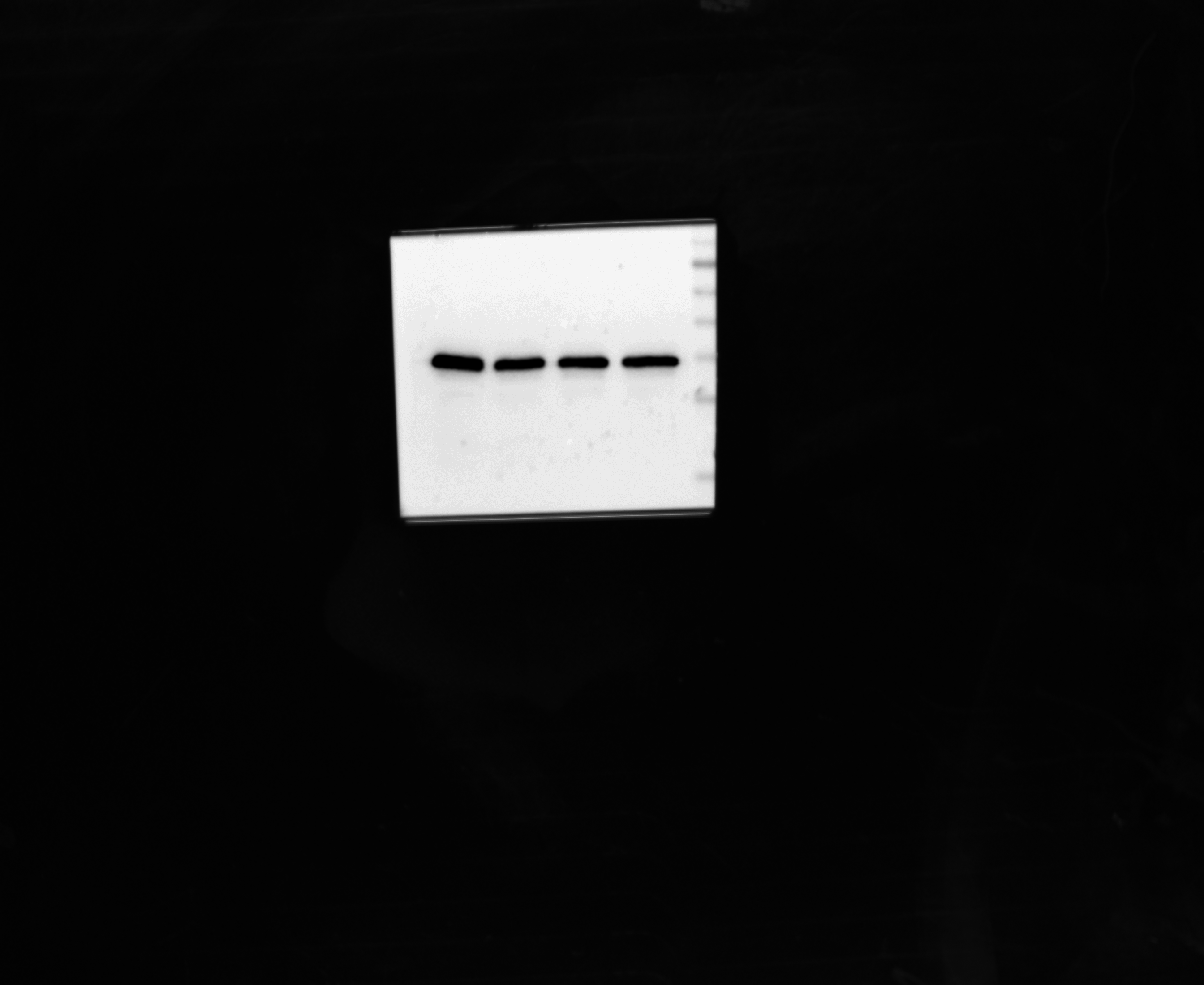


**KDa**

43

34

HA


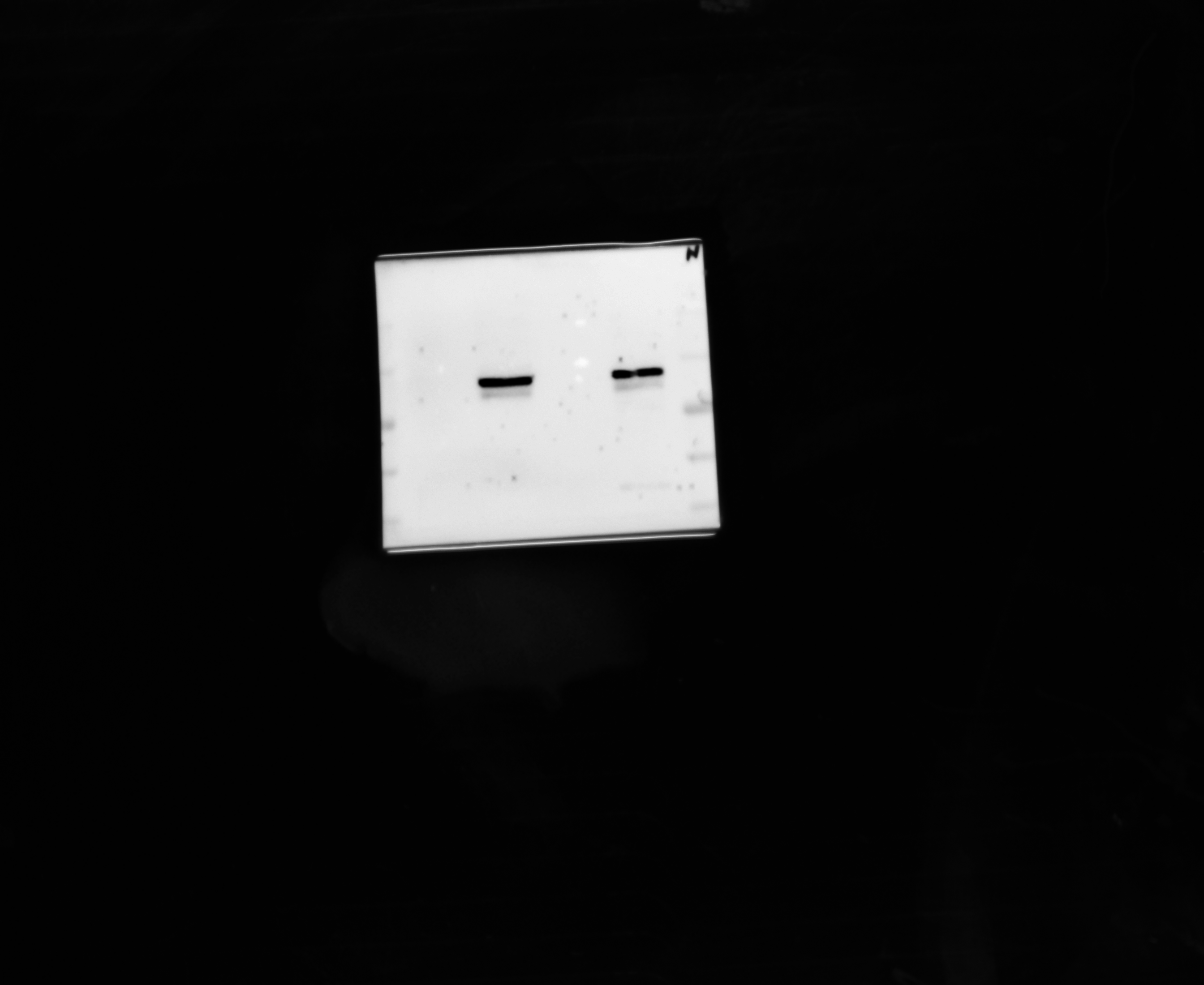


**KDa**

100

70

**Fig.5 L**

SOX2


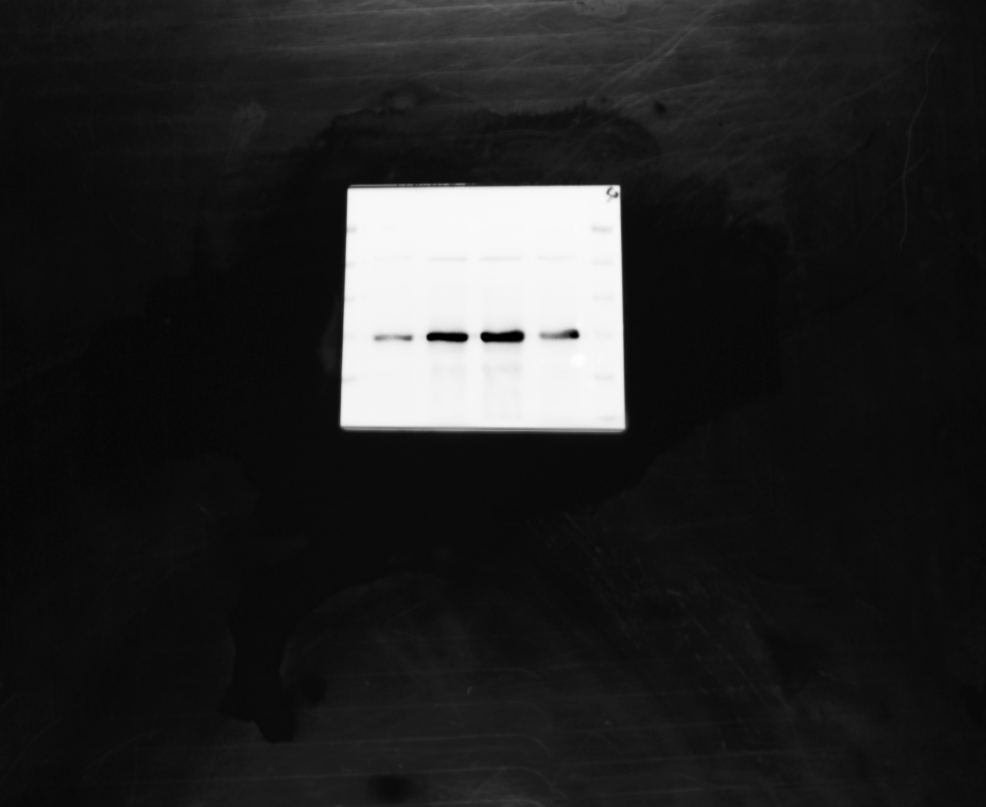


**KDa**

43

34

SOX2


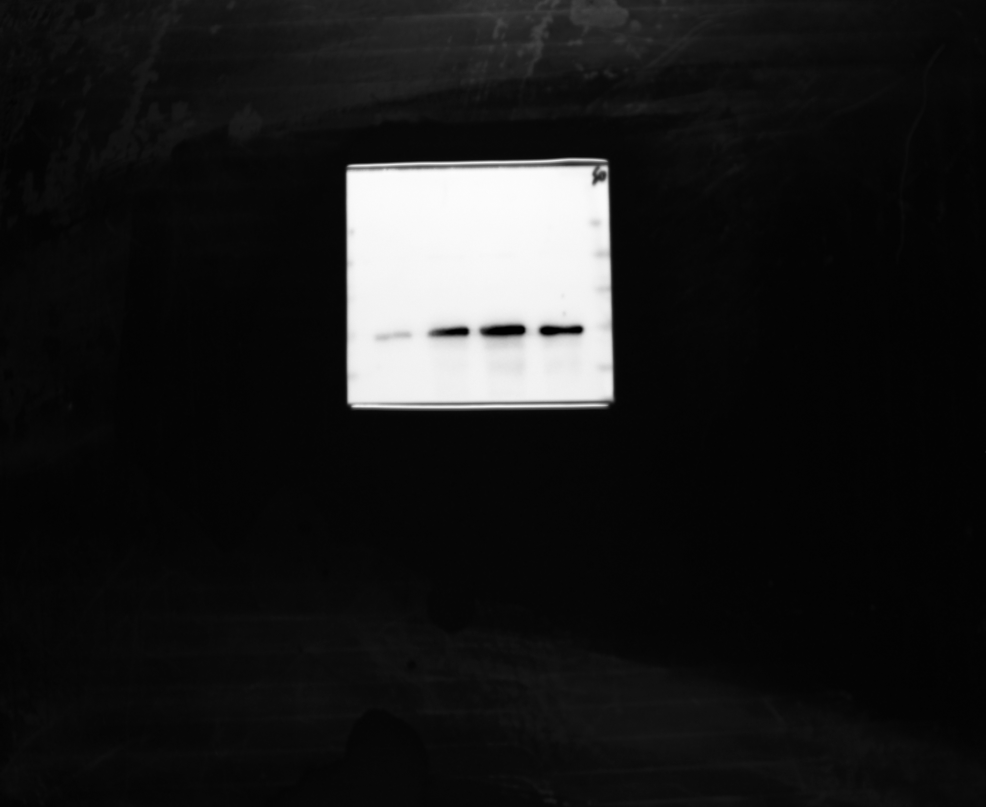


**KDa**

43

34

Lamin B1


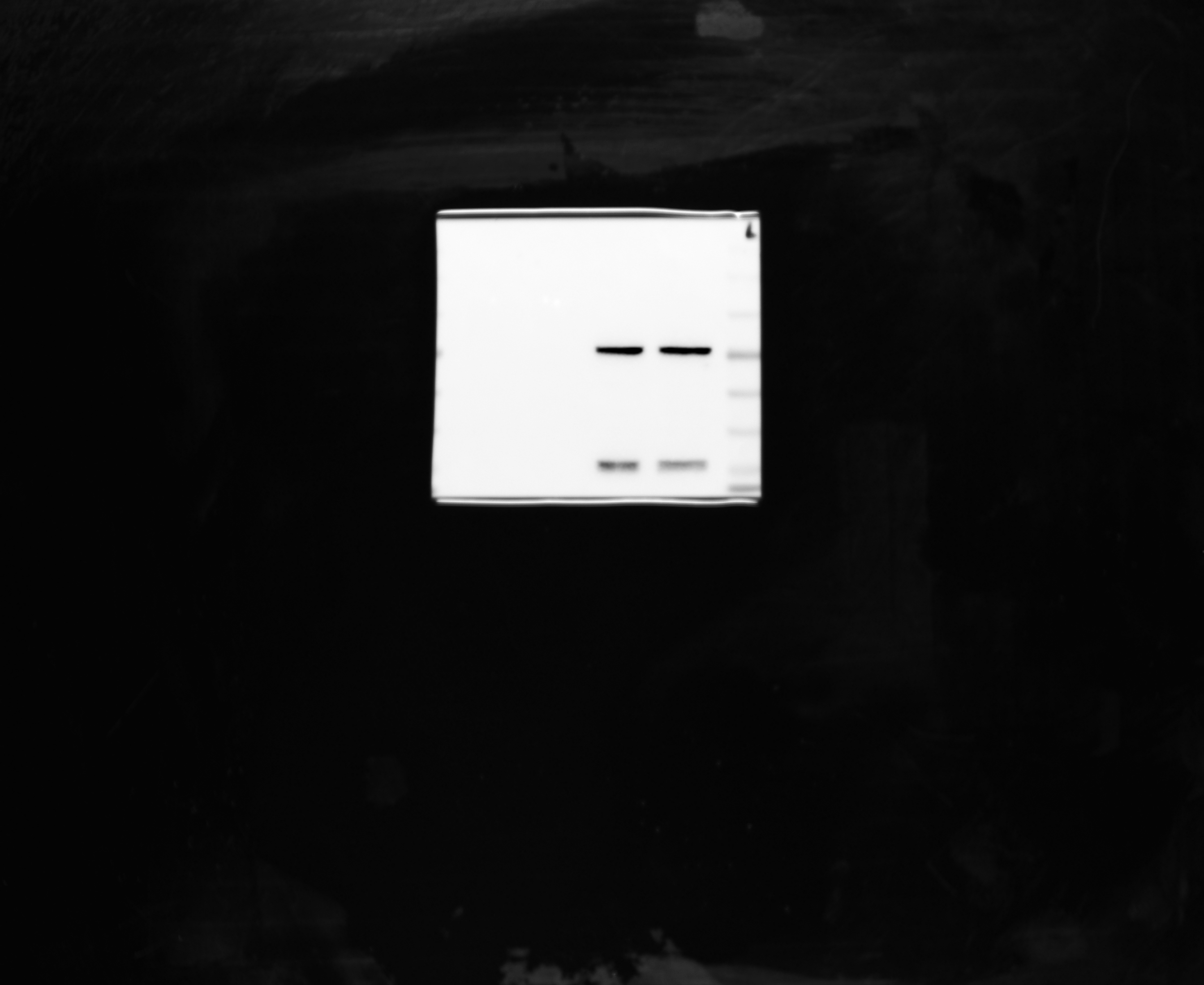


**KDa**

100

70

Lamin B1


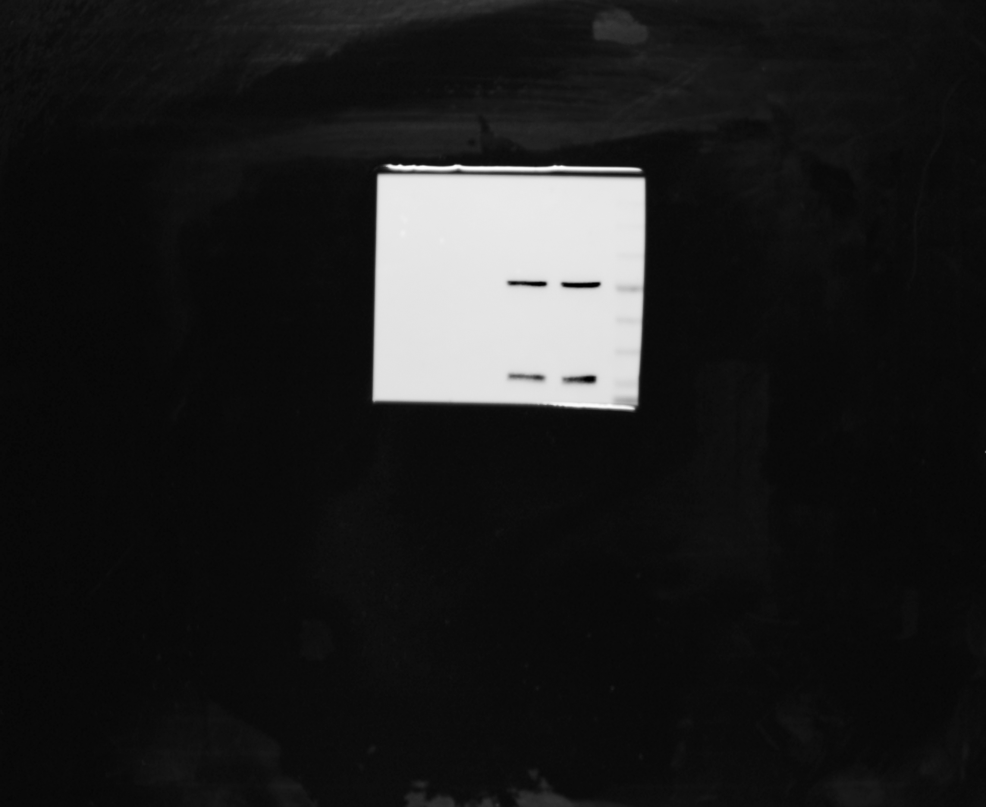


**KDa**

100

70

β-Tubulin


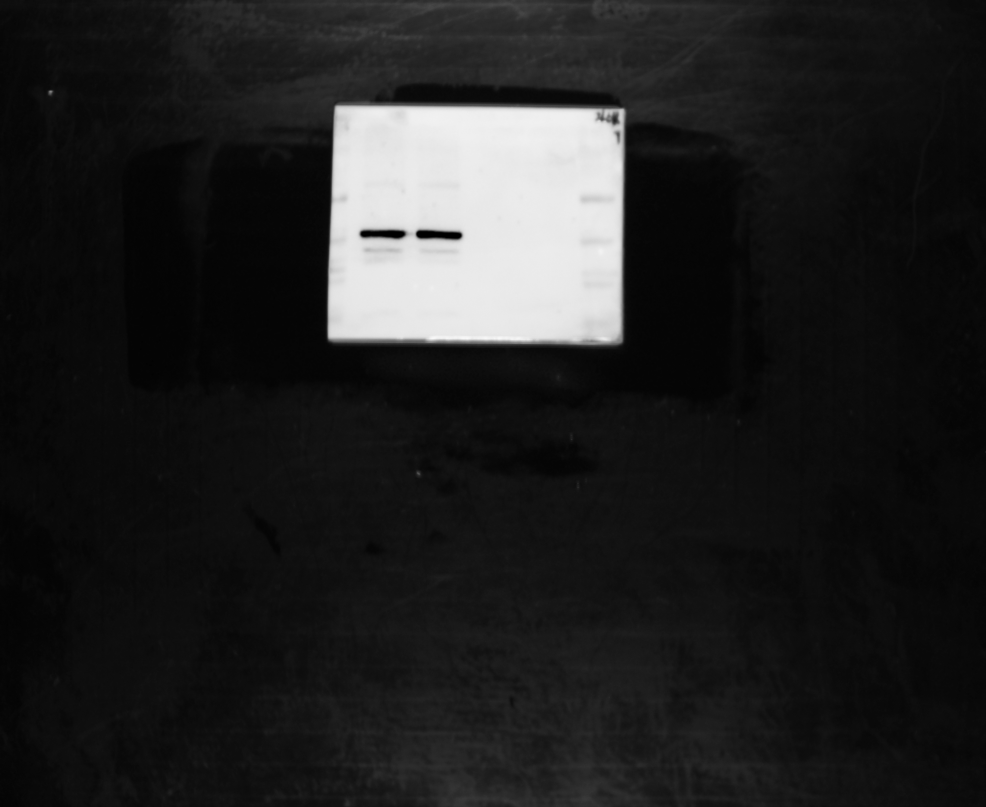


**KDa**

70

55

β-Tubulin


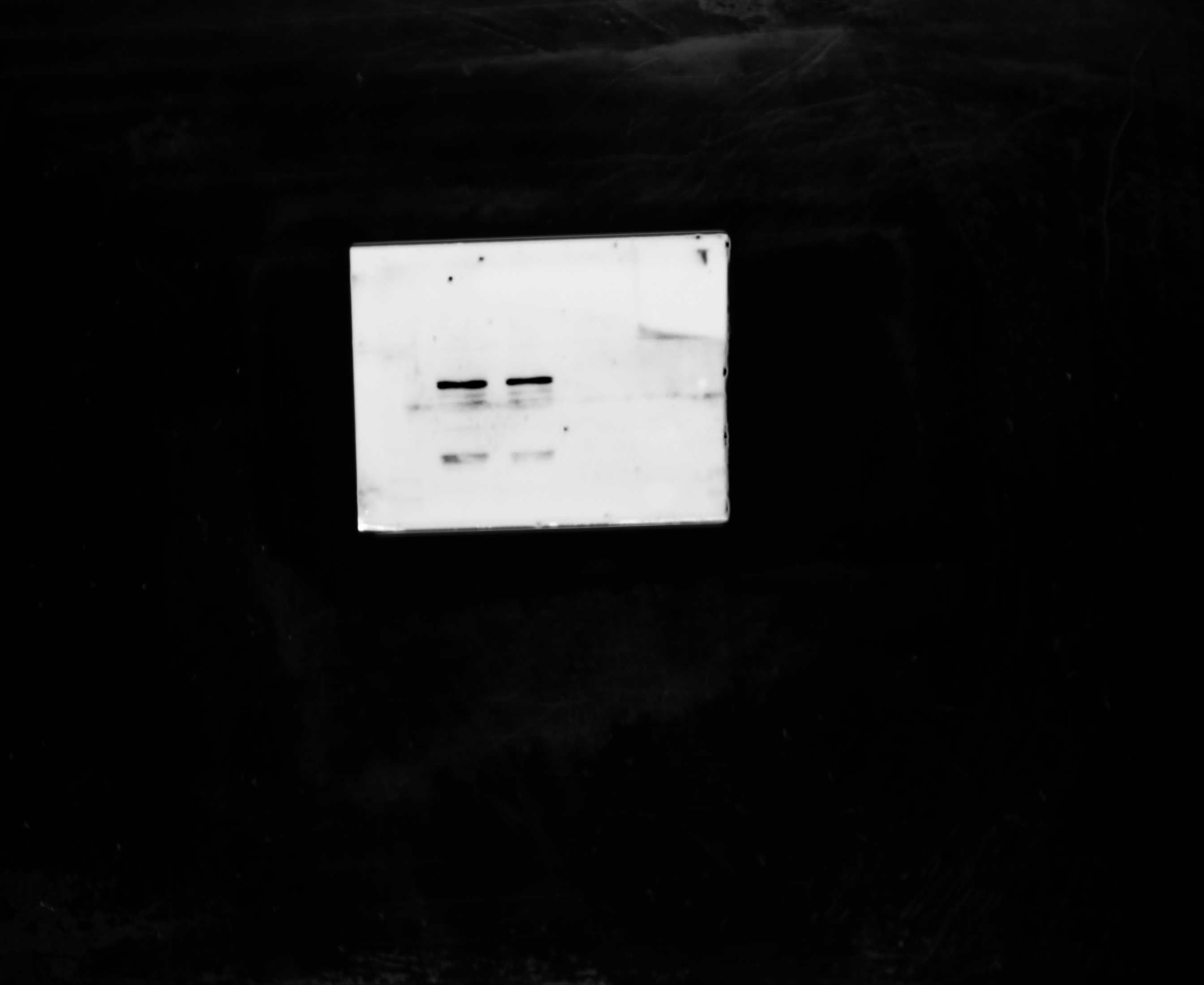


**KDa**

70

55

**Fig.6 C**

HA


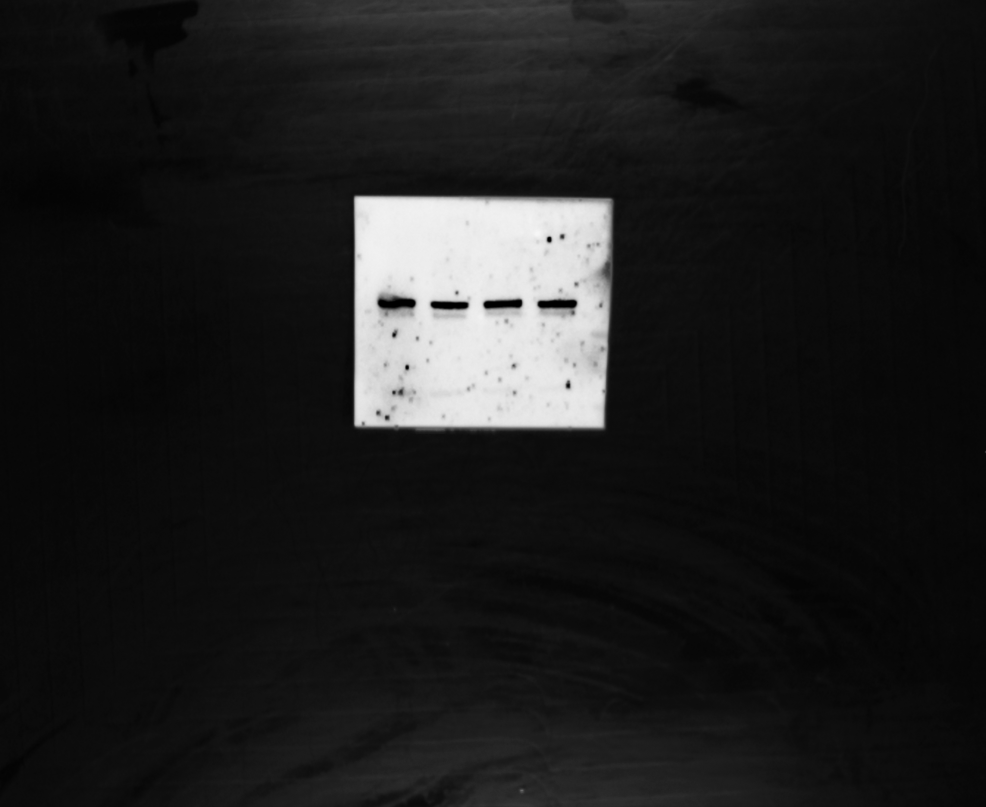


**KDa**

100

70

Flag


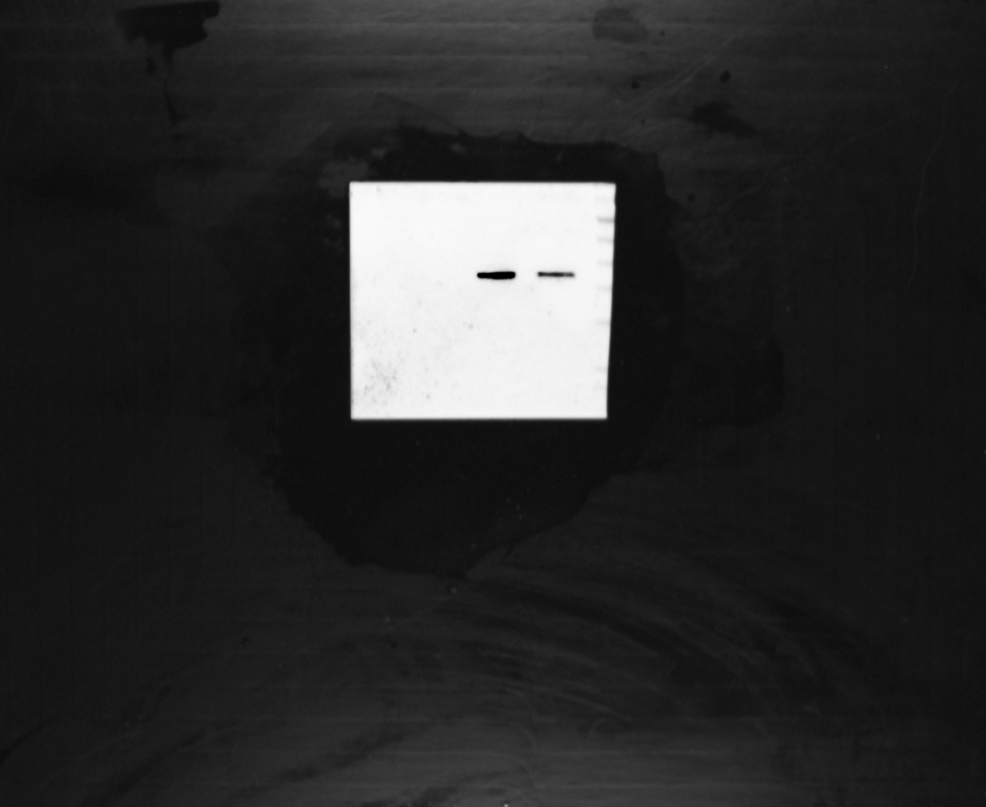


**KDa**

43

34

HA


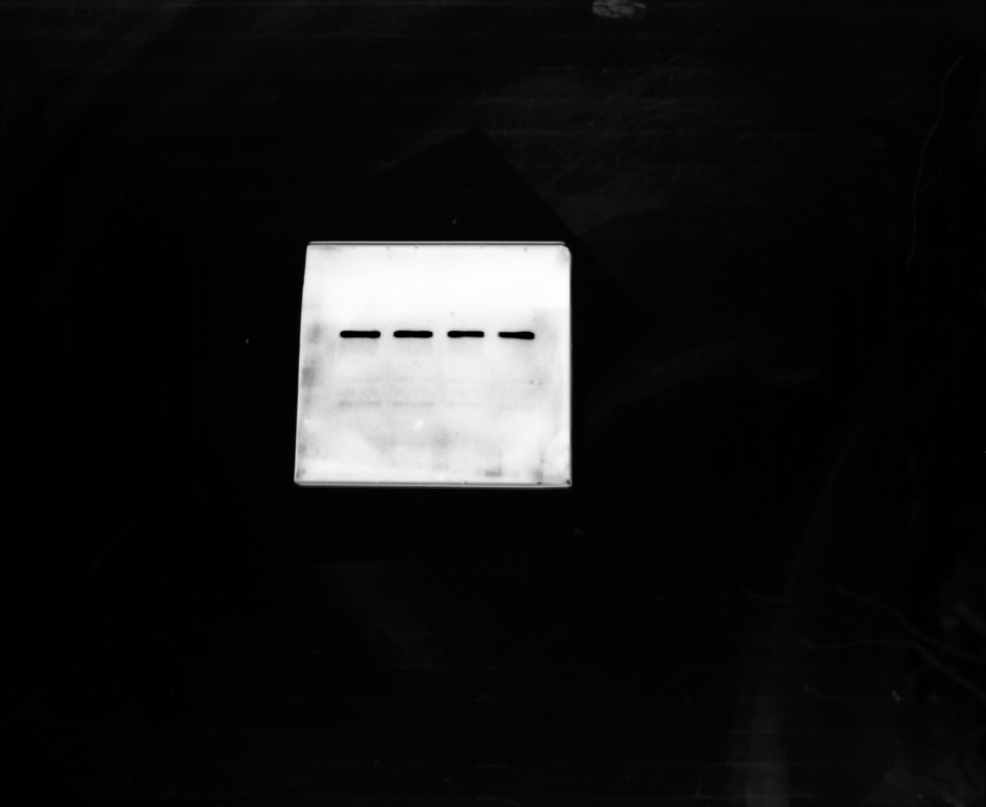


**KDa**

100

70

Flag


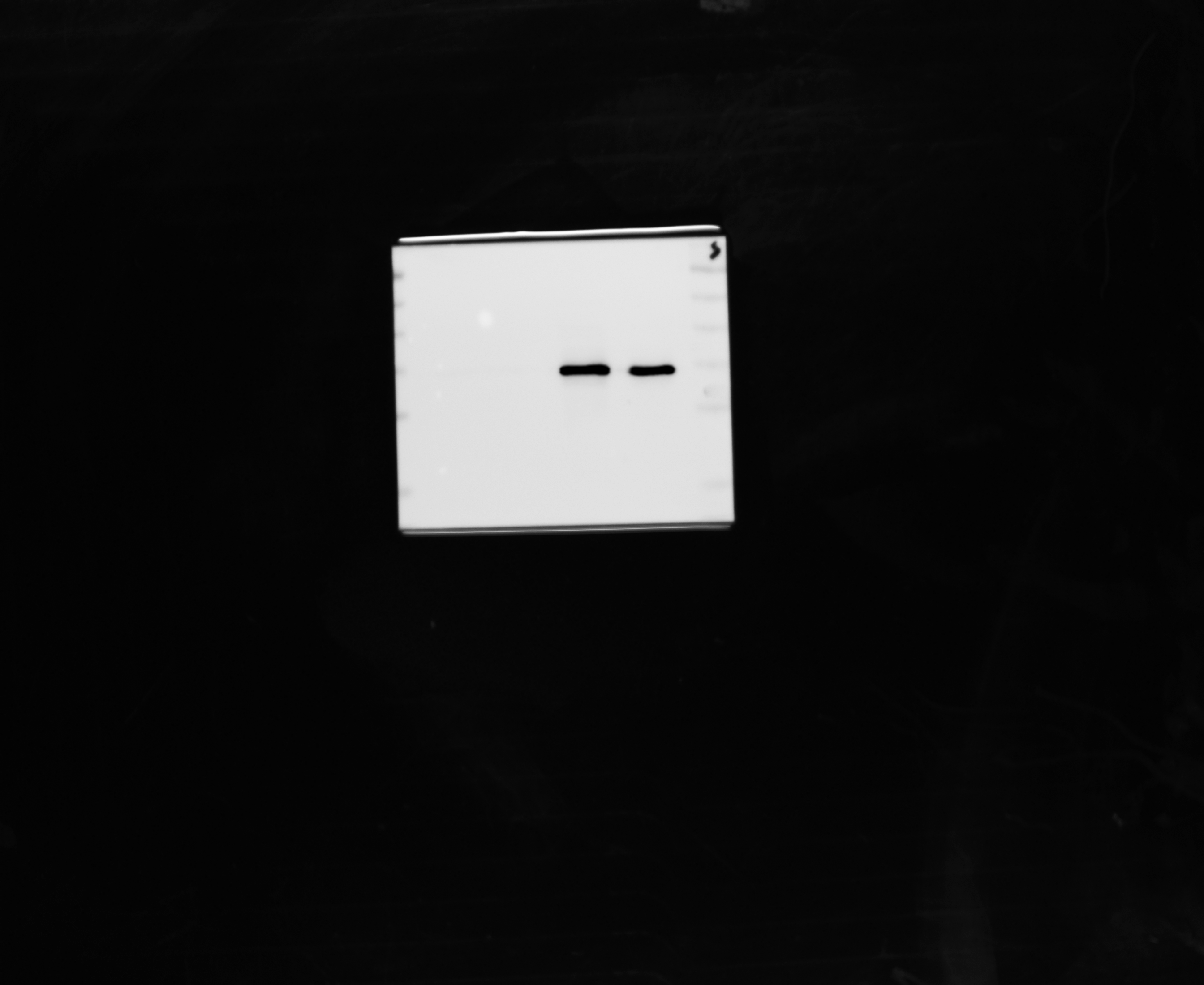


**KDa**

43

34

Flag


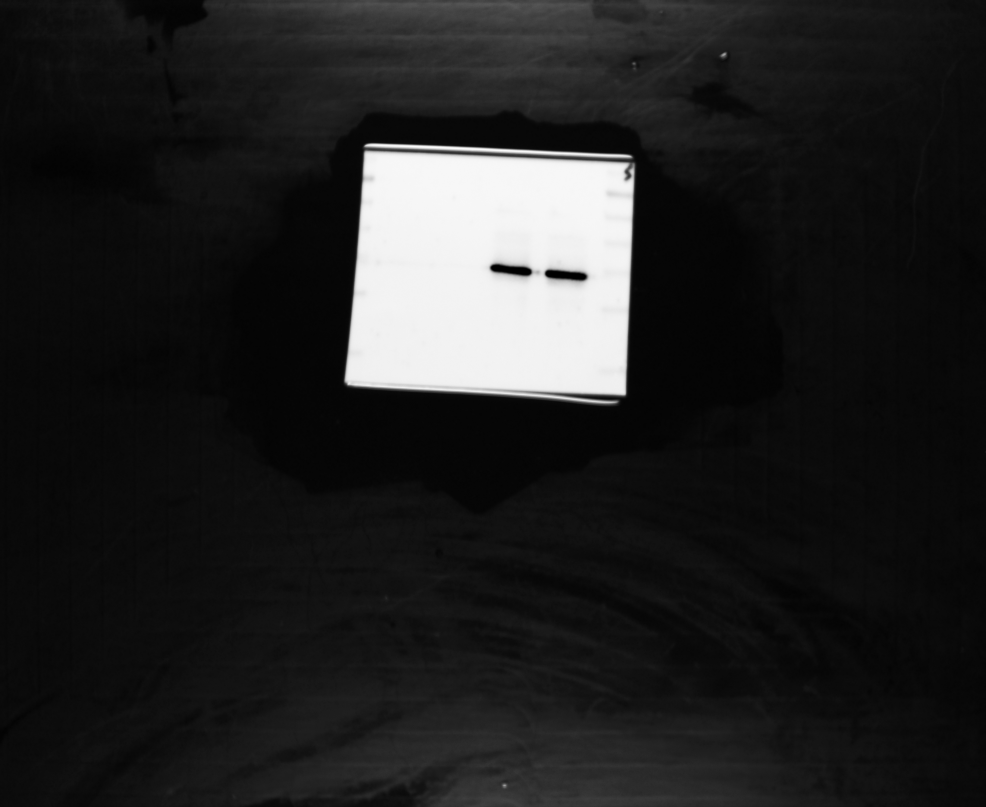


**KDa**

43

34

HA


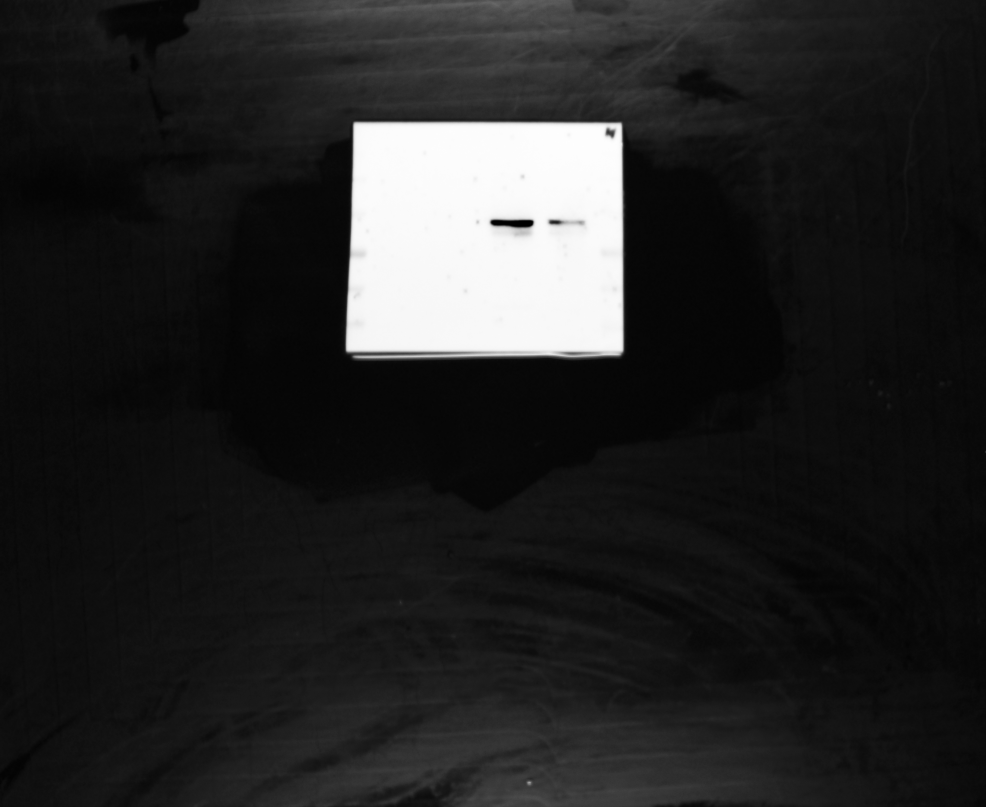


**KDa**

100

70

Falg


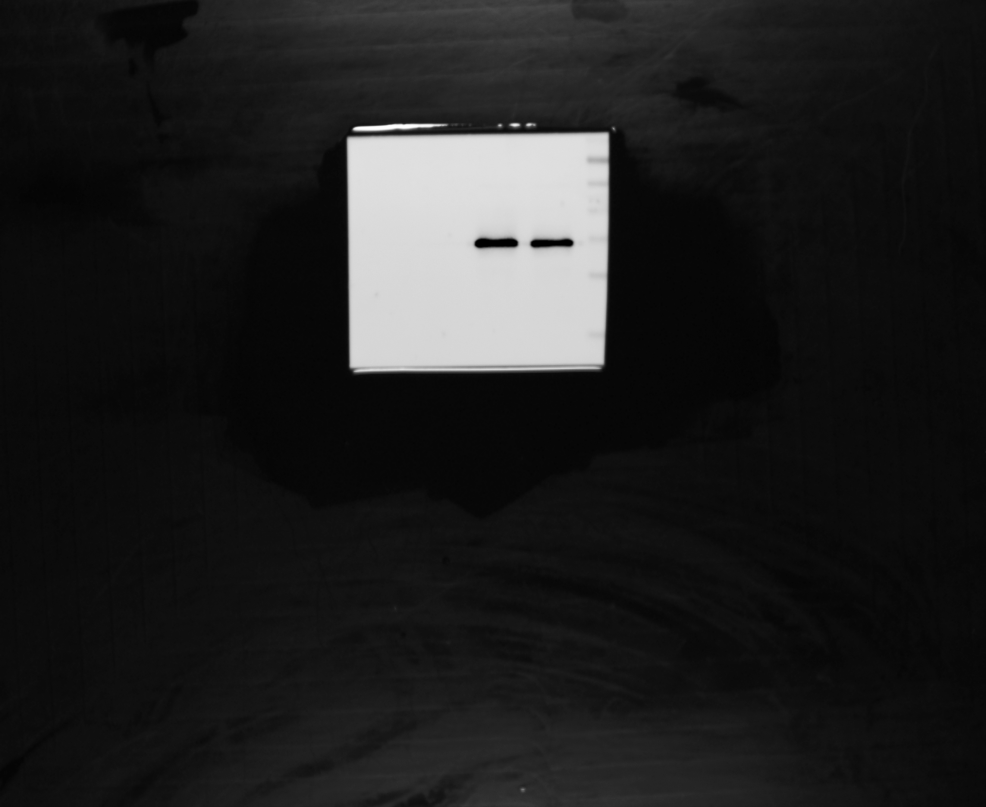


**KDa**

43

34

HA


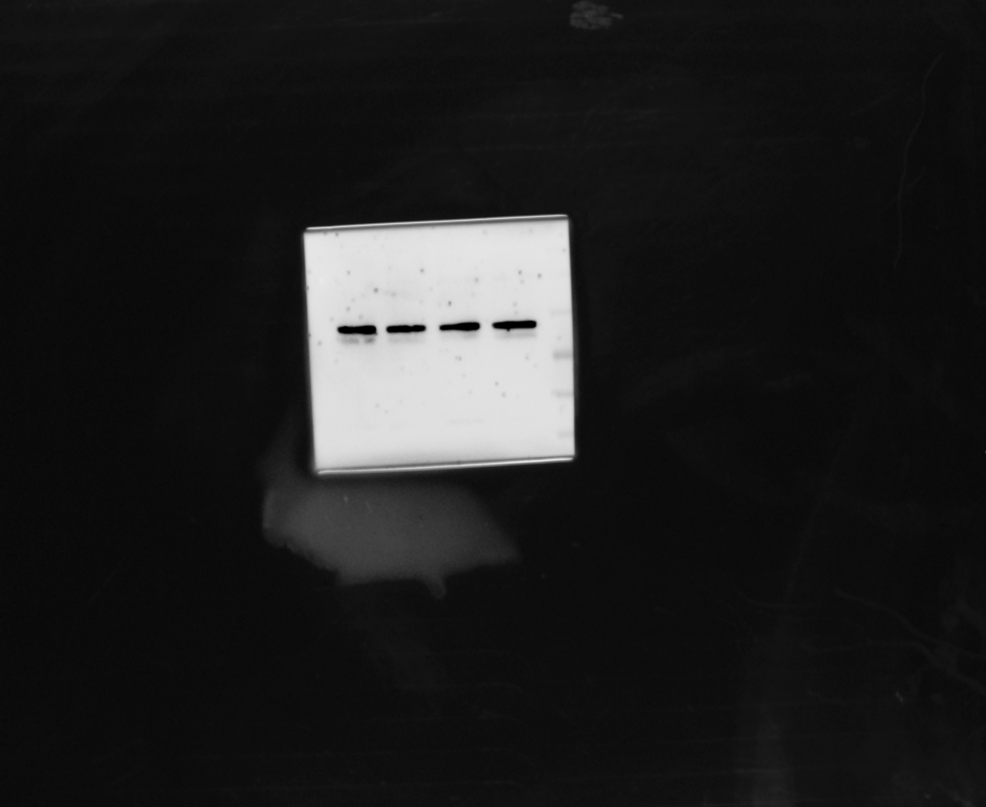


**KDa**

100

70

**Fig.6 D**

HA


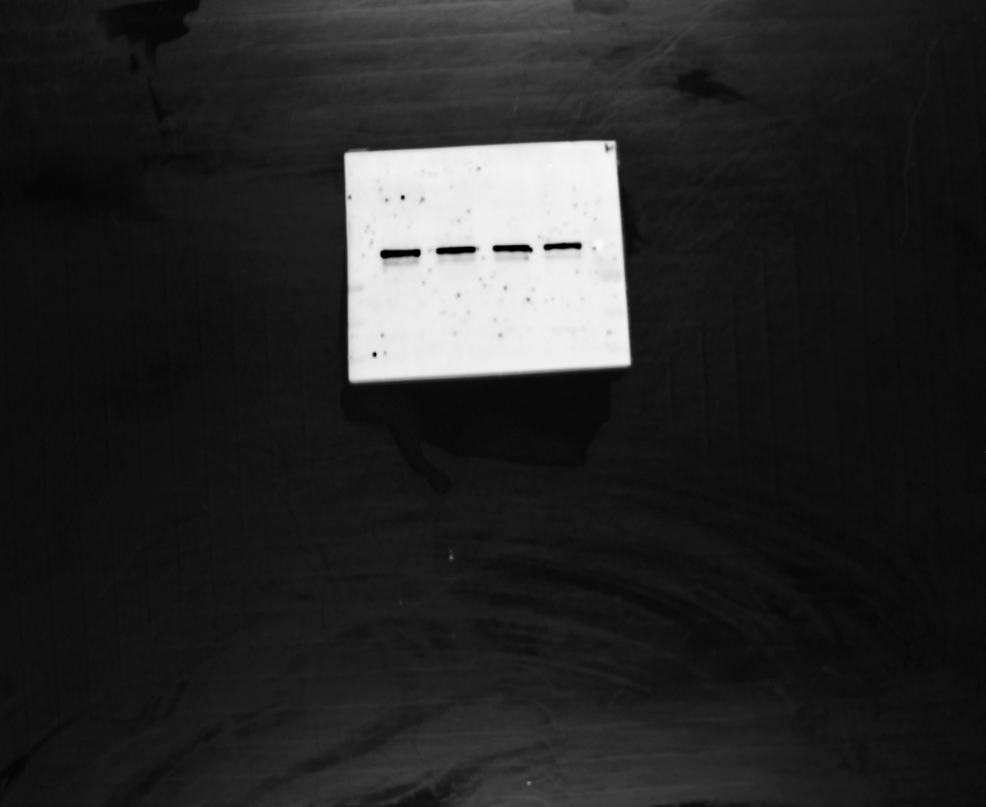


**KDa**

100

70

Flag


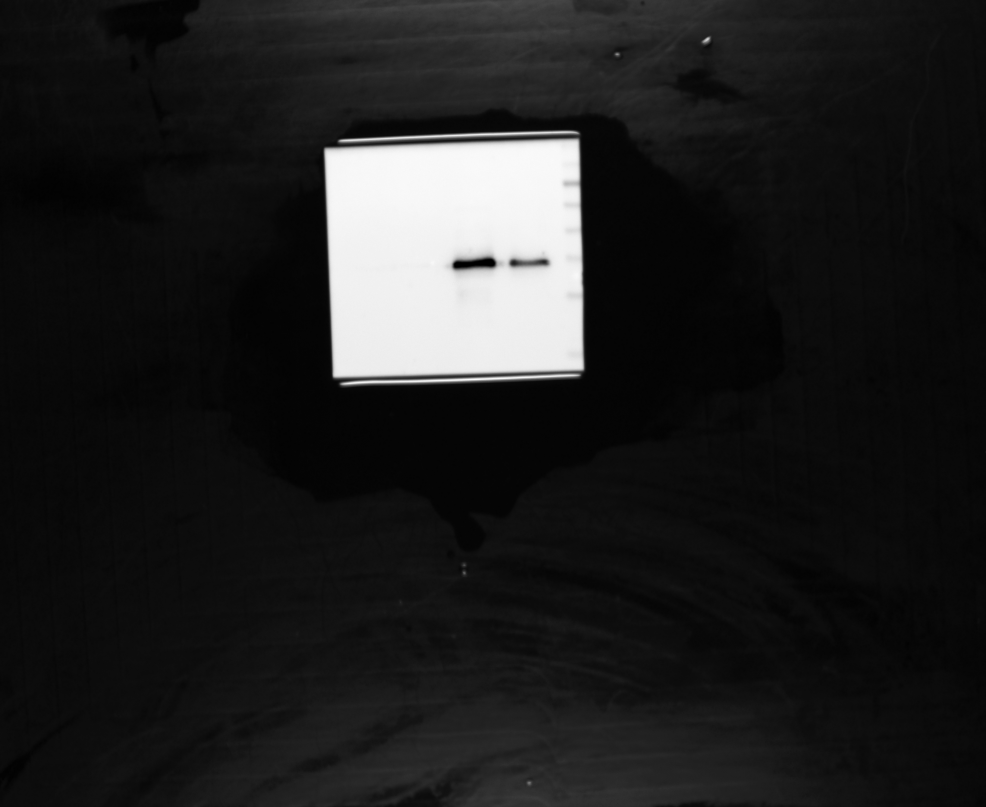


**KDa**

43

34

HA


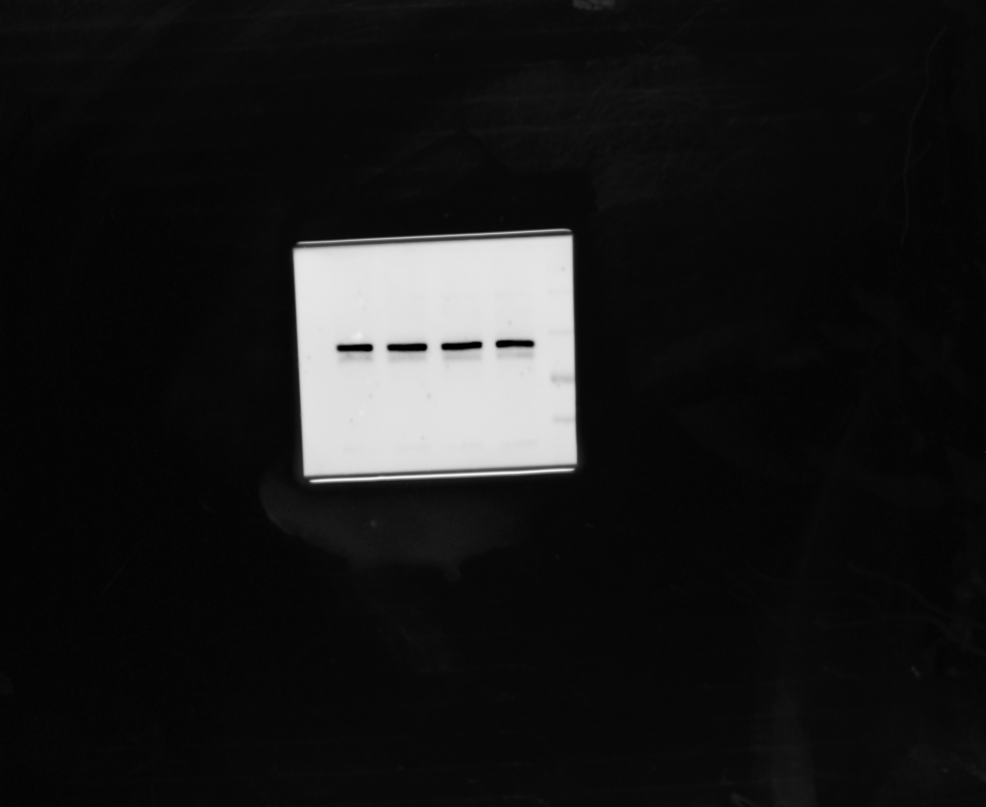


100

70

**KDa**

Flag


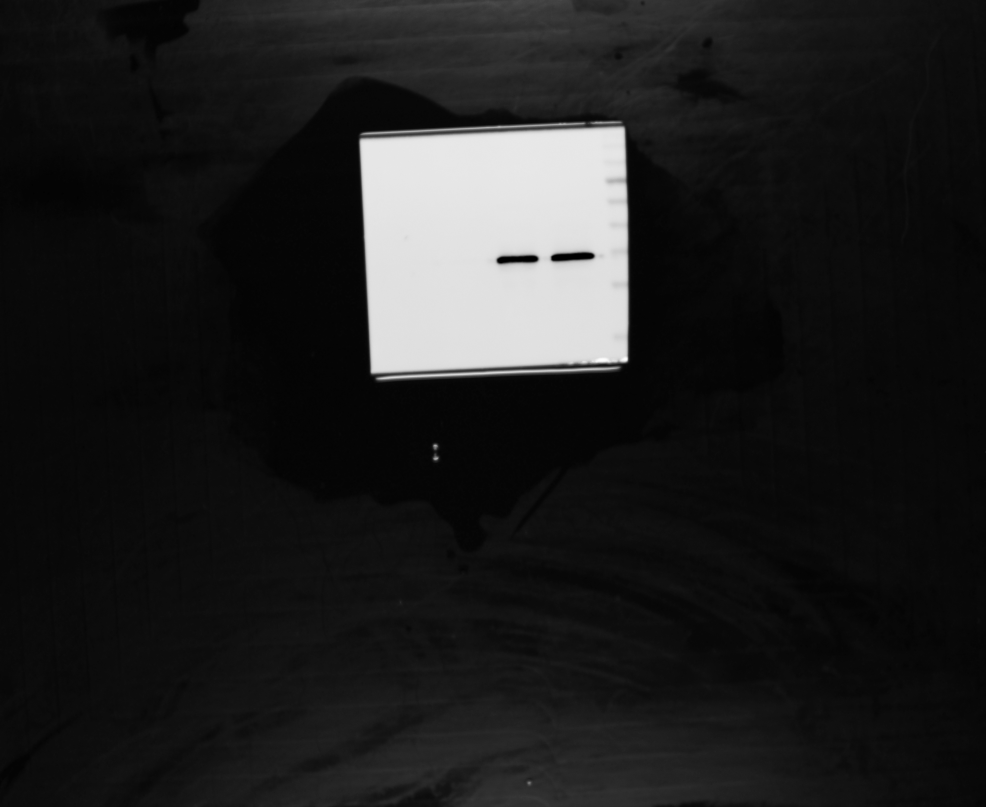


**KDa**

43

34

Flag


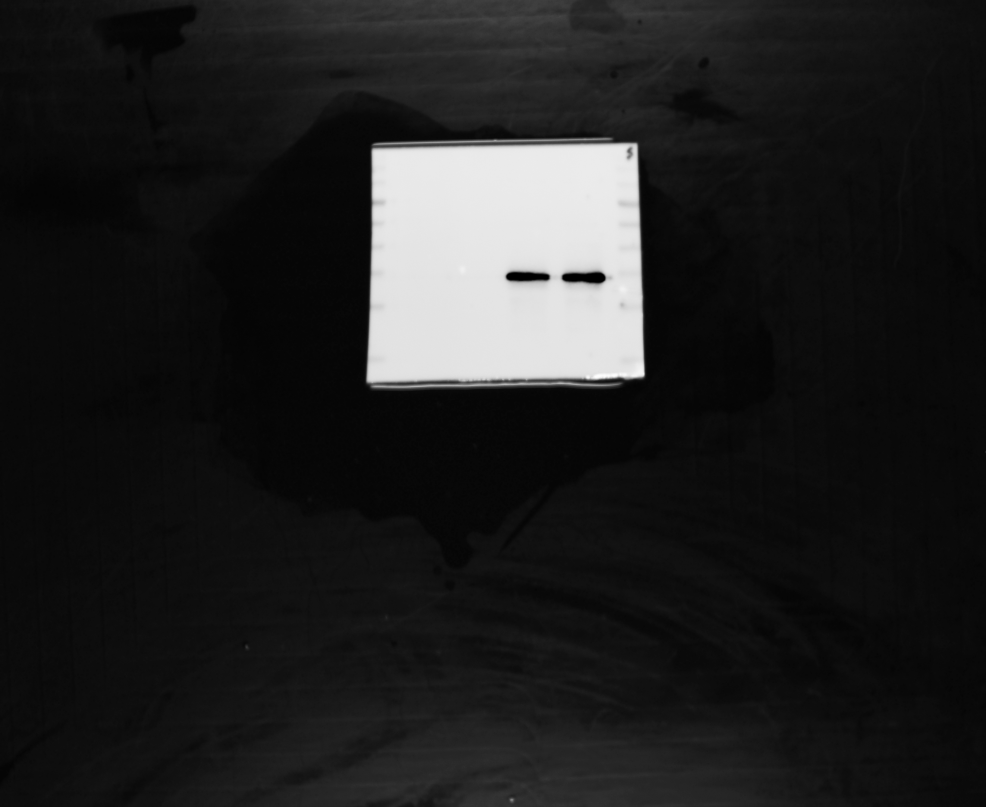


**KDa**

43

34

HA


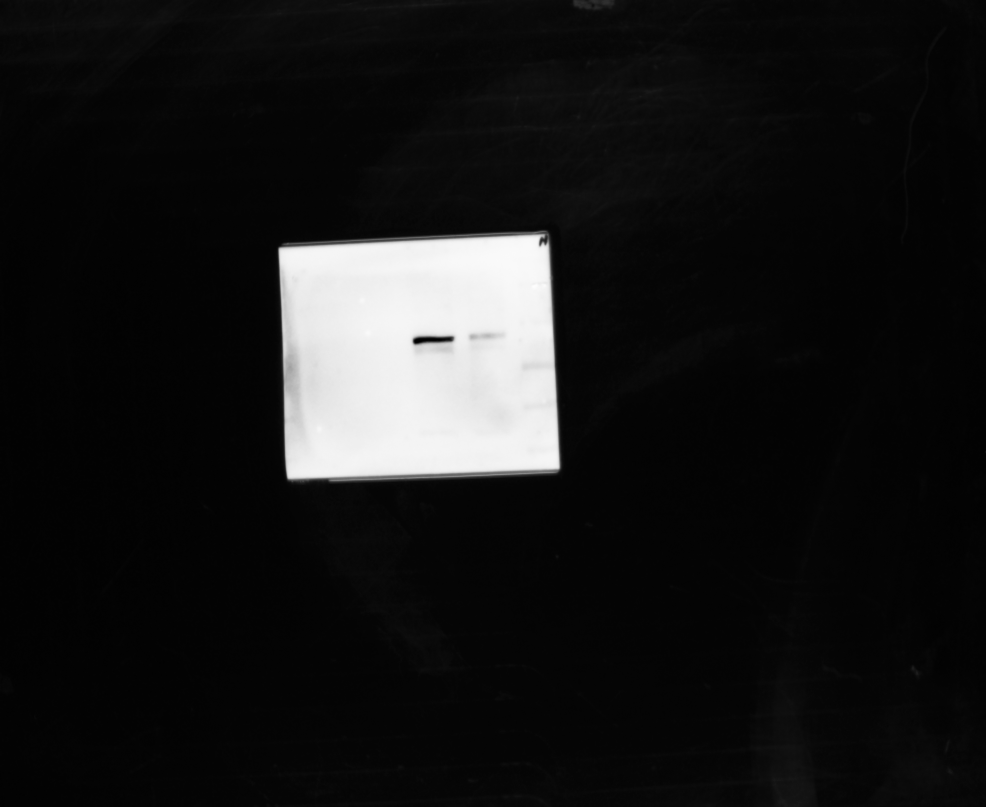


100

70

**KDa**

Falg


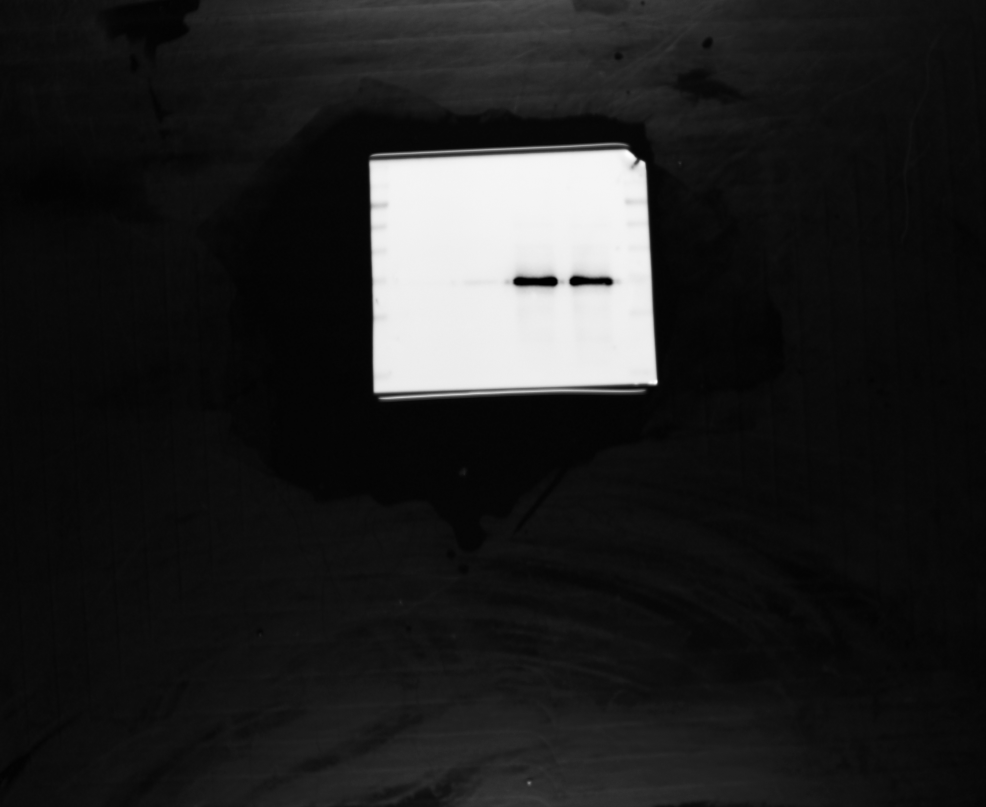


**KDa**

43

34

HA


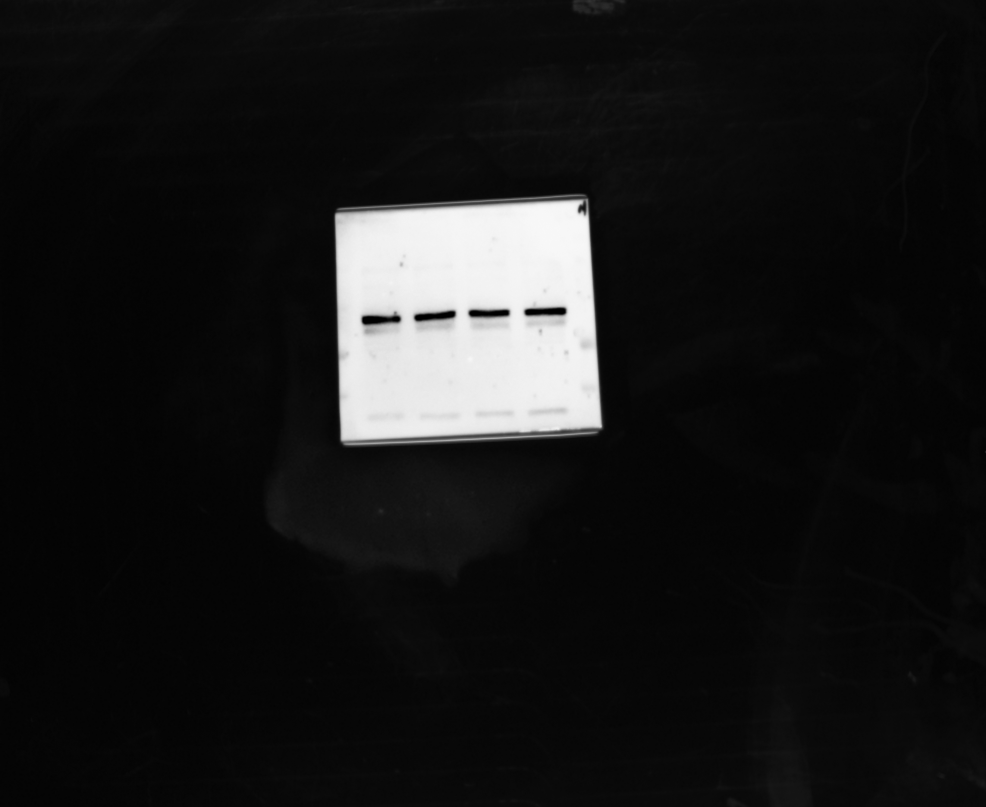


**KDa**

100

70

**Fig.6 F**

Flag


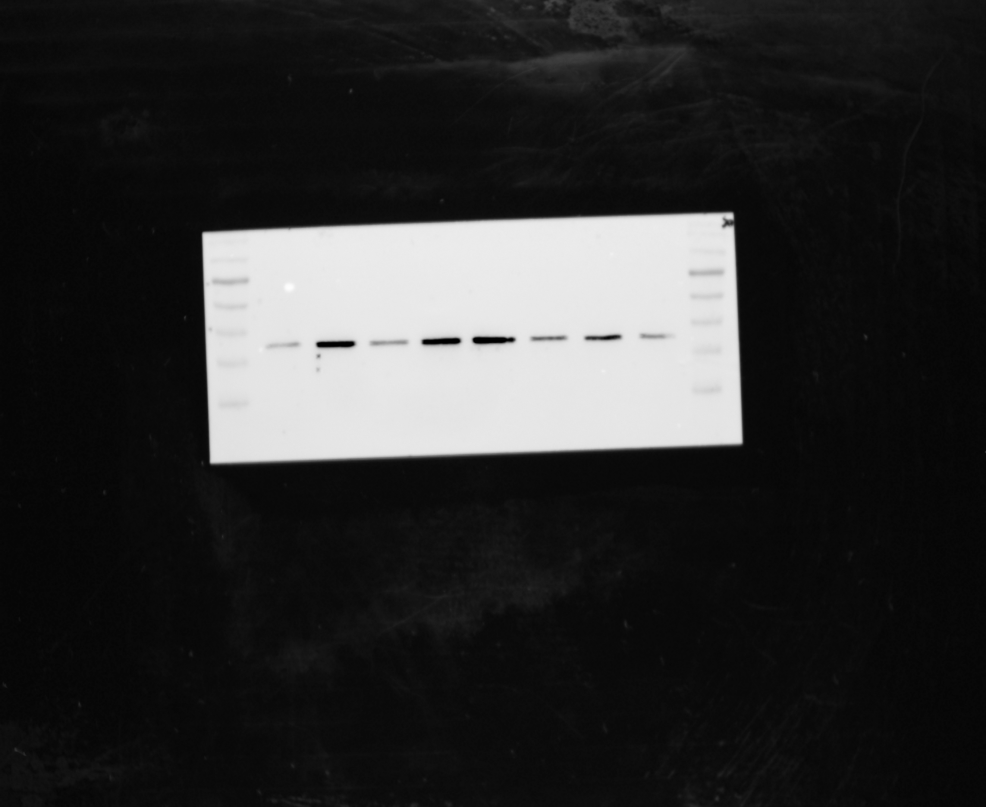


**KDa**

43

34

Flag


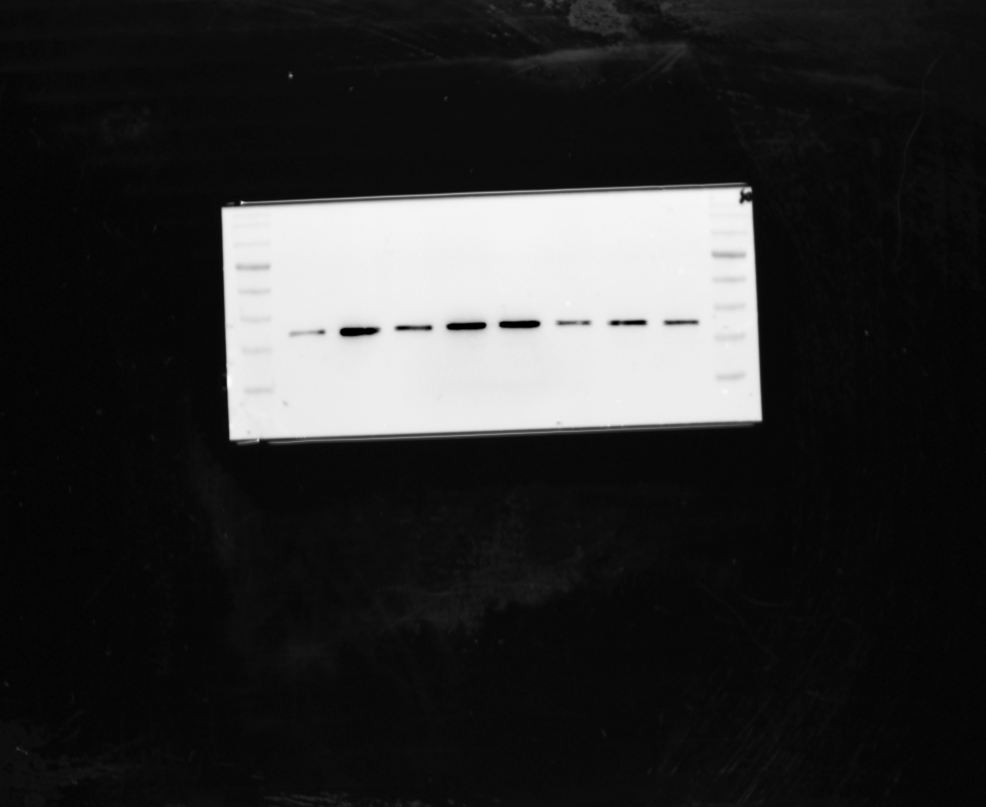


**KDa**

43

34

Lamin B1


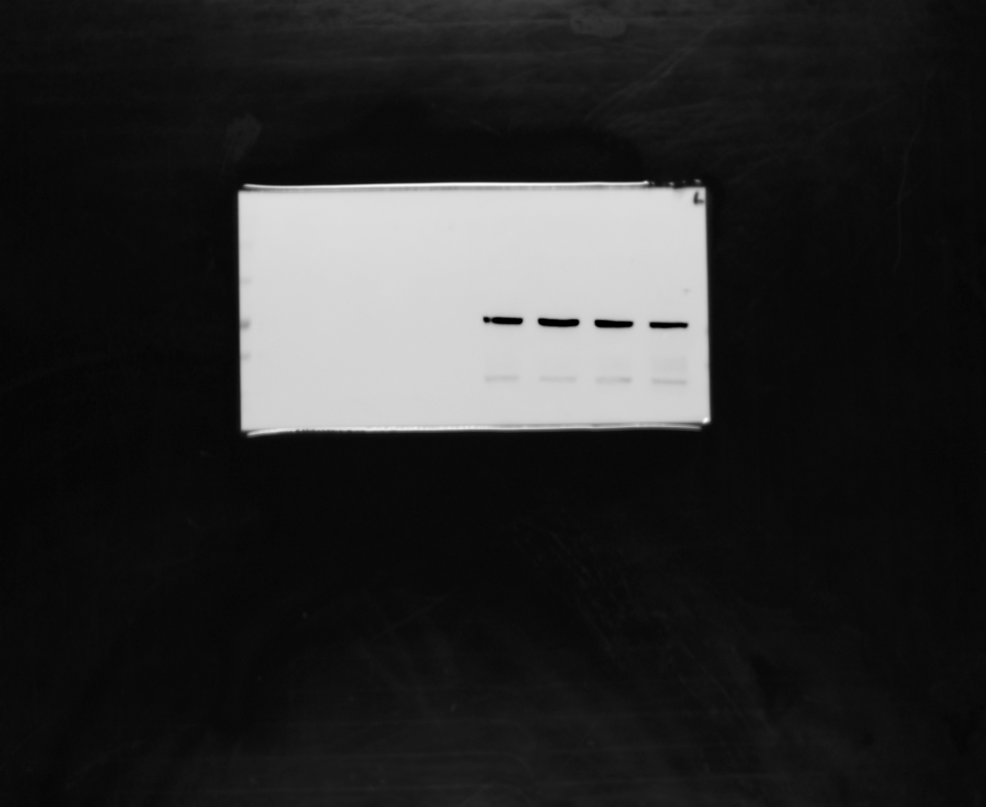


**KDa**

100

70

Lamin B1


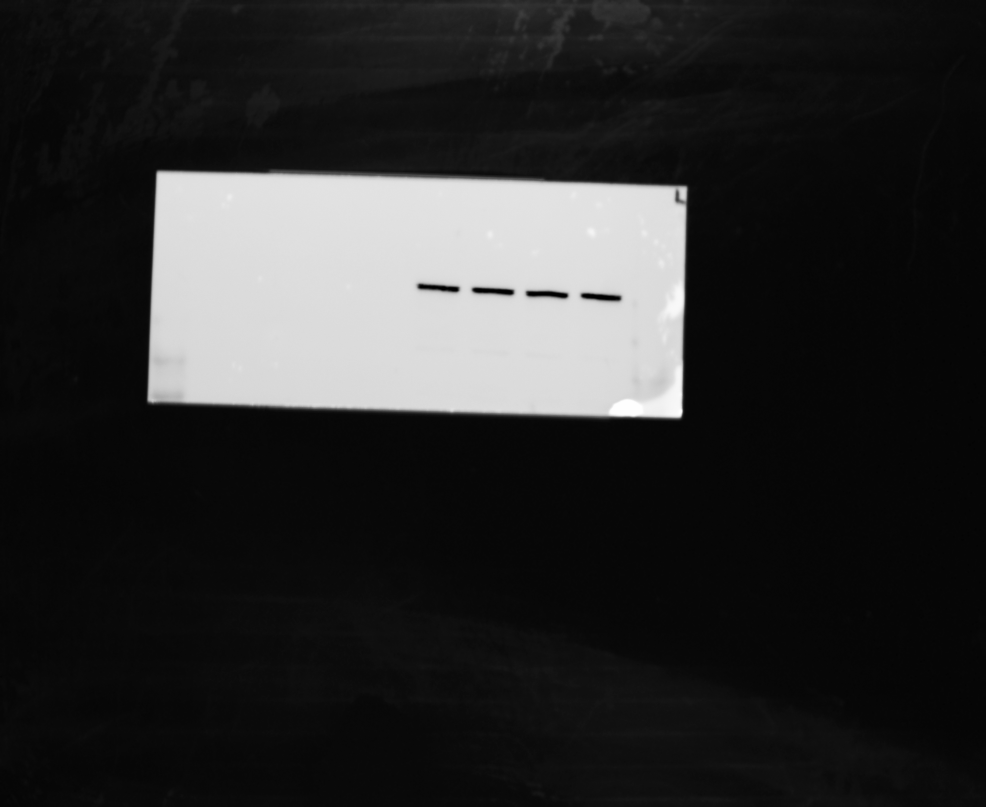


**KDa**

100

70

β-Tubulin


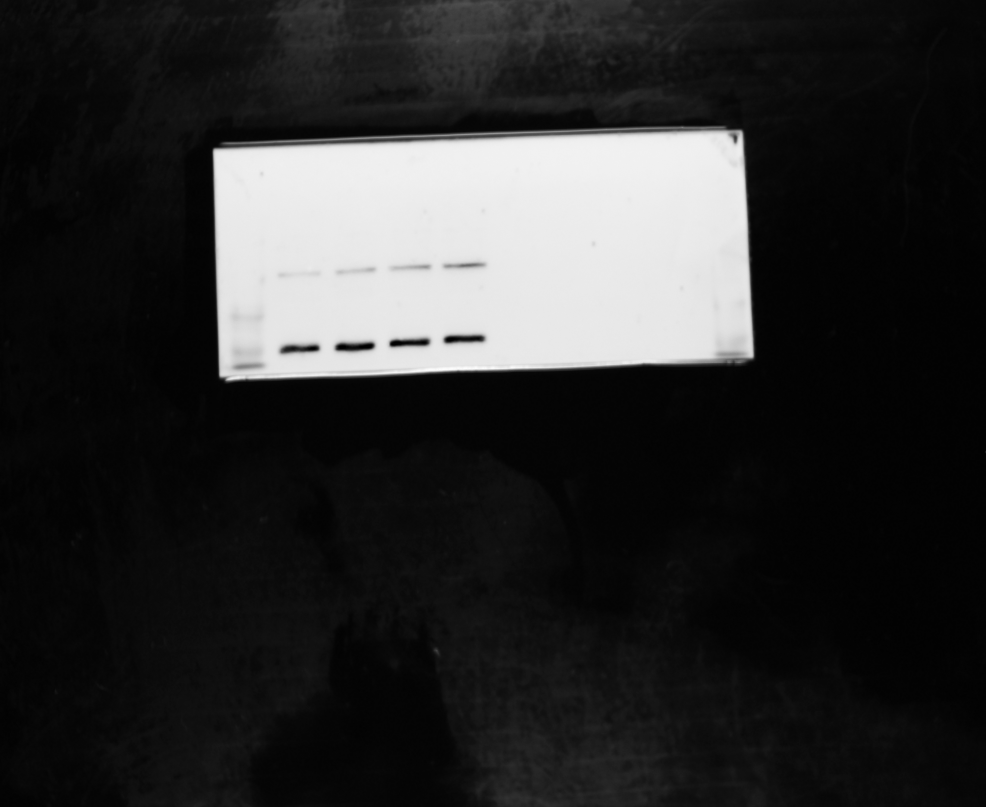


**KDa**

70

55

β-Tubulin


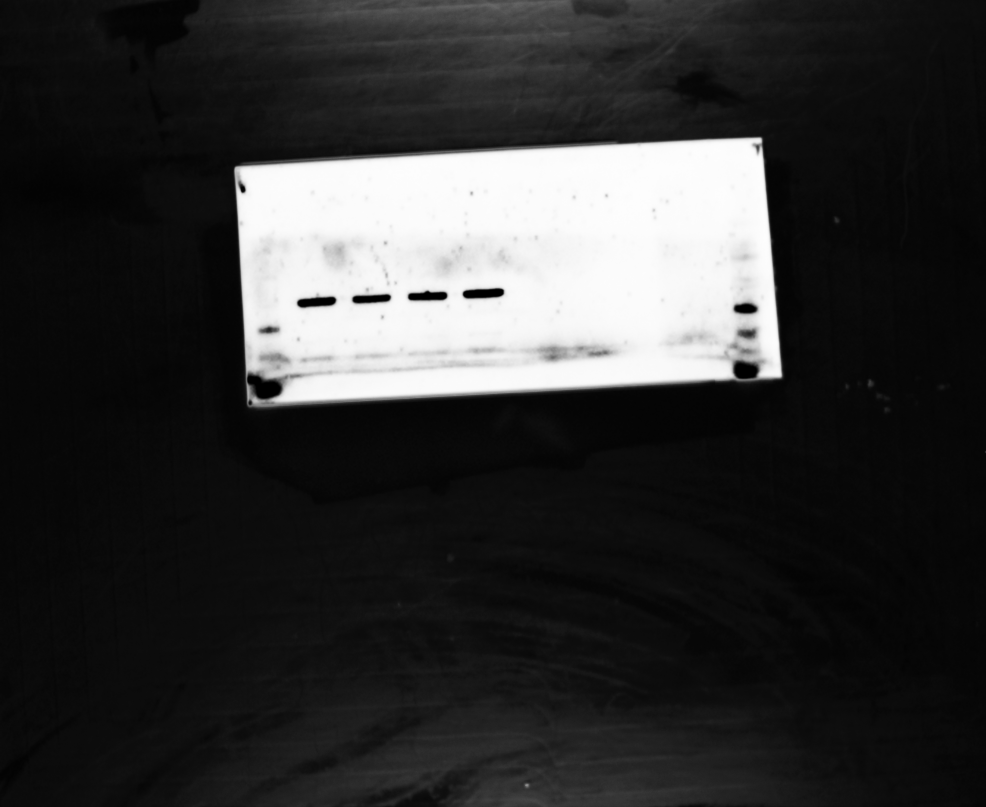


**KDa**

70

55

**Fig.7 G**

SOX2


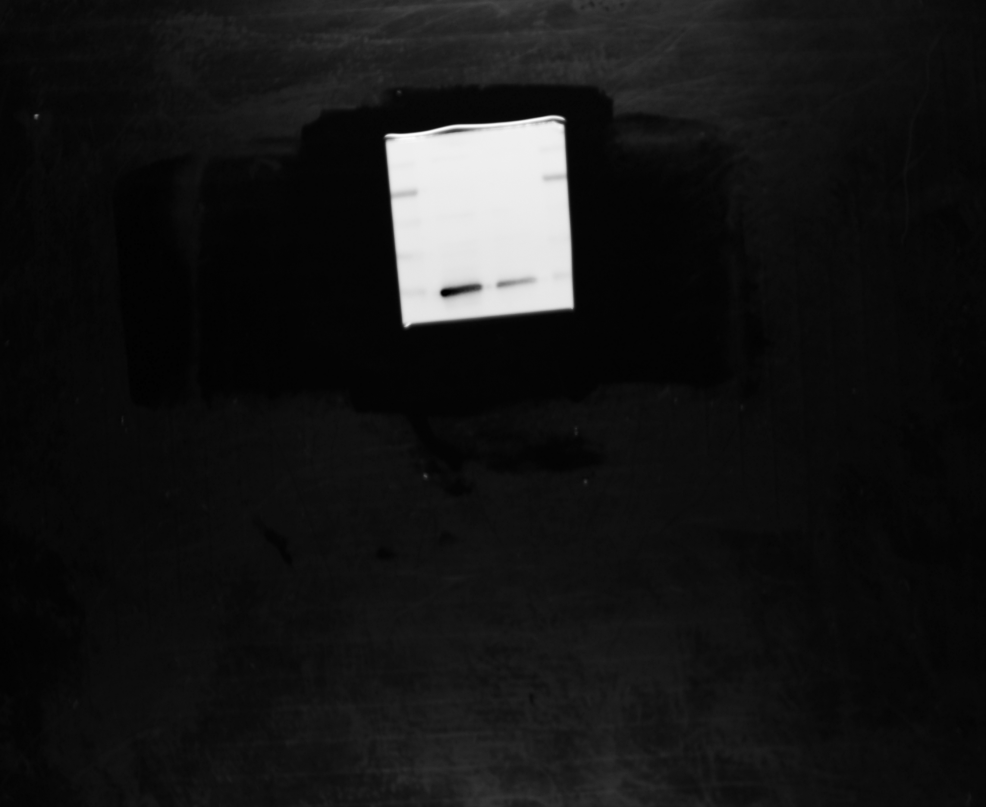


**KDa**

43

34

G3BP1


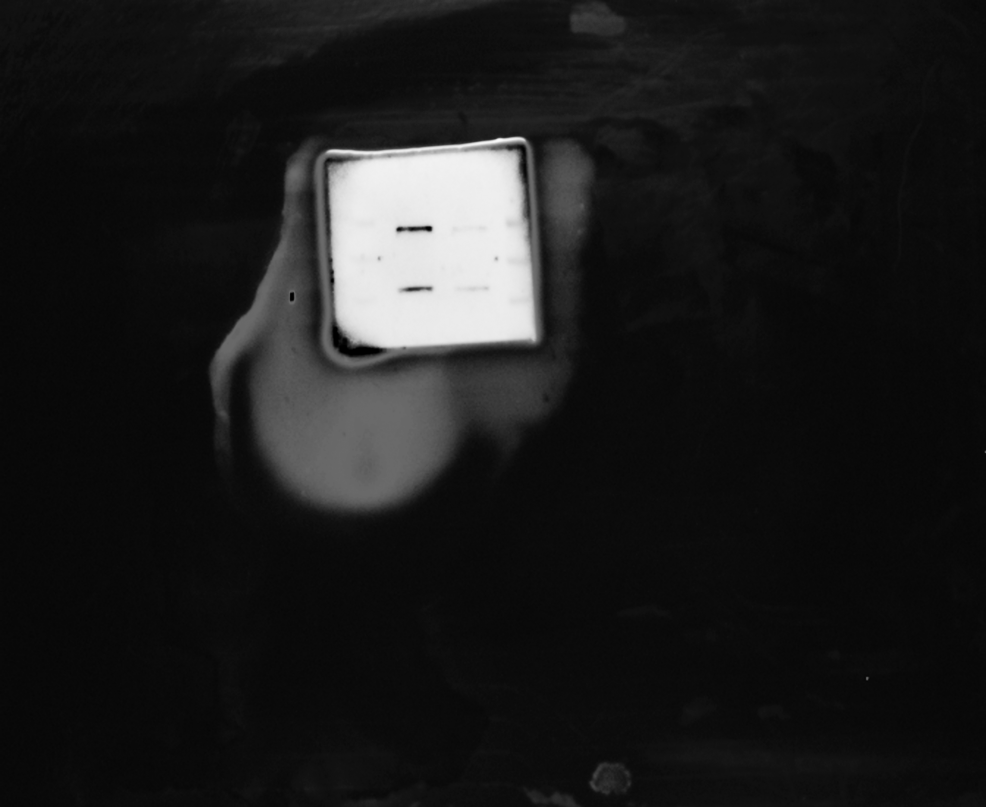


**KDa**

70

55

β-Actin


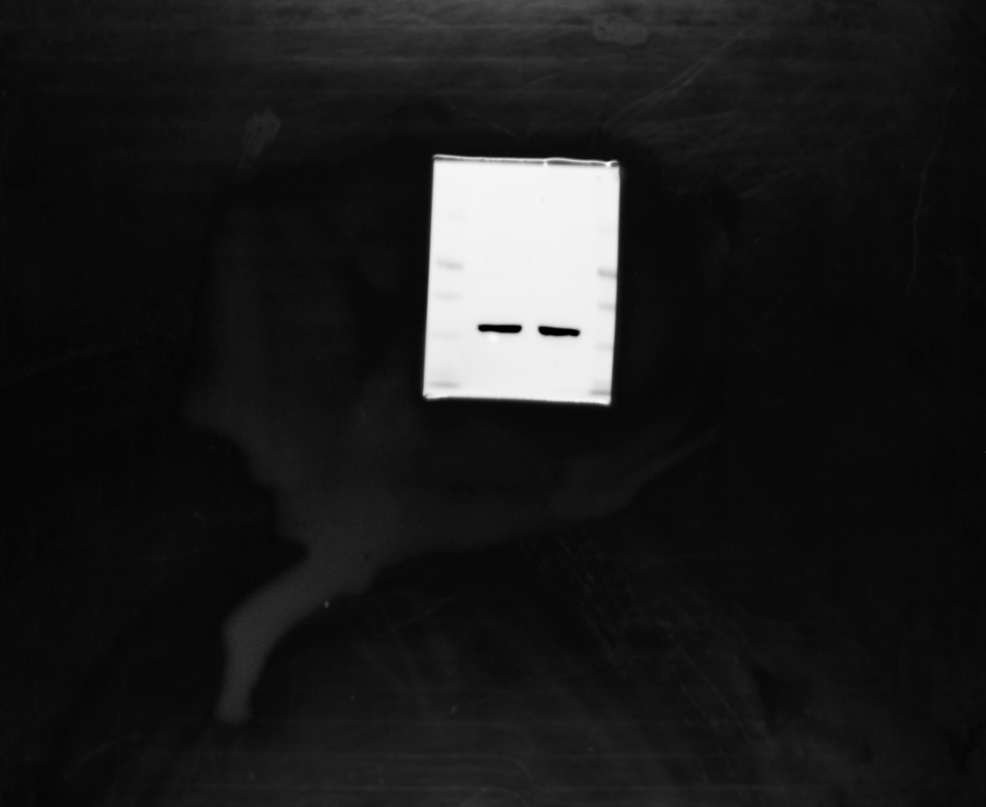


**KDa**

70

55

SOX2


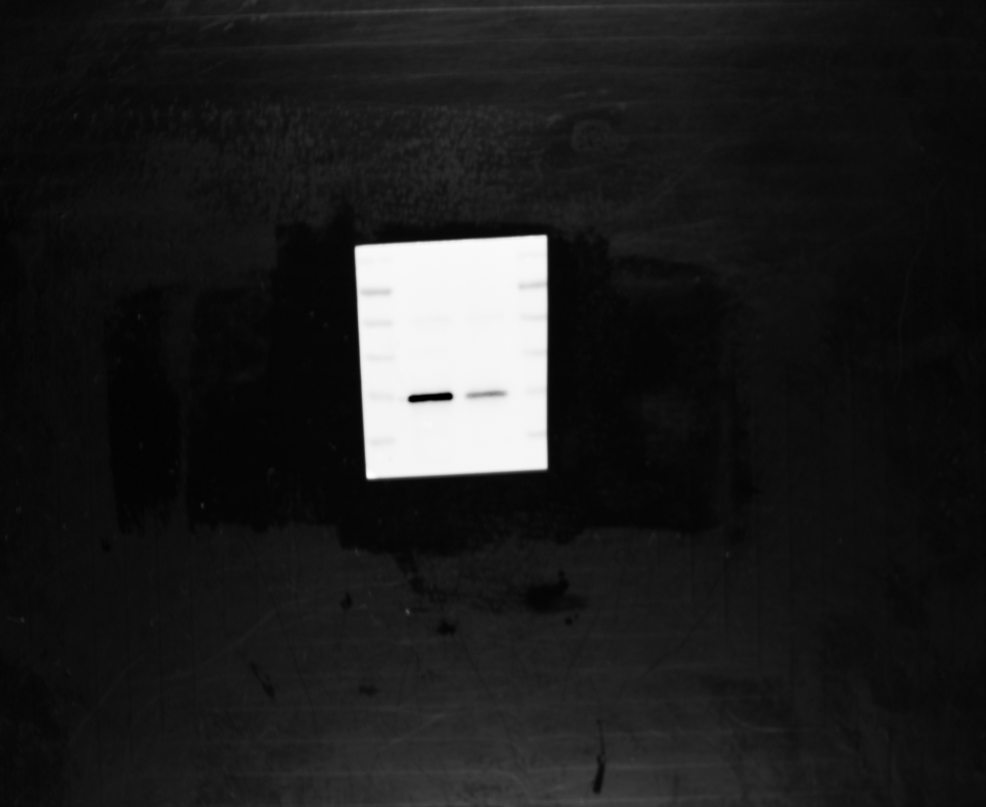


**KDa**

43

34

G3BP1


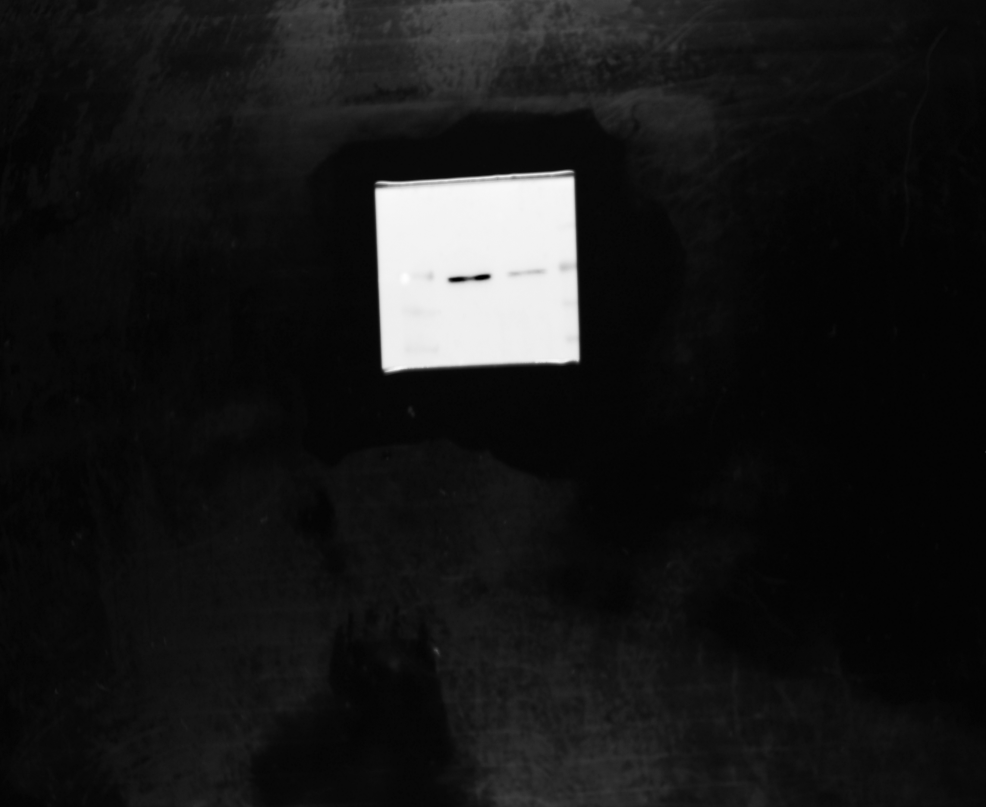


**KDa**

70

55

β-Actin


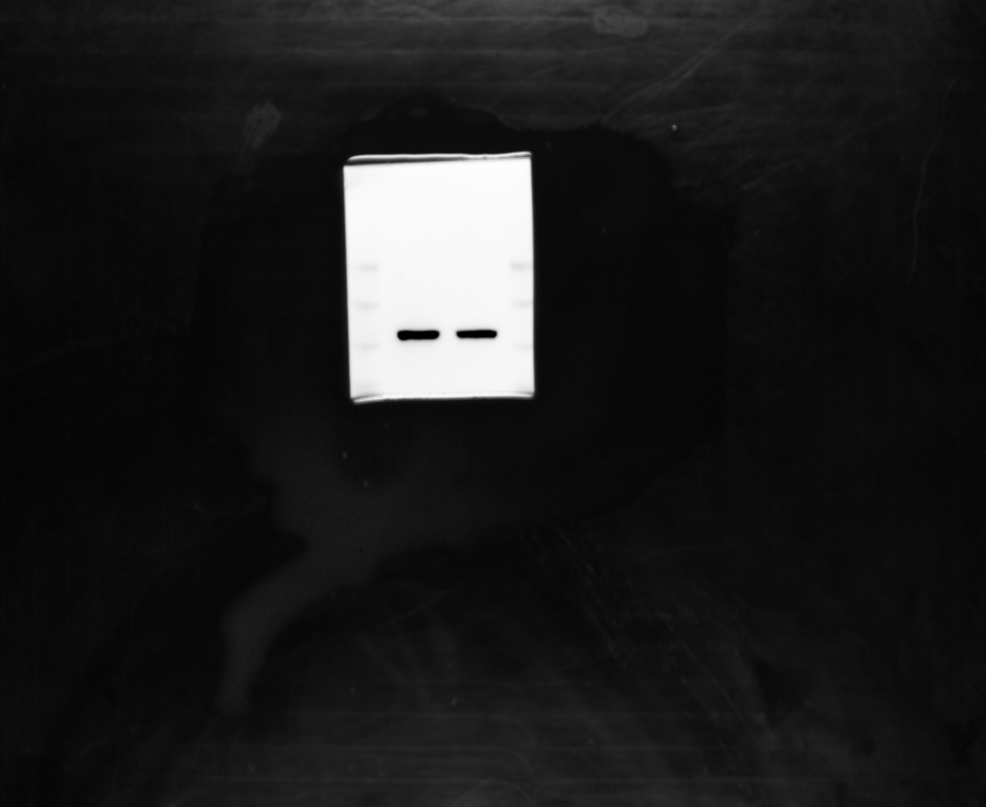


**KDa**

55

43

**Fig.7 H**

SOX2


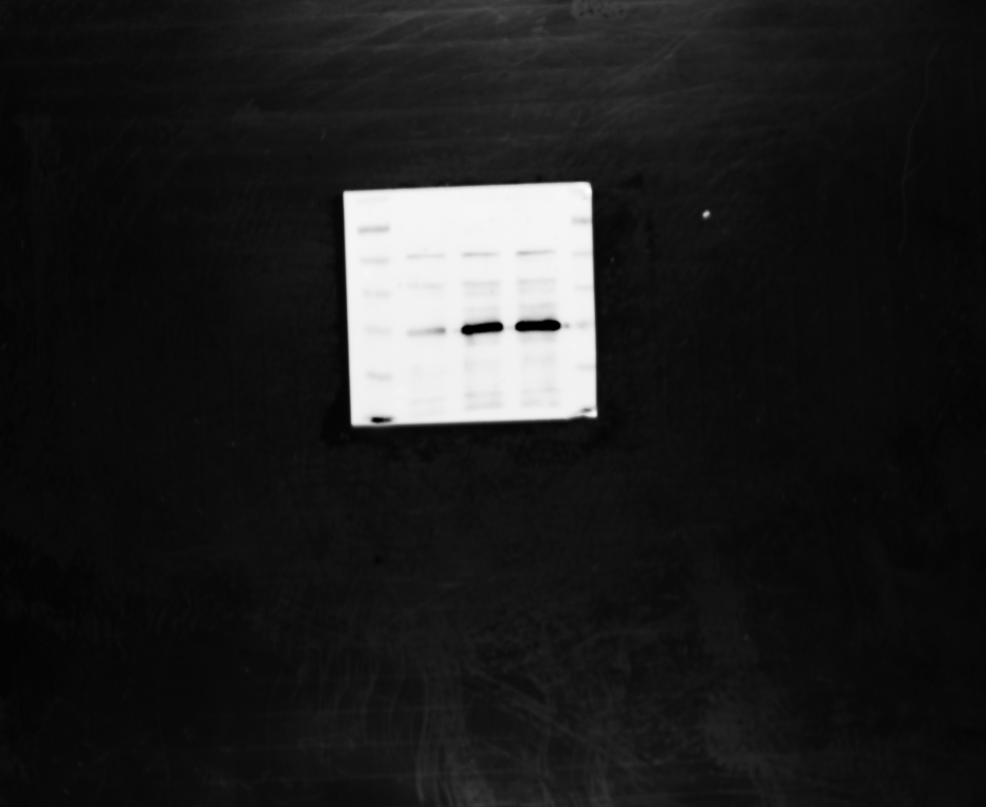


**KDa**

43

34

G3BP1


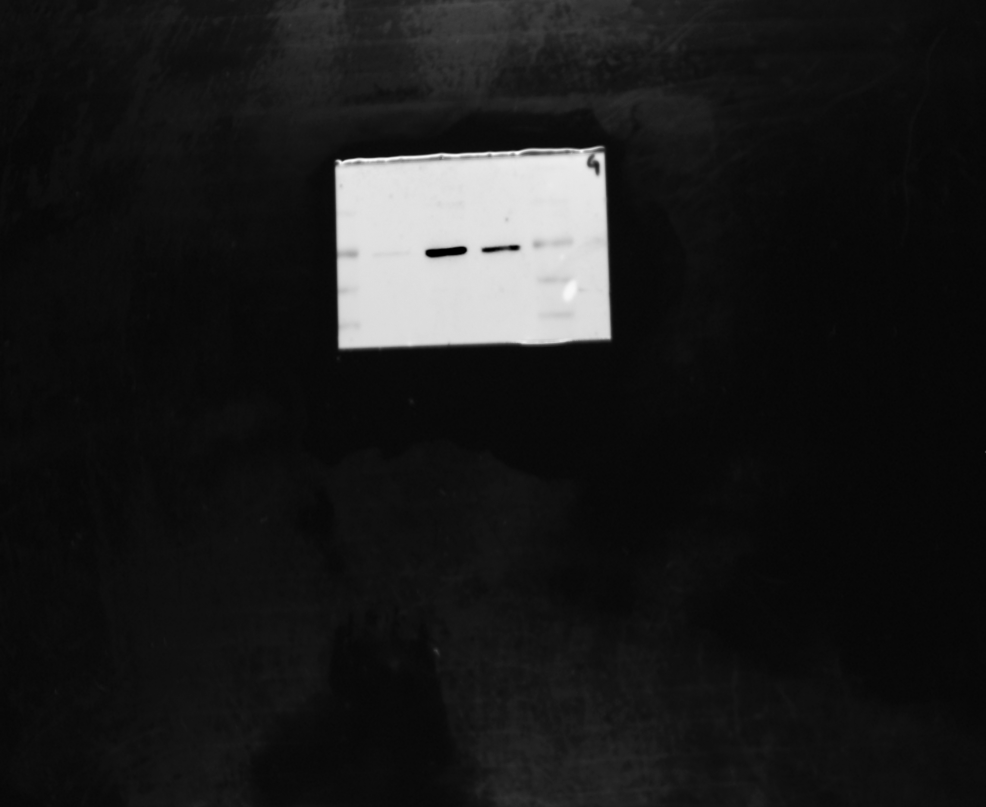


**KDa**

70

55

β-Actin


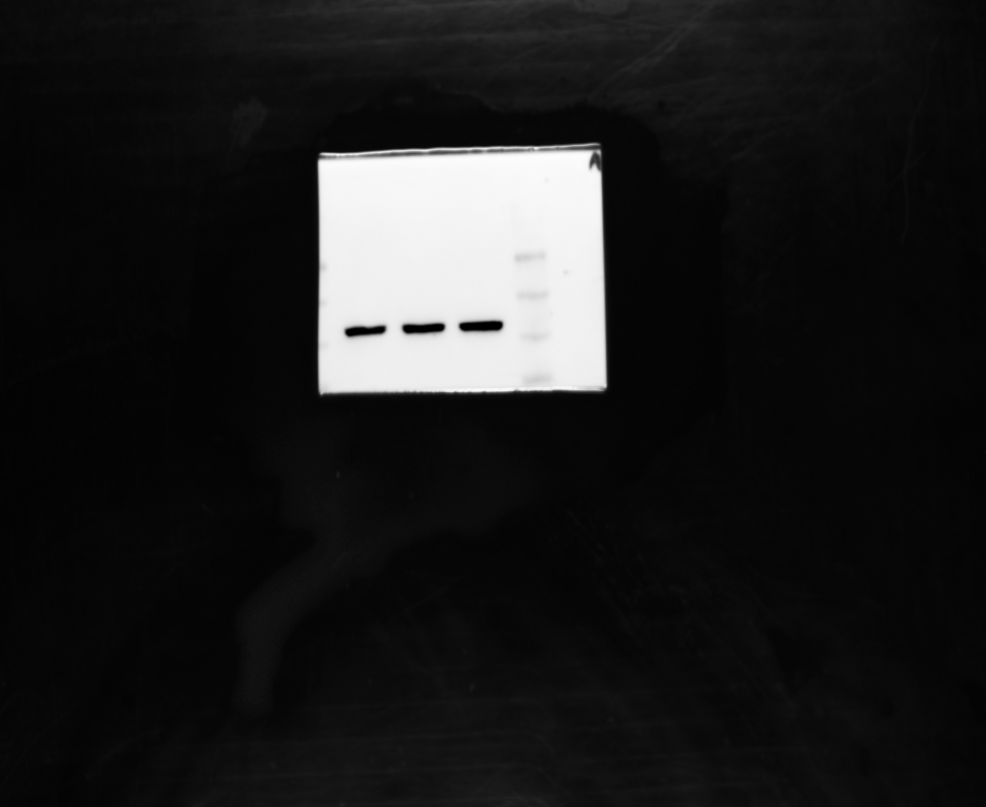


**KDa**

55

43

SOX2


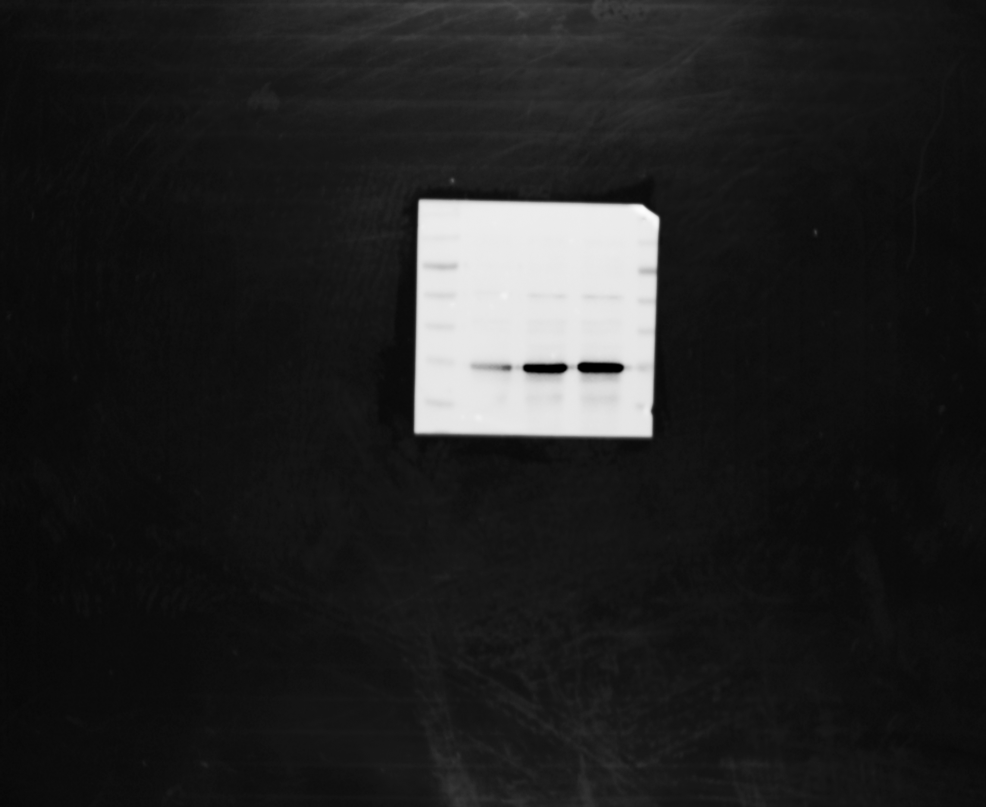


**KDa**

43

34

G3BP1


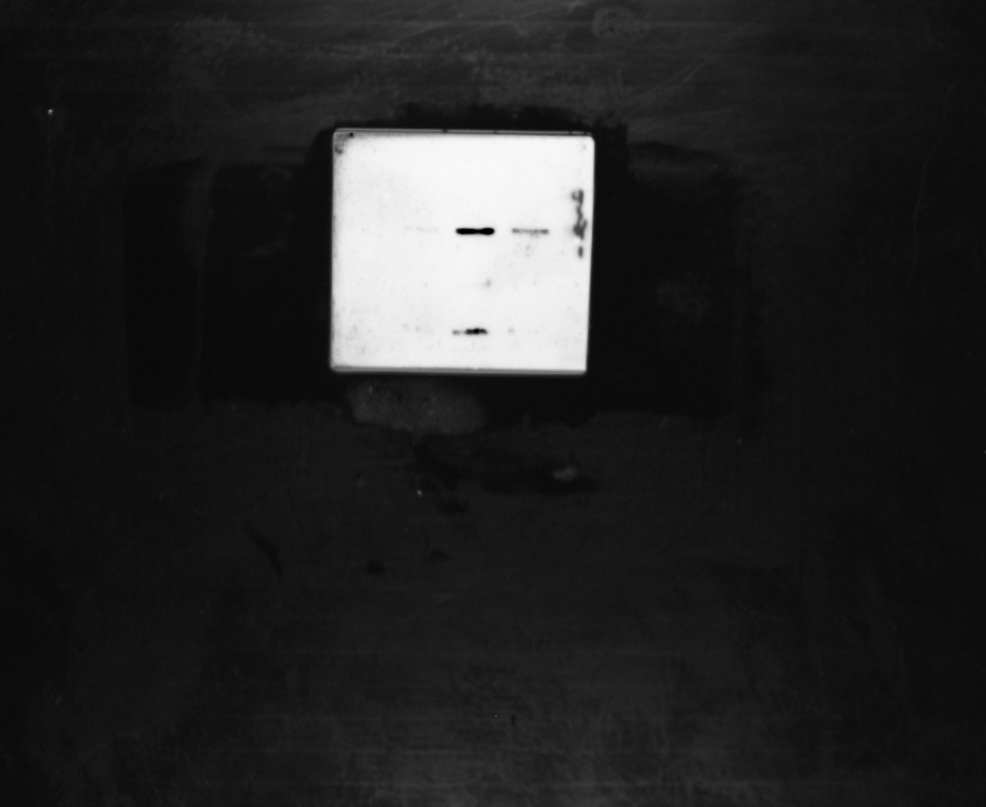


**KDa**

70

55

β-Actin


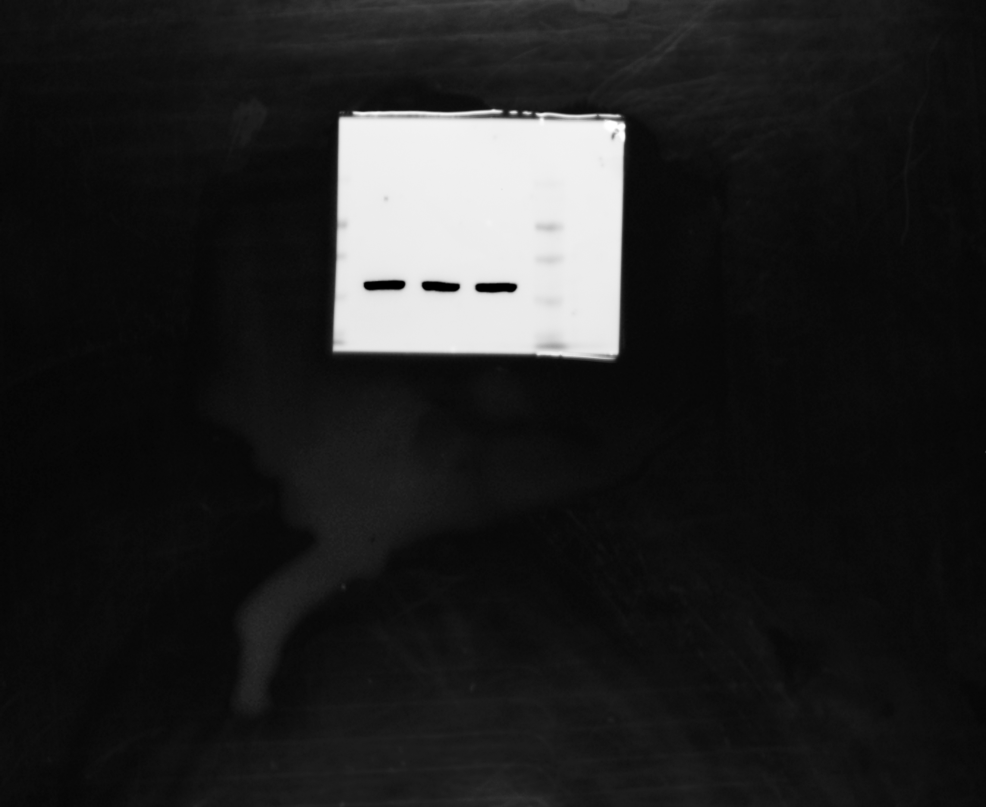


**KDa**

55

43

**Supplementary Fig.5 A**

**
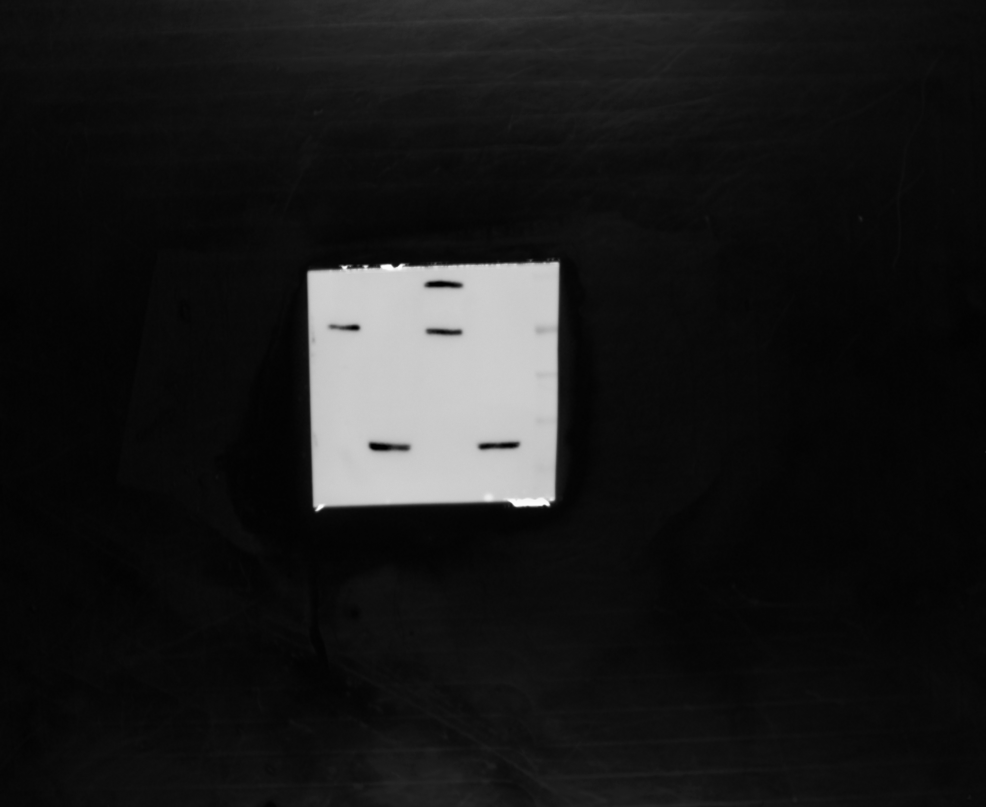
**

**KDa**

43

70

55

**Supplementary Fig.5 B**

**
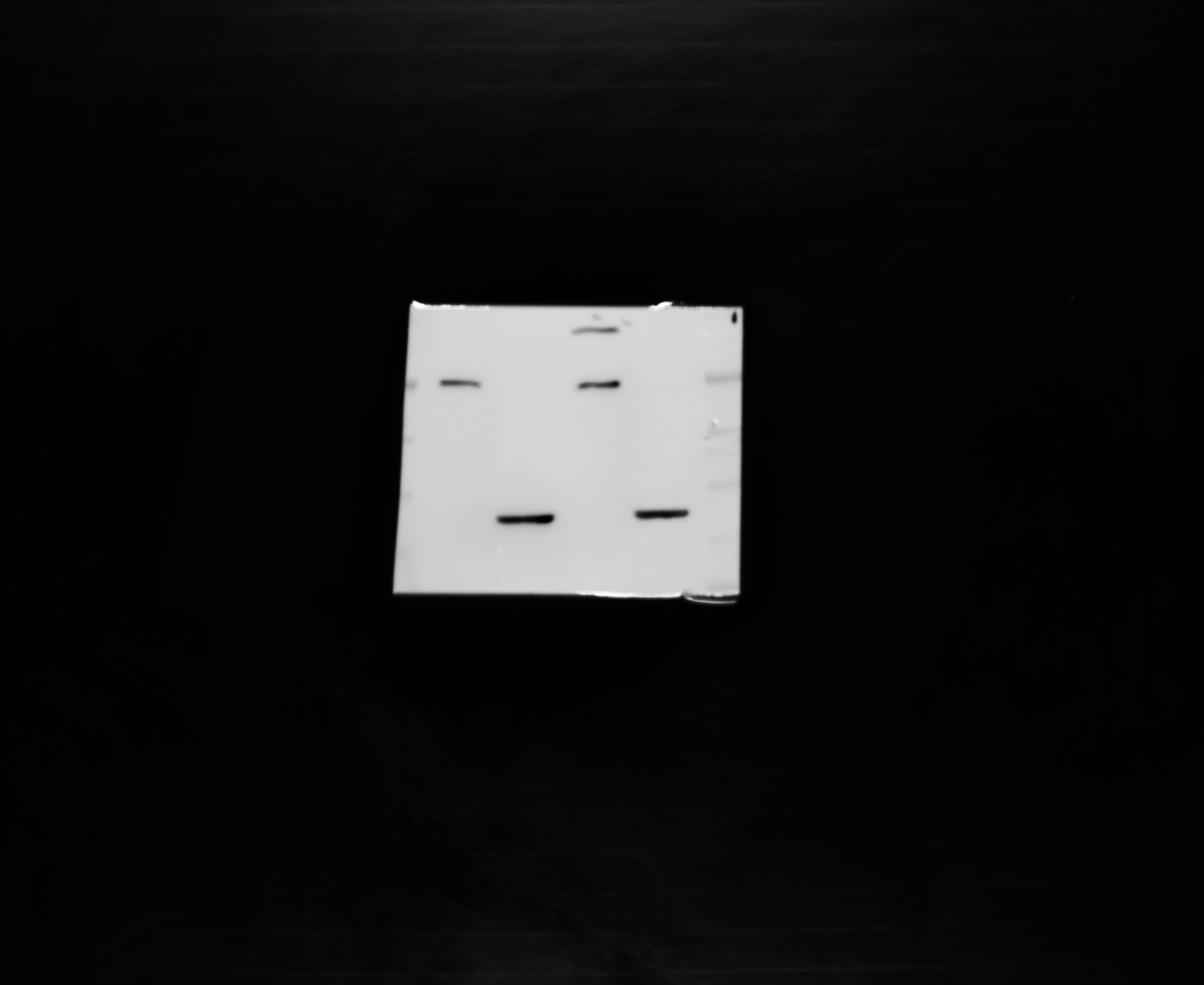
**

**KDa**

43

70

55

**Supplementary Fig.6 G**

γH2AX


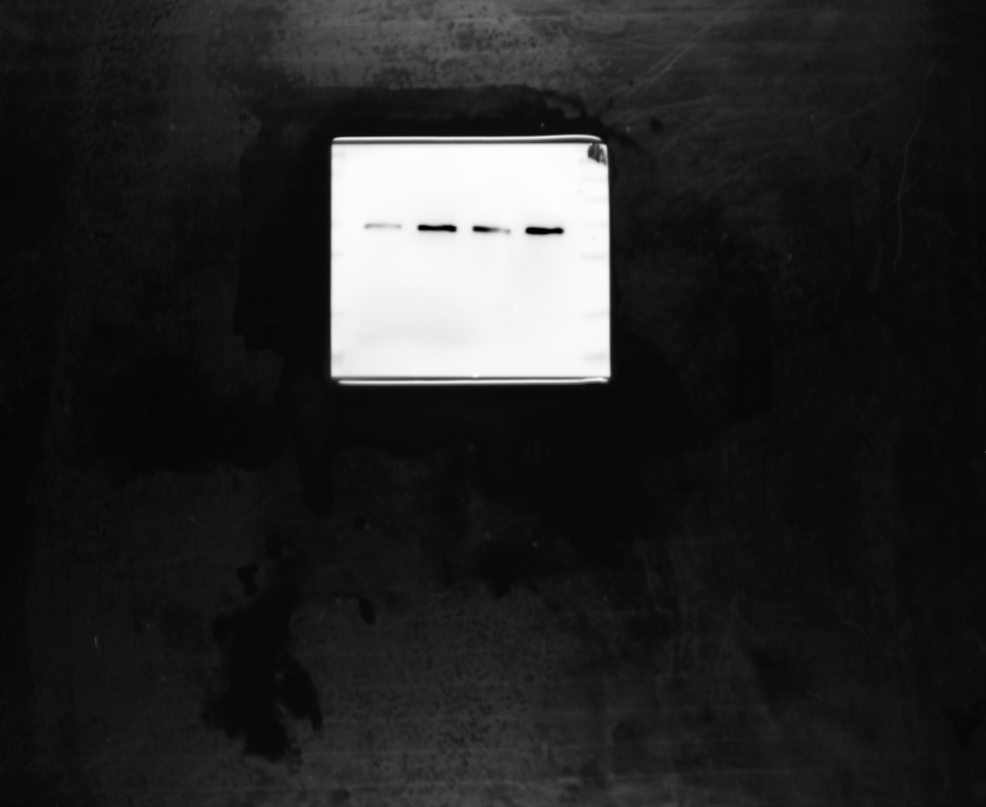


**KDa**

15

8

β-Actin


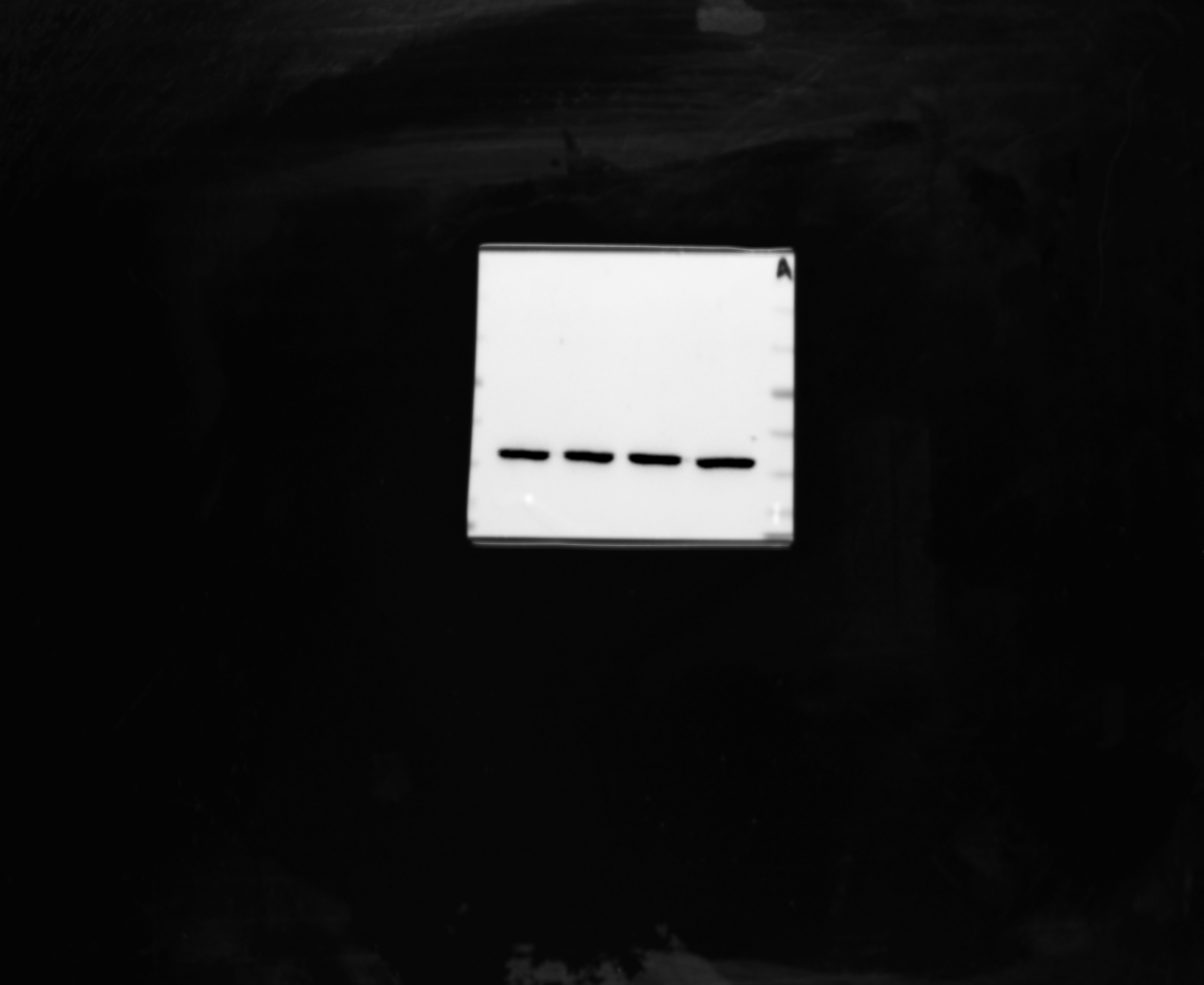


**KDa**

55

43

γH2AX


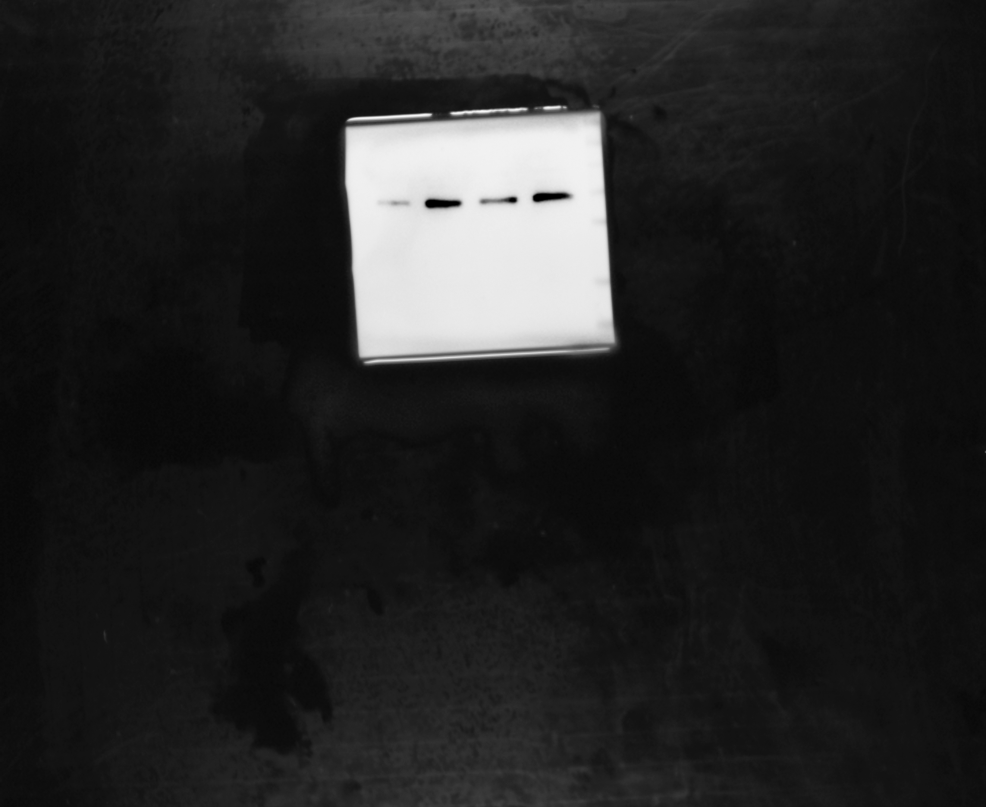


**KDa**

15

8

β-Actin


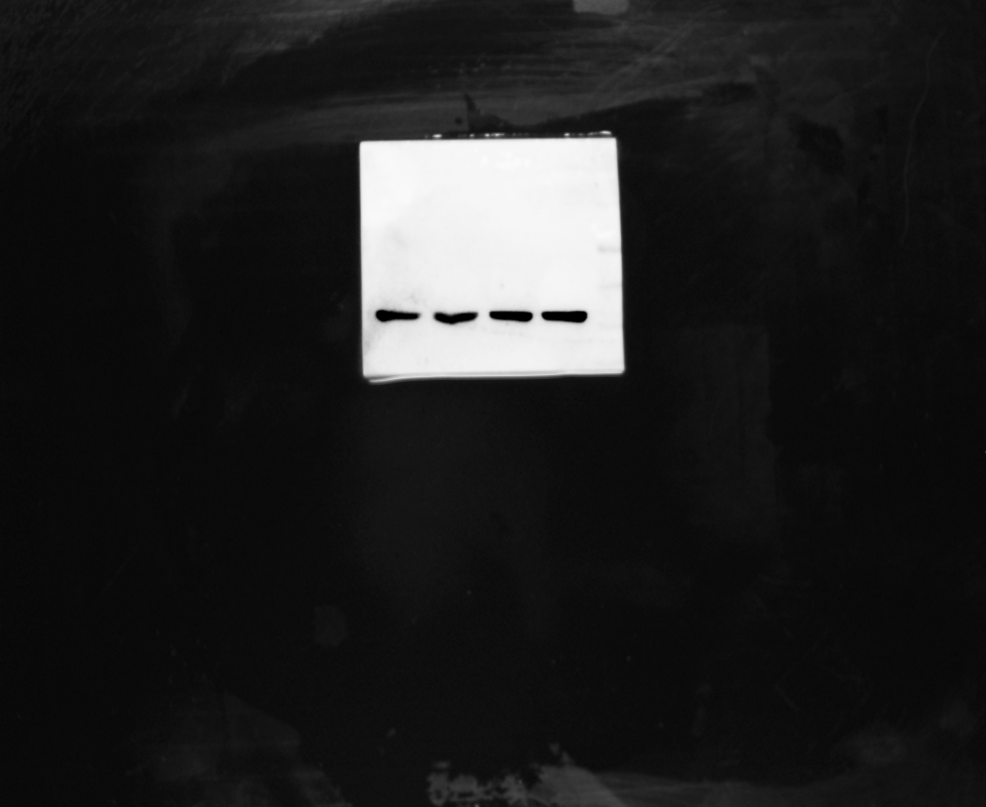


**KDa**

55

43

**Supplementary Fig.7 F**

γH2AX


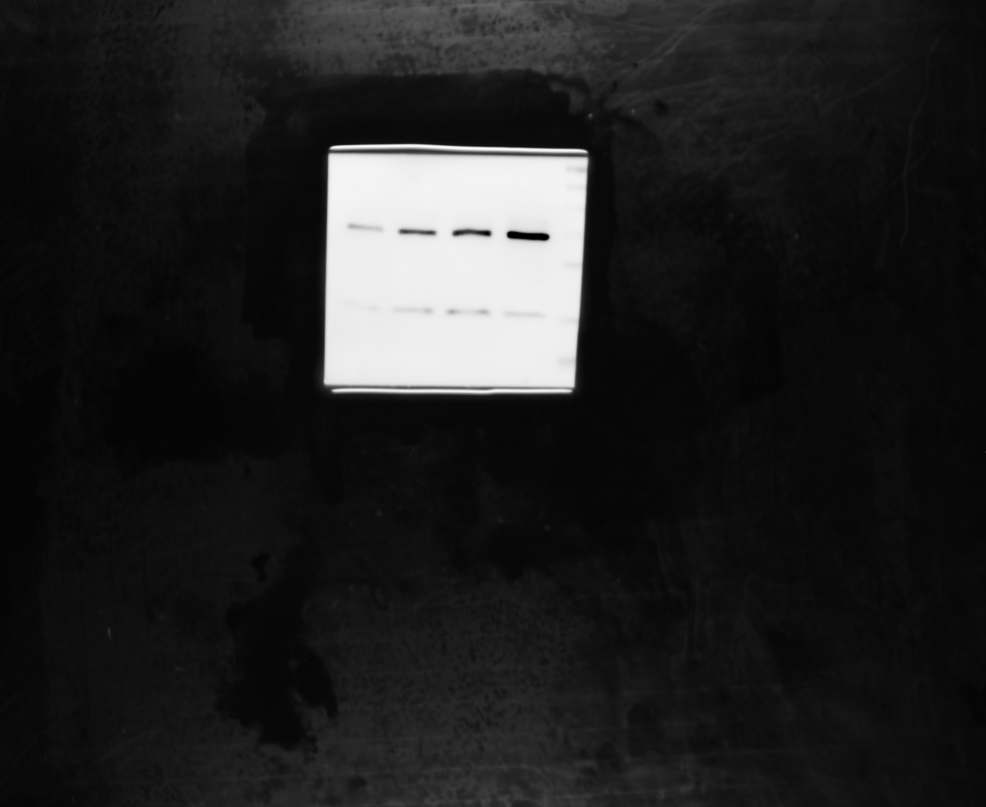


**KDa**

15

8

β-Actin


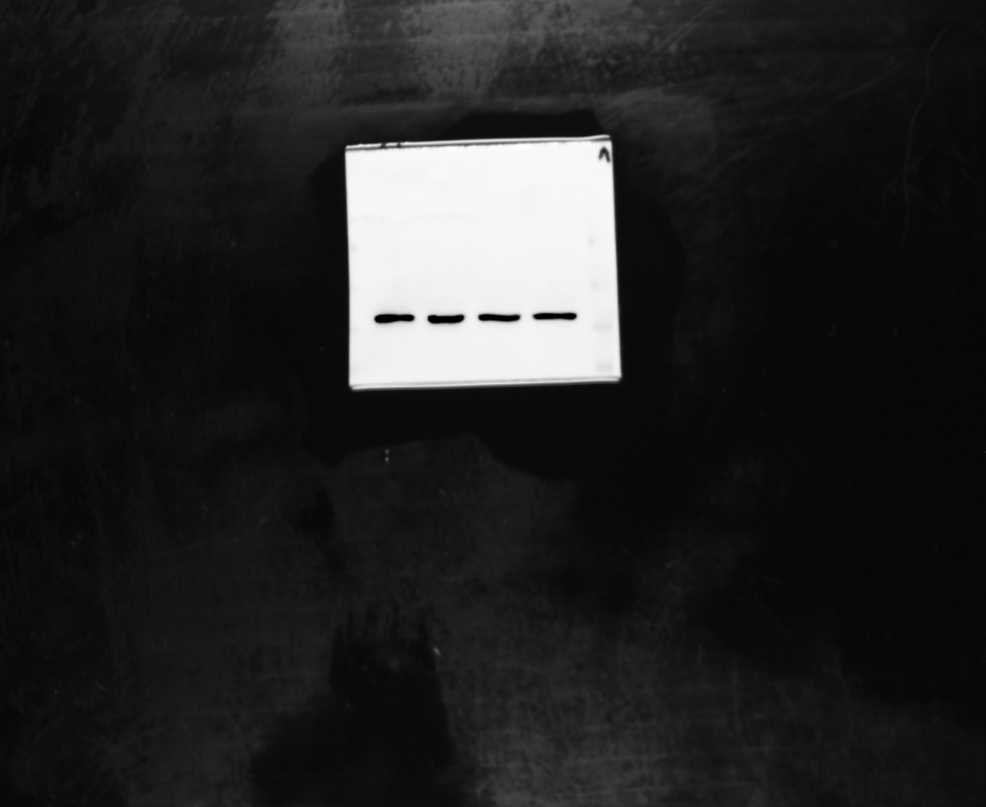


**KDa**

55

43

γH2AX


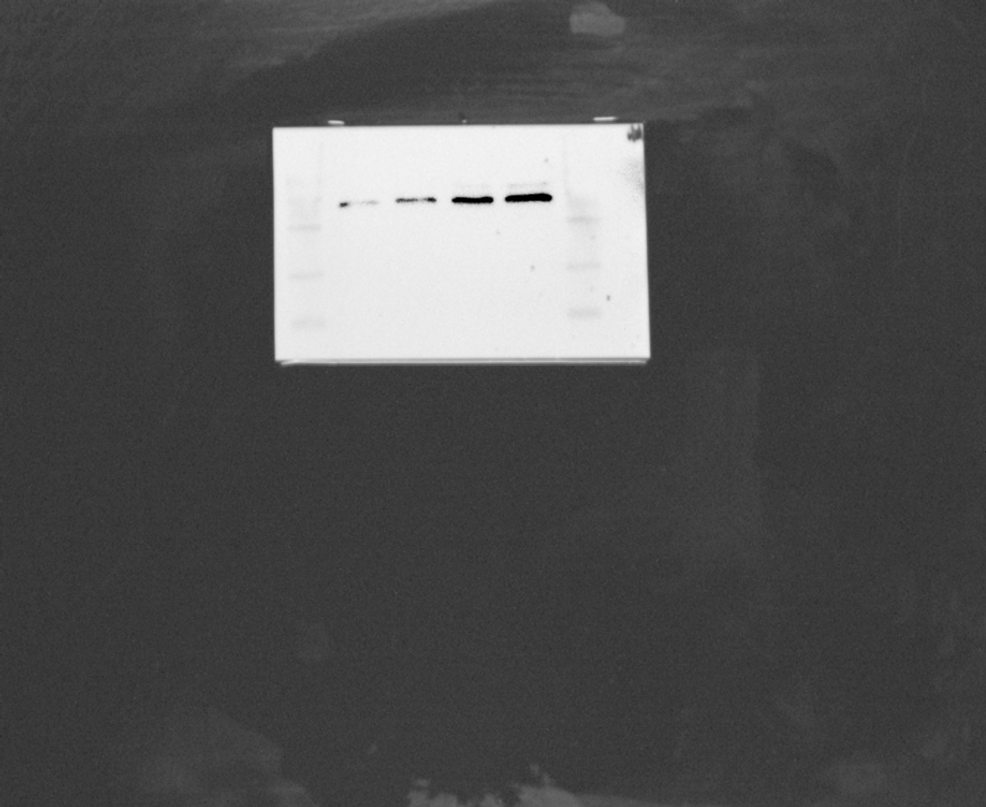


**KDa**

15

8

β-Actin


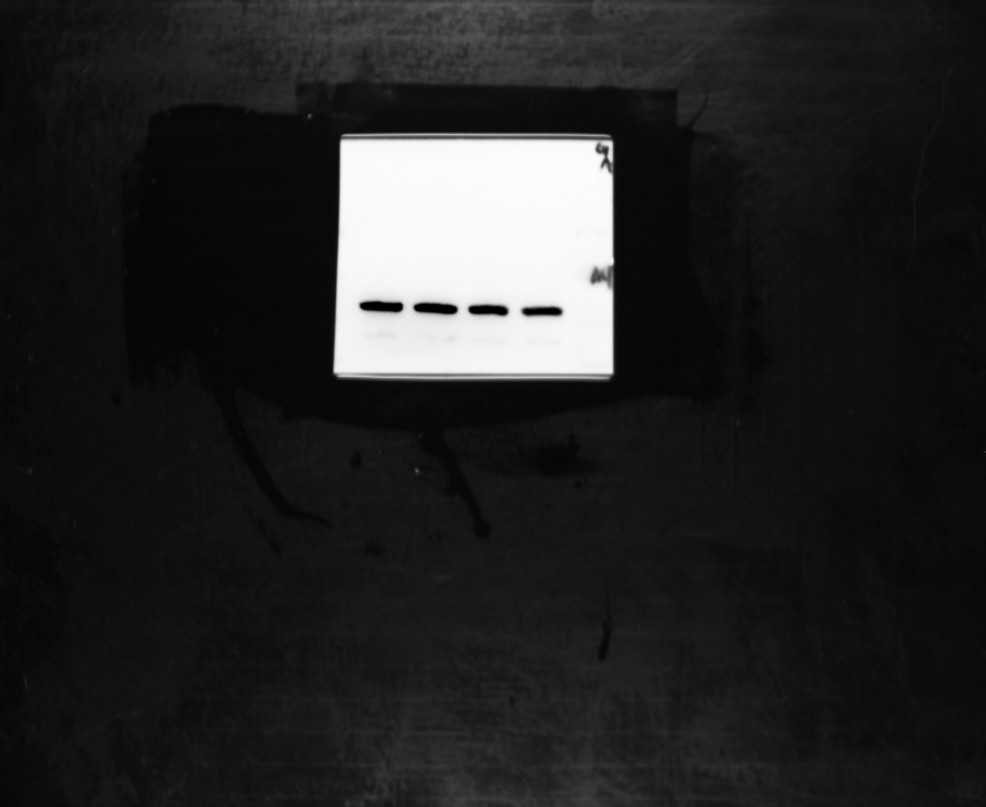


**KDa**

55

43

**Supplementary Fig.8 H**

G3BP1


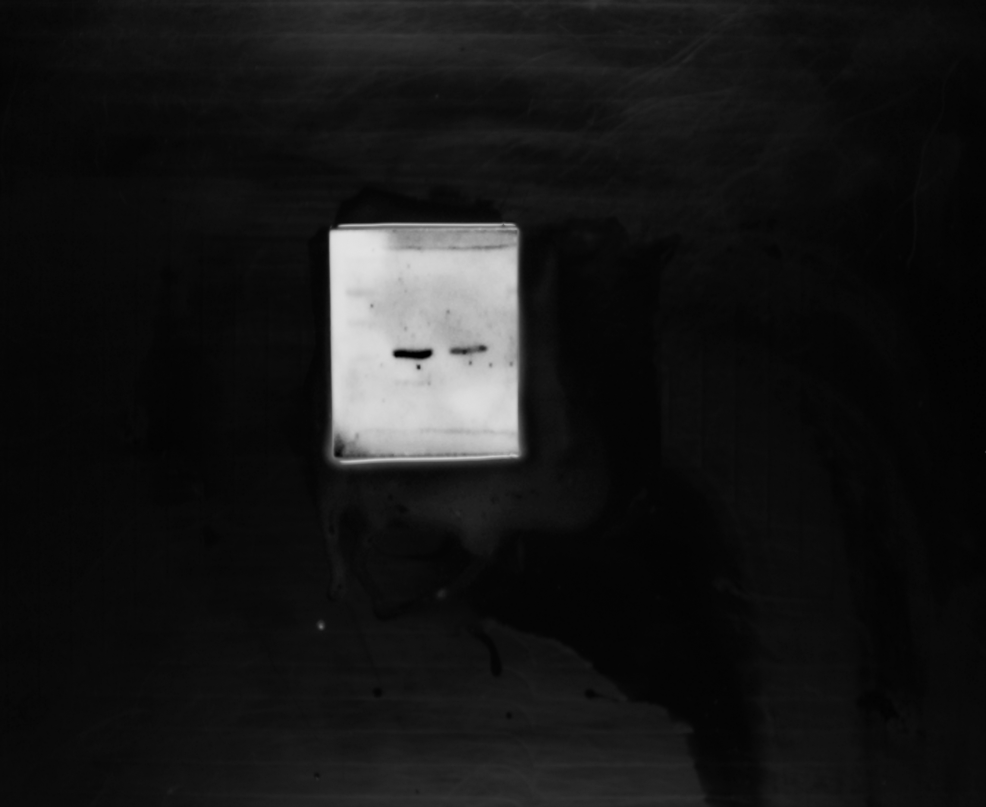


**KDa**

70

55

RAD51


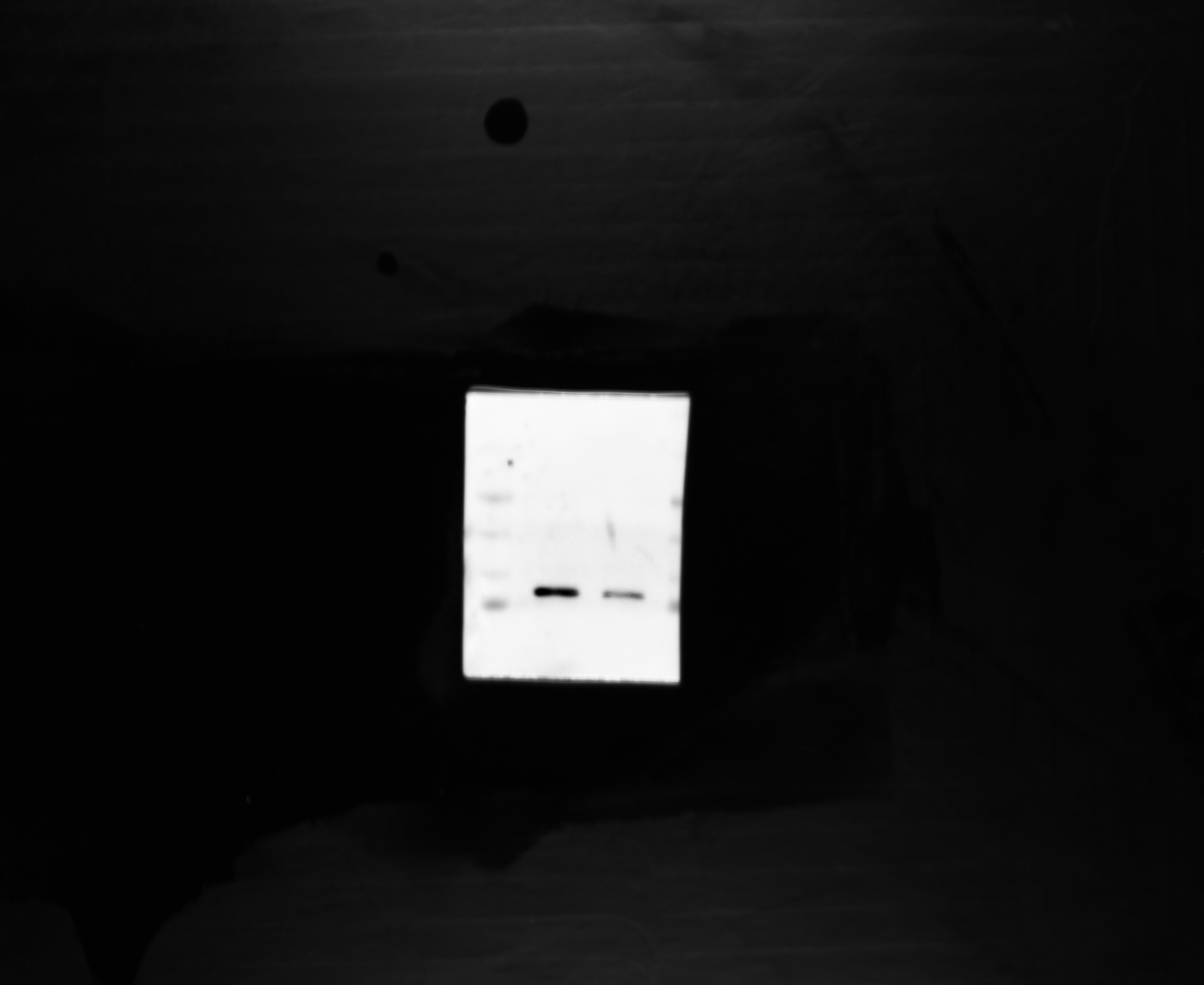


**KDa**

43

34

β-Actin


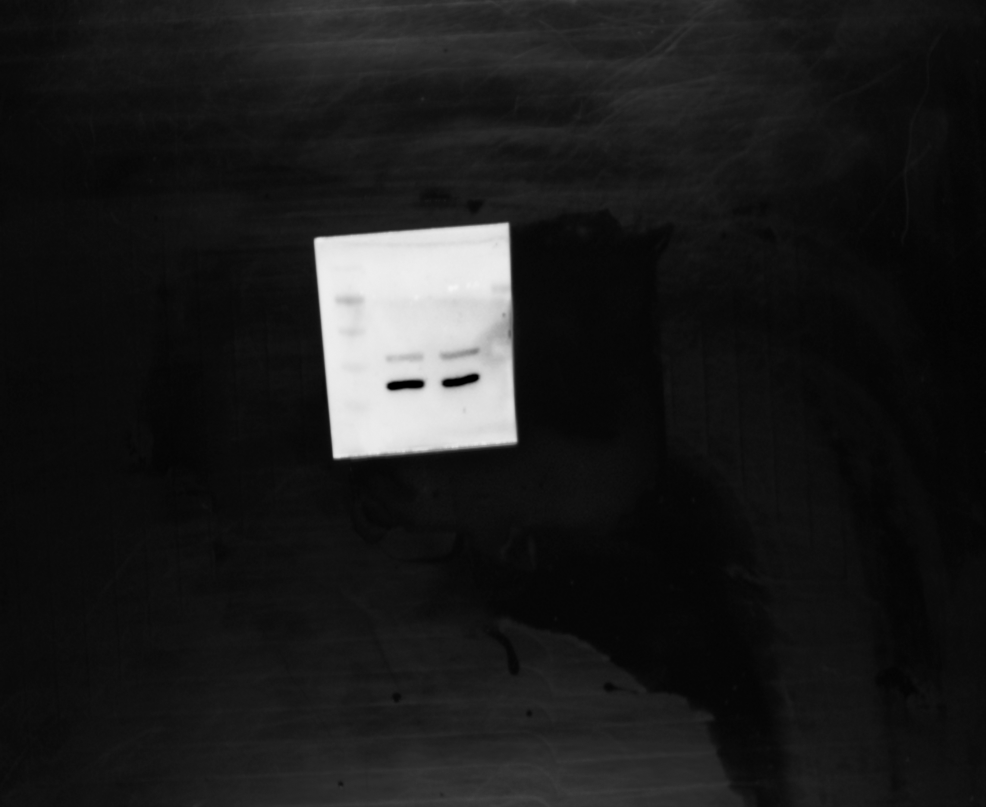


**KDa**

55

43

G3BP1


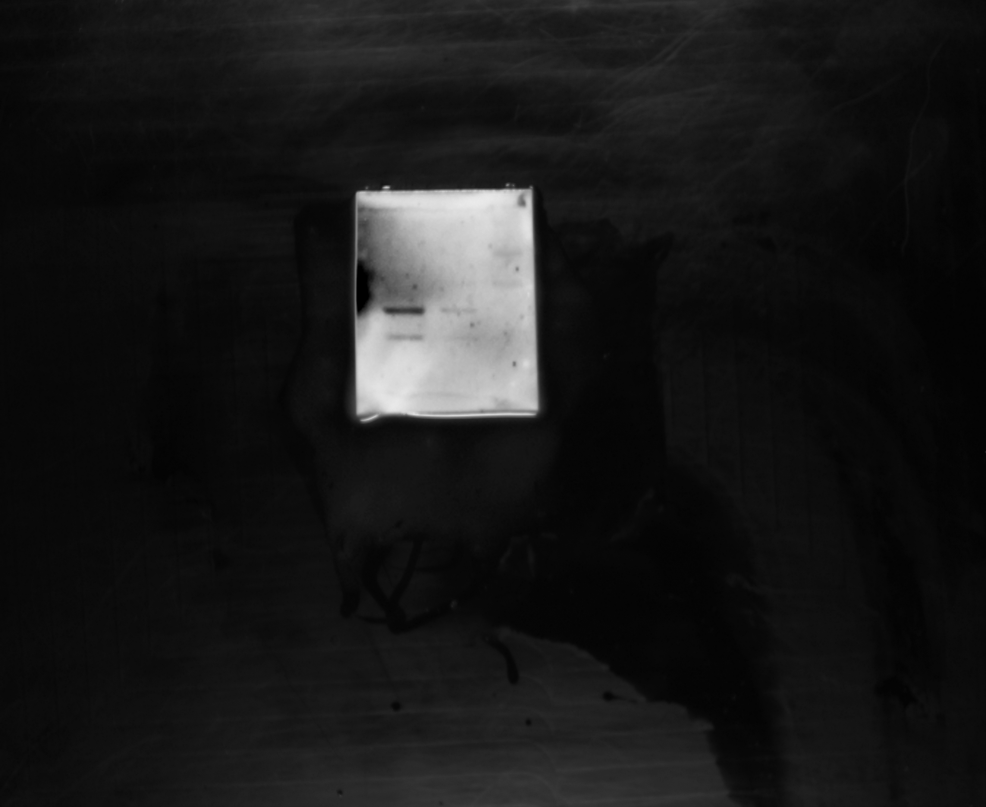


**KDa**

70

55

RAD51


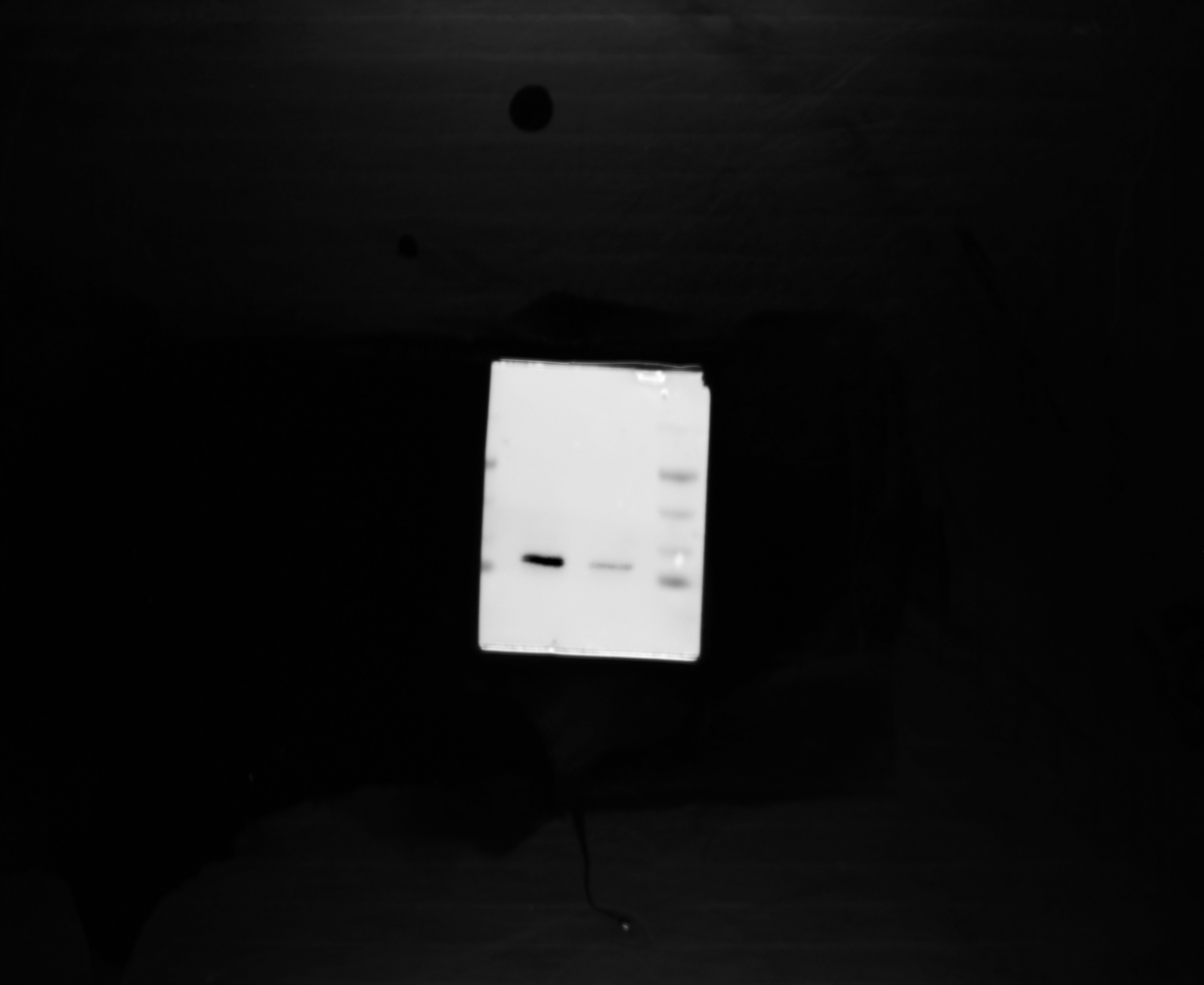


**KDa**

43

34

β-Actin


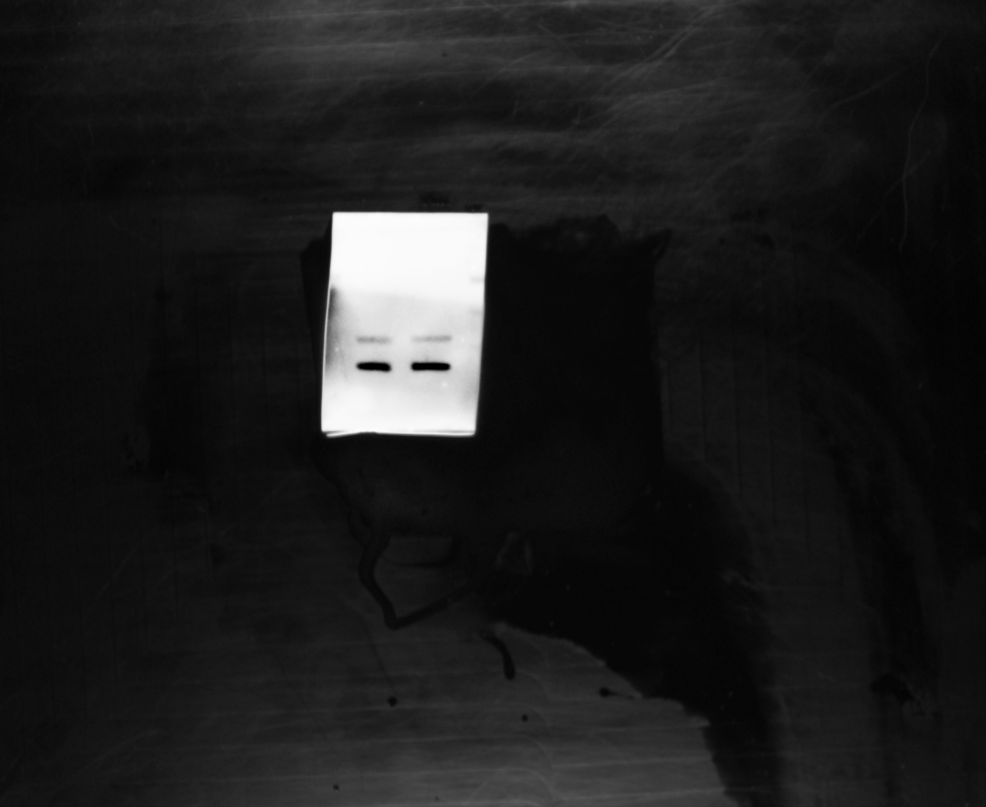


**KDa**

55

43
